# Discovery of Bacillamide–Acylhydrazone Hybrids as Novel Fungicide Lead Compounds

**DOI:** 10.3390/jof12030169

**Published:** 2026-02-26

**Authors:** Sijia Feng, Yuxiao Zhang, Peipei Shi, Ke Chen, Kang Lei

**Affiliations:** 1 College of Life Science, Henan Normal University, Xinxiang 453007, China; 2 School of Pharmaceutical Sciences and Food Engineering, Liaocheng University, Liaocheng 252059, China

**Keywords:** bacillamide, fungicide, acylhydrazone, copper ion transmembrane transporters

## Abstract

To identify fungicide lead compounds with novel scaffolds and high efficacy, 33 novel bacillamide–acylhydrazone derivatives were successfully designed and synthesized by using a molecular hybridization strategy. The bioassay results showed that most of the target compounds exhibited promising inhibitory activity against *B. cinerea*. Among them, compound **BAD-15** displayed the most potent antifungal activity with an EC_50_ value of 6.725 μg/mL. Furthermore, preliminary SAR analysis revealed that the R group in hydrazine fragments exerts a significant influence on antifungal potency. Studying the molecular mechanism by morphological observation and transcriptome analyses revealed that **BAD-15** may inhibit the activity of copper ion transmembrane transporters, leading to disrupted copper ion homeostasis and subsequent suppression of fungal growth. The present work indicates that bacillamide–acylhydrazone has potential as a novel scaffold for the development of fungicides, and compound **BAD-15** may serve as a potential lead compound for the further development of novel fungicides.

## Simple Summary

This study reports the design and synthesis of 33 novel bacillamide–acylhydrazone derivatives based on the structural features of natural products, aiming to identify novel antifungal lead scaffolds. Antifungal activity screening led to the discovery of a promising lead compound, **BAD-15**, whose mechanism of action was subsequently preliminarily investigated. The results suggest that compound **BAD-15** may inhibit copper ion transmembrane transporters, thereby disrupting copper homeostasis and ultimately suppressing fungal growth. This study establishes a theoretical foundation for the development of fungicides with novel modes of action, thereby contributing significant theoretical insights to antifungal agent research.

## 1. Introduction

Plant pathogenic fungi pose a significant threat to agricultural productivity, adversely affecting both crop quality and yield [1,2,3]. In modern agriculture, fungicides play a crucial role in the effective control and prevention of fungal infections in crops, thereby contributing to food security and sustainable agricultural development [4,5,6,7]. However, the prolonged and extensive use of fungicides has led to increasing resistance among plant pathogenic fungi, necessitating higher application rates and posing potential risks to non-target organisms and the environment [8,9,10]. Consequently, there is an urgent need for the development of novel fungicides with new molecular scaffolds or innovative modes of action.

**Figure 1:**
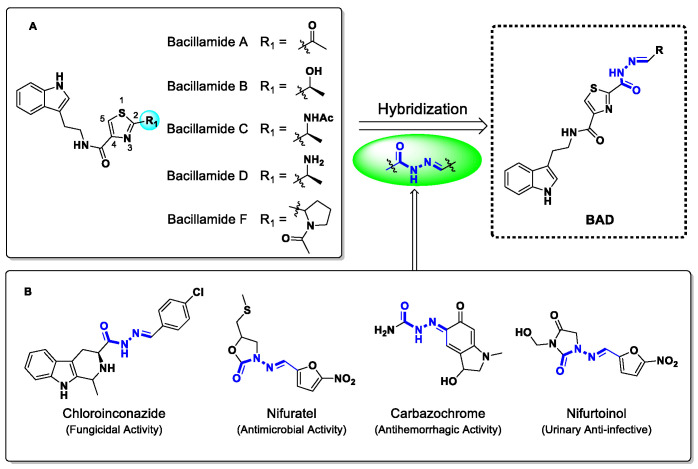
Design of target compound **BAD** by molecular hybridization strategy. (**A**): the common structures of natural bacillamides; (**B**) the structures of representative arylhydrazones with biological activity.

Natural products (NPs) constitute a rich and diverse reservoir of bioactive compounds, and their application in pesticide development holds substantial potential for the discovery of fungicides with novel modes of action, enhanced target specificity, and improved environmental compatibility [11]. For decades, extensive research has been devoted to the structural modification and derivatization of NPs in pursuit of antifungal compounds exhibiting high biological activity [12,13,14,15,16,17]. Bacillamides, which contain a tryptamide-thiazole structural motif, are a class of indole alkaloids produced by the *Bacillus genus* (Figure 1). Studies have demonstrated that natural bacillamides exhibit significant algicidal activity against a broad range of dinoflagellates, raphidophytes, and specific species of cyanobacteria [18,19], while bacillamide analogues display cytotoxic and anti-inflammatory activities [20], as well as inhibitory effects on HIV-1 replication [21]. However, the agricultural applications of natural bacillamides and their analogues remain limited. There has been no reported study on their antifungal activity to date.

It was easy to find that the structural variation among natural bacillamides is primarily attributed to the substituents located on the 2-position of the thiazole ring, and these differences in substituents account for the distinct biological activities exhibited by the compounds. On the other hand, acylhydrazones represent an important class of nitrogen-containing compounds in chemistry and have found extensive applications in the fields of agriculture and medicine [22]. Studies have demonstrated that acylhydrazones exhibit a wide range of biological activities, including antifungal [23,24,25], herbicidal [26,27], insecticidal [28], antimicrobial [29], antihemorrhagic [30], antitumor [31], antiviral [32], anti-inflammatory [33], and antiparasitic activities [34]. Based on these observations, we hypothesized that the introduction of an acylhydrazone moiety at the 2-position of thiazole ring in bacillamides could enable the construction of a series of bacillamide–acylhydrazone derivatives (**BAD**), which may exhibit promising agricultural biological activity. Therefore, as part of our ongoing efforts to discover novel antifungal lead compounds, we synthesized 33 bacillamide–acylhydrazone derivatives and evaluated their antifungal activities. To the best of our knowledge, this is the first report on the antifungal activity of bacillamide derivatives.

## 2. Materials and Methods

### 2.1. General Information

The raw materials were obtained from Shanghai Energy Chemical Co., Ltd. (Shanghai, China) or Shanghai Bide Pharmatech Co., Ltd. (Shanghai, China). All reagents were of analytical grade and were used as received without further purification. Melting points were measured using an X-4 digital electrothermal melting point apparatus (Beijing Tech Instruments Co., Beijing, China) and are reported as uncorrected values. Nuclear magnetic resonance (NMR) spectra were acquired on a Bruker AV500 spectrometer (Bruker Corp., Billerica, MA, USA) with deuterated DMSO as the solvent and tetramethylsilane (TMS) as the internal standard. Chemical shifts (δ) are expressed in parts per million (ppm). High-resolution mass spectra (HRMS) were obtained using a 6520 TOF LC/MS instrument (Agilent Technologies, Santa Clara, CA, USA).

### 2.2. Chemical Synthesis Procedures

**Scheme 1:**
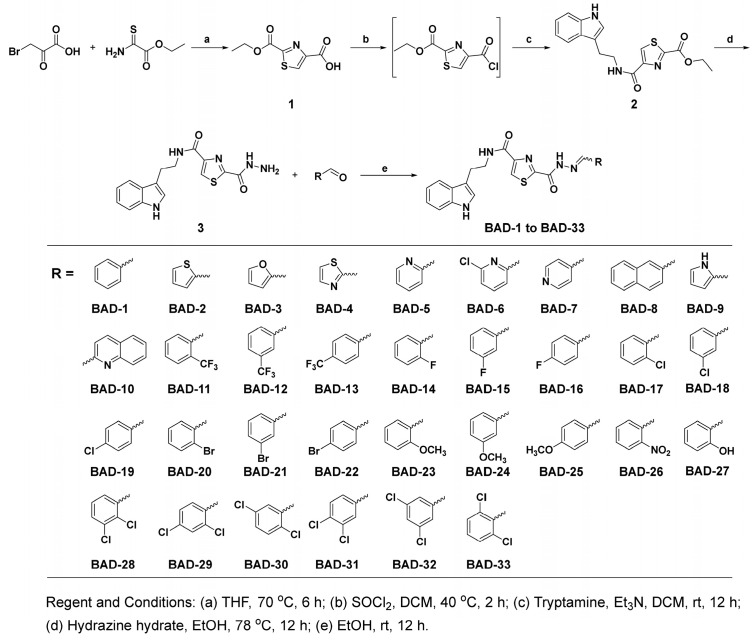
General synthetic route of the target compounds **BAD-1** to **BAD-33**.

The synthetic pathway employed for the preparation of the target compounds **BAD-1** to **BAD-33** is illustrated in Scheme 1. It should be noted that the yields have not been optimized, and the purity of the target compounds was over 95% according to the NMR.

#### 2.2.1. Method for the Synthesis of Intermediate **1**

Intermediate **1** was synthesized following a previously reported procedure [35]. Under a nitrogen atmosphere, a solution of 3-bromo-2-oxopropanoic acid (16.6 g, 100 mmol) and ethyl amino(thioxo)acetate (14.0 g, 105 mmol) in 300 mL anhydrous tetrahydrofuran (THF) was heated at 70 °C for 6 h. After that, the reaction mixture was reduced in vacuo to give an orange solid. The solid was triturated with ethyl acetate, filtered and dried in vacuo to give intermediate **1** (14.7 g, yield 73.1%) as a white solid. ^1^H NMR (500 MHz, DMSO-*d*_6_) δ 8.78 (s, 1H), 4.42 (q, *J* = 7.1 Hz, 2H), 1.37 (t, *J* = 7.1 Hz, 3H); ^13^C NMR (126 MHz, DMSO-*d*_6_) δ 162.1 (s), 159.6 (s), 158.4 (s), 149.2 (s), 134.5 (s), 62.9 (s), 14.4 (s).

#### 2.2.2. Method for the Synthesis of Intermediate **2**

A 250 mL round-bottom flask was filled with intermediate **1** (10.0 g, 50 mmol) and dichloromethane (DCM, 100 mL). Thionyl chloride (SOCl_2_, 20 mL) was added dropwise to the stirred mixture, followed by the addition of one drop of *N*, *N*-dimethylformamide (DMF). The reaction mixture was heated at 40 °C for 2 h. Upon completion, the solvent and excess thionyl chloride were removed under reduced pressure using a rotary evaporator, and the residue was redissolved in 100 mL of fresh DCM. Subsequently, tryptamine (8.0 g, 50 mmol) and triethylamine (Et_3_N, 10.1 g, 100 mmol) were added to the solution. The resulting mixture was stirred at room temperature for 12 h. After reaction completion, as confirmed by thin-layer chromatography (TLC), the solvent was removed under reduced pressure, and the residue was recrystallized from ethyl acetate to afford intermediate **2** (12.1 g, 70.3% yield) as a white solid. ^1^H NMR (500 MHz, DMSO-*d*_6_) δ 10.80 (s, 1H), 8.65 (t, *J* = 6.0 Hz, 1H), 7.62 (d, *J* = 7.9 Hz, 1H), 7.34 (d, *J* = 8.1 Hz, 1H), 7.18 (d, *J* = 2.2 Hz, 1H), 7.11–7.05 (m, 1H), 7.01–6.94 (m, 1H), 4.42 (q, *J* = 7.1 Hz, 2H), 3.58 (dd, *J* = 14.8, 6.5 Hz, 2H), 3.06–2.85 (m, 2H), 1.35 (t, *J* = 7.1 Hz, 3H); ^13^C NMR (126 MHz, DMSO-*d*_6_) δ 160.2 (s), 159.5 (s), 158.0 (s), 152.2 (s), 136.7 (s), 130.3 (s), 127.7 (s), 123.0 (s), 121.4 (s), 118.8 (s), 118.6 (s), 112.1 (s), 111.8 (s), 62.9 (s), 25.74 (s), 14.5 (s).

#### 2.2.3. Method for the Synthesis of Intermediate **3**

To a 250 mL round-bottom flask, intermediate **2** (10.3 g, 30 mmol) was added, followed by the sequential addition of 100 mL of ethanol and 3 mL of hydrazine hydrate (80 wt%). The reaction mixture was stirred at 78 °C for 12 h. After cooling to room temperature, the mixture was stored at 4 °C in a refrigerator overnight to promote precipitation. The resulting solid was collected by filtration and dried to afford intermediate **3** (8.7 g, 88.1% yield) as a white solid. ^1^H NMR (500 MHz, DMSO-*d*_6_) δ 10.85 (s, 1H), 10.04 (s, 1H), 8.62 (t, J = 5.8 Hz, 1H), 8.48 (s, 1H), 7.60 (d, *J* = 7.9 Hz, 1H), 7.35 (d, *J* = 8.1 Hz, 1H), 7.22 (d, *J* = 1.8 Hz, 1H), 7.08 (t, *J* = 7.5 Hz, 1H), 6.99 (t, *J* = 7.4 Hz, 1H), 4.73 (s, 2H), 3.61 (q, *J* = 6.9 Hz, 2H), 2.99 (t, *J* = 7.4 Hz, 2H); ^13^C NMR (126 MHz, DMSO-*d*_6_) δ 162.1 (s), 160.4 (s), 158.5 (s), 150.5 (s), 136.7 (s), 127.8 (s), 127.6 (s), 123.1 (s), 121.4 (s), 118.7 (s), 118.7 (s), 111.9 (s), 111.8 (s), 40.0 (s), 25.7 (s).

#### 2.2.4. General Method for the Synthesis of Target Compounds **BAD-1** to **BAD-33**

Intermediate **3** (329 mg, 1.0 mmol), benzaldehyde (117 mg, 1.1 mmol), and 10 mL of ethanol were added to a 50 mL round-bottom flask. The reaction mixture was stirred at room temperature for 12 h. The resulting precipitate was collected by filtration and dried to afford target compound **BAD-1** (385 mg, 92.3% yield) as a white solid. Target compounds **BAD-2** to **BAD-33** were prepared using an analogous procedure.

The detailed physiochemical information, yields, ^1^H NMR, ^13^C NMR, and HRMS of all target compounds are shown below. It should be noted that the ^13^C NMR signal assigned to the aliphatic carbon adjacent to the tryptophan amide (δ ≈ 40 ppm) becomes blurred due to overlap with the resonance of the DMSO-*d*_6_ solvent.

*N*-(2-(1*H*-indol-3-yl)ethyl)-2-(2-benzylidenehydrazine-1-carbonyl)thiazole-4-carboxamide (**BAD-1**): White solid; yield 92.3%; M.p. 242–243 °C; ^1^H NMR (500 MHz, DMSO-*d*_6_) δ 12.05 (s, 1H), 10.85 (s, 1H), 8.60 (s, 1H), 8.59 (s, 1H), 8.55 (t, *J* = 6.0 Hz, 1H), 7.79–7.77 (m, 2H), 7.61 (d, *J* = 7.8 Hz, 1H), 7.53–7.46 (m, 3H), 7.35 (d, *J* = 8.1 Hz, 1H), 7.22 (d, *J* = 2.2 Hz, 1H), 7.11–7.03 (m, 1H), 7.02–6.95 (m, 1H), 3.67–3.63 (m, 2H), 3.02 (t, *J* = 7.5 Hz, 2H); ^13^C NMR (126 MHz, DMSO-*d*_6_) δ 162.4 (s), 160.4 (s), 155.7 (s), 150.9 (s), 150.6 (s), 136.7 (s), 134.2 (s), 131.1 (s), 129.4 (s), 129.2 (s), 127.8 (s), 127.6 (s), 123.2 (s), 121.4 (s), 118.7 (s), 111.9 (s), 111.9 (s), 25.8 (s); HRMS, *m*/*z* calcd. for C_22_H_20_N_5_O_2_S^+^ [M+H]^+^ 418.1332, found 418.1330.

*N*-(2-(1*H*-indol-3-yl)ethyl)-2-(2-(thiophen-2-ylmethylene)hydrazine-1-carbonyl)thiazole-4-carboxamide (**BAD-2**): White solid; yield, 83.9%; M.p. 232–233 °C; ^1^H NMR (500 MHz, DMSO-*d*_6_) δ 12.05 (s, 1H), 10.84 (s, 1H), 8.81 (s, 1H), 8.58 (s, 1H), 8.51 (t, *J* = 6.0 Hz, 1H), 7.74 (d, *J* = 5.0 Hz, 1H), 7.61 (d, *J* = 7.9 Hz, 1H), 7.56–7.52 (m, 1H), 7.35 (d, *J* = 8.1 Hz, 1H), 7.22 (d, *J* = 2.1 Hz, 1H), 7.18 (dd, *J* = 5.0, 3.6 Hz, 1H), 7.07 (dd, *J* = 11.1, 3.9 Hz, 1H), 6.99 (dd, *J* = 11.0, 3.9 Hz, 1H), 3.66–3.62 (m, 2H), 3.01 (t, *J* = 7.5 Hz, 2H); ^13^C NMR (126 MHz, DMSO-*d*_6_) δ 162.4 (s), 160.4 (s), 155.5 (s), 150.9 (s), 145.6 (s), 138.9 (s), 136.7 (s), 132.4 (s), 130.2 (s), 129.1 (s), 128.5 (s), 127.6 (s), 123.2 (s), 121.4 (s), 118.7 (s), 111.9 (s), 111.9 (s), 25.8 (s); HRMS, *m*/*z* calcd. for C_20_H_18_N_5_O_2_S_2_^+^ [M+H]^+^ 424.0896, found 424.0894.

*N*-(2-(1*H*-indol-3-yl)ethyl)-2-(2-(furan-2-ylmethylene)hydrazine-1-carbonyl)thiazole-4-carboxamide (**BAD-3**): Light brown solid; yield, 80.1%; M.p. 146–148 °C; ^1^H NMR (500 MHz, DMSO-*d*_6_) δ 12.07 (s, 1H), 10.84 (s, 1H), 8.59 (s, 1H), 8.52–8.46 (m, 2H), 7.91 (d, *J* = 1.3 Hz, 1H), 7.61 (d, *J* = 7.8 Hz, 1H), 7.35 (d, *J* = 8.1 Hz, 1H), 7.22 (d, *J* = 2.0 Hz, 1H), 7.07 (t, *J* = 7.2 Hz, 1H), 7.03 (d, *J* = 3.4 Hz, 1H), 6.99 (t, *J* = 7.4 Hz, 1H), 6.68 (dd, *J* = 3.4, 1.7 Hz, 1H), 3.66–3.62 (m, 2H), 3.01 (t, *J* = 7.5 Hz, 2H); ^13^C NMR (126 MHz, DMSO-*d*_6_) δ 162.4 (s), 160.4 (s), 155.6 (s), 150.9 (s), 149.4 (s), 146.2 (s), 140.1 (s), 136.7 (s), 129.2 (s), 127.6 (s), 123.1 (s), 121.4 (s), 118.7 (s), 115.2 (s), 112.8 (s), 111.9 (s), 111.8 (s), 25.8 (s); HRMS, *m*/*z* calcd. for C_20_H_18_N_5_O_3_S^+^ [M+H]^+^ 408.1125, found 408.1123.

*N*-(2-(1*H*-indol-3-yl)ethyl)-2-(2-(thiazol-2-ylmethylene)hydrazine-1-cabonyl)thiazole-4-carboxamide (**BAD-4**): Yellow solid; yield, 76.7%; M.p. 206–208 °C; ^1^H NMR (500 MHz, DMSO-*d*_6_) δ 14.71 (s, 1H), 10.81 (s, 1H), 8.66 (s, 1H), 8.18 (t, *J* = 5.7 Hz, 1H), 8.11 (s, 1H), 8.01 (d, *J* = 3.1 Hz, 1H), 7.82 (d, *J* = 3.0 Hz, 1H), 7.64 (d, *J* = 7.8 Hz, 1H), 7.32 (d, *J* = 8.1 Hz, 1H), 7.22 (d, *J* = 2.0 Hz, 1H), 7.11–7.05 (m, 1H), 7.01–6.94 (m, 1H), 3.73–3.69 (m, 2H), 3.05 (t, *J* = 7.1 Hz, 2H); ^13^C NMR (126 MHz, DMSO-*d*_6_) δ 162.4 (s), 160.3 (s), 159.7 (s), 156.6 (s), 151.4 (s), 144.5 (s), 136.8 (s), 134.2 (s), 130.2 (s), 127.6 (s), 124.8 (s), 123.1 (s), 121.4 (s), 118.8 (s), 118.7 (s), 112.0 (s), 111.9 (s), 25.7 (s); HRMS, *m*/*z* calcd. for C_19_H_17_N_6_O_2_S_2_^+^ [M+H]^+^ 425.0849, found 425.0847.

*N*-(2-(1*H*-indol-3-yl)ethyl)-2-(2-(pyridin-2-ylmethylene)hydrazine-1-carbonyl)thiazole-4-carboxamide (**BAD-5**): White solid; yield, 89.9%; M.p. 216–218 °C; ^1^H NMR (500 MHz, DMSO-*d*_6_) δ 12.31 (s, 1H), 10.85 (s, 1H), 8.69–8.59 (m, 3H), 8.55 (t, *J* = 6.0 Hz, 1H), 8.02 (d, *J* = 7.9 Hz, 1H), 7.92 (td, *J* = 7.7, 1.5 Hz, 1H), 7.62 (d, *J* = 7.9 Hz, 1H), 7.47 (ddd, *J* = 7.4, 4.9, 1.1 Hz, 1H), 7.35 (d, *J* = 8.1 Hz, 1H), 7.23 (d, *J* = 2.1 Hz, 1H), 7.11–7.05 (m, 1H), 7.03–6.96 (m, 1H), 3.68–3.63 (m, 2H), 3.02 (t, *J* = 7.5 Hz, 2H). ^13^C NMR (126 MHz, DMSO-*d*_6_) δ 162.1 (s), 160.4 (s), 155.9 (s), 153.2 (s), 151.0 (s), 150.6 (s), 150.1 (s), 137.4 (s), 136.7 (s), 129.4 (s), 127.6 (s), 125.3 (s), 123.2 (s), 121.4 (s), 120.7 (s), 118.7 (s), 112.0 (s), 111.8 (s), 25.8 (s); HRMS, *m*/*z* calcd. for C_21_H_19_N_6_O_2_S^+^ [M+H]^+^ 419.1285, found 419.1282.

*N*-(2-(1*H*-indol-3-yl)ethyl)-2-(2-((6-chloropyridin-2-yl)methylene)hydrazine-1-carbonyl)thiazole-4-carboxamide (**BAD-6**): White solid; yield, 80.5%; M.p. 220–222 °C; ^1^H NMR (500 MHz, DMSO-*d*_6_) δ 12.43 (s, 1H), 10.85 (s, 1H), 8.63 (s, 1H), 8.57–8.49 (m, 2H), 8.01–7.95 (m, 2H), 7.62–7.58 (m, 2H), 7.35 (d, *J* = 8.1 Hz, 1H), 7.23 (d, *J* = 2.0 Hz, 1H), 7.08 (t, *J* = 7.2 Hz, 1H), 6.99 (t, *J* = 7.3 Hz, 1H), 3.67–3.63 (m, 2H), 3.02 (t, *J* = 7.5 Hz, 2H). ^13^C NMR (126 MHz, DMSO-*d*_6_) δ 162.0 (s), 160.4 (s), 156.1 (s), 153.9 (s), 151.1 (s), 150.6 (s), 148.7 (s), 141.0 (s), 136.7 (s), 129.6 (s), 127.6 (s), 125.7 (s), 123.2 (s), 121.4 (s), 120.0 (s), 118.7 (s), 112.0 (s), 111.8 (s), 25.8 (s); HRMS, *m*/*z* calcd. for C_21_H_18_ClN_6_O_2_S^+^ [M+H]^+^ 453.0895, found 453.0895.

*N*-(2-(1*H*-indol-3-yl)ethyl)-2-(2-(pyridin-4-ylmethylene)hydrazine-1-carbonyl)thiazole-4-carboxamide (**BAD-7**): Yellow solid; yield, 87.3%; M.p. 142–145 °C; ^1^H NMR (500 MHz, DMSO-*d*_6_) δ 12.32 (s, 1H), 10.84 (s, 1H), 8.70 (d, *J* = 5.7 Hz, 2H), 8.62 (s, 1H), 8.59 (s, 1H), 8.53 (t, *J* = 5.9 Hz, 1H), 7.71 (d, *J* = 6.0 Hz, 2H), 7.61 (d, *J* = 7.8 Hz, 1H), 7.35 (d, *J* = 8.1 Hz, 1H), 7.22 (d, *J* = 2.0 Hz, 1H), 7.10–7.04 (m, 1H), 7.01–6.98 (m, 1H), 3.67–3.63 (m, 2H), 3.02 (t, *J* = 7.5 Hz, 2H). ^13^C NMR (126 MHz, DMSO-*d*_6_) δ 162.0 (s), 160.4 (s), 156.0 (s), 151.0 (s), 150.8 (s), 148.2 (s), 141.4 (s), 136.7 (s), 129.5 (s), 127.6 (s), 123.2 (s), 121.6 (s), 121.4 (s), 118.7 (s), 118.7 (s), 111.9 (s), 111.9 (s), 25.8 (s); HRMS, *m*/*z* calcd. for C_21_H_19_N_6_O_2_S^+^ [M+H]^+^ 419.1285, found 419.1281.

*N*-(2-(1*H*-indol-3-yl)ethyl)-2-(2-(naphthalen-2-ylmethylene)hydrazine-1-carbonyl)thiazole-4-carboxamide (**BAD-8**): White solid; yield, 93.6%; M.p. 238–241 °C; ^1^H NMR (500 MHz, DMSO-*d*_6_) δ 12.17 (s, 1H), 10.86 (s, 1H), 8.75 (s, 1H), 8.61 (s, 1H), 8.57 (t, *J* = 6.0 Hz, 1H), 8.21 (s, 1H), 8.08–8.02 (m, 1H), 8.01 (d, *J* = 0.7 Hz, 2H), 8.00–7.94 (m, 1H), 7.66–7.57 (m, 3H), 7.36 (d, *J* = 8.1 Hz, 1H), 7.24 (d, *J* = 2.2 Hz, 1H), 7.12–7.06 (m, 1H), 7.03–6.97 (m, 1H), 3.69–3.65 (m, 2H), 3.04 (t, *J* = 7.5 Hz, 2H). ^13^C NMR (126 MHz, DMSO-*d*_6_) δ 162.4 (s), 160.5 (s), 155.7 (s), 150.9 (s), 150.5 (s), 136.7 (s), 134.4 (s), 133.2 (s), 132.0 (s), 129.8 (s), 129.2 (s), 129.1 (s), 128.9 (s), 128.3 (s), 127.9 (s), 127.6 (s), 127.3 (s), 123.2 (s), 123.1 (s), 121.4 (s), 118.7 (s), 112.0 (s), 111.9 (s), 25.8 (s); HRMS, *m*/*z* calcd. for C_26_H_22_N_5_O_2_S^+^ [M+H]^+^ 468.1489, found 468.1487.

*N*-(2-(1*H*-indol-3-yl)ethyl)-2-(2-((1*H*-pyrrol-2-yl)methylene)hydrazine-1-carbonyl)thiazole-4-carboxamide (**BAD-9**): Yellow solid; yield, 67.7%; M.p. 228–231 °C; ^1^H NMR (500 MHz, DMSO-*d*_6_) δ 11.89 (s, 1H), 11.65 (s, 1H), 10.84 (s, 1H), 8.67 (t, *J* = 5.9 Hz, 1H), 8.55 (s, 1H), 8.47 (s, 1H), 7.61 (d, *J* = 7.8 Hz, 1H), 7.35 (d, *J* = 8.1 Hz, 1H), 7.22 (d, *J* = 1.7 Hz, 1H), 7.07 (t, *J* = 7.3 Hz, 1H), 7.02–6.94 (m, 2H), 6.56 (s, 1H), 6.18 (dd, *J* = 5.3, 2.3 Hz, 1H), 3.65–3.61 (m, 2H), 3.02 (t, *J* = 7.5 Hz, 2H). ^13^C NMR (126 MHz, DMSO-*d*_6_) δ 162.8 (s), 160.5 (s), 155.1 (s), 150.8 (s), 143.3 (s), 136.7 (s), 128.7 (s), 127.6 (s), 127.0 (s), 123.8 (s), 123.1 (s), 121.4 (s), 118.7 (s), 115.0 (s), 112.0 (s), 111.8 (s), 110.0 (s), 25.8 (s); HRMS, *m*/*z* calcd. for C_20_H_19_N_6_O_2_S^+^ [M+H]^+^ 407.1285, found 407.1282.

*N*-(2-(1*H*-indol-3-yl)ethyl)-2-(2-(quinolin-2-ylmethylene)hydrazine-1-carbonyl)thiazole-4-carboxamide (**BAD-10**): Yellow solid; yield, 82.7%; M.p. 228–230 °C; ^1^H NMR (500 MHz, DMSO-*d*_6_) δ 12.48 (s, 1H), 10.86 (s, 1H), 8.79 (s, 1H), 8.64 (s, 1H), 8.57 (t, *J* = 6.0 Hz, 1H), 8.47 (d, *J* = 8.6 Hz, 1H), 8.16 (d, *J* = 8.6 Hz, 1H), 8.08 (d, *J* = 8.4 Hz, 1H), 8.04 (d, *J* = 7.9 Hz, 1H), 7.86–7.79 (m, 1H), 7.69–7.65 (m, 1H), 7.63 (d, *J* = 7.8 Hz, 1H), 7.36 (d, *J* = 8.1 Hz, 1H), 7.24 (d, *J* = 1.9 Hz, 1H), 7.09 (t, *J* = 7.2 Hz, 1H), 7.00 (t, *J* = 7.4 Hz, 1H), 3.69–3.65 (m, 2H), 3.04 (t, *J* = 7.5 Hz, 2H); ^13^C NMR (126 MHz, DMSO-*d*_6_) δ 162.1 (s), 160.4 (s), 156.1 (s), 153.8 (s), 151.1 (s), 150.6 (s), 147.8 (s), 137.4 (s), 136.7 (s), 130.6 (s), 129.5 (s), 129.5 (s), 128.5 (s), 128.0 (s), 127.6 (s), 123.2 (s), 121.4 (s), 118.7 (s), 118.0 (s), 112.0 (s), 111.9 (s), 25.8 (s); HRMS, *m*/*z* calcd. for C_25_H_21_N_6_O_2_S^+^ [M+H]^+^ 469.1441, found 469.1441.

*N*-(2-(1*H*-indol-3-yl)ethyl)-2-(2-(2-(trifluoromethyl)benzylidene)hydrazine-1-carbonyl)thiazole-4-carboxamide (**BAD-11**): Yellow solid; yield, 79.6%; M.p. 246–248 °C; ^1^H NMR (500 MHz, DMSO-*d*_6_) δ 12.53 (s, 1H), 10.83 (s, 1H), 9.00 (d, *J* = 1.8 Hz, 1H), 8.62 (s, 1H), 8.49 (t, *J* = 6.0 Hz, 1H), 8.25 (d, *J* = 7.9 Hz, 1H), 7.84 (d, *J* = 7.8 Hz, 1H), 7.81 (t, *J* = 7.6 Hz, 1H), 7.69 (t, *J* = 7.6 Hz, 1H), 7.62 (d, *J* = 7.8 Hz, 1H), 7.35 (d, *J* = 8.1 Hz, 1H), 7.22 (d, *J* = 2.2 Hz, 1H), 7.10–7.05 (m, 1H), 7.02–6.96 (m, 1H), 3.67–3.63 (m, 2H), 3.01 (t, *J* = 7.5 Hz, 2H); ^13^C NMR (126 MHz, DMSO-*d*_6_) δ 162.4 (s), 160.5 (s), 156.2 (s), 151.2 (s), 145.5 (s), 136.7 (s), 133.4 (s), 132.2 (s), 131.0 (s), 129.5 (s), 127.8 (q, *J* = 8.3 Hz), 127.6 (s), 127.6 (s), 127.4 (s), 126.5 (q, *J* = 5.7 Hz), 124.5 (d, *J* = 273.9 Hz), 123.1 (s), 121.4 (s), 112.0 (s), 111.8 (s), 25.7 (s); HRMS, *m*/*z* calcd. for C_23_H_19_F_3_N_5_O_2_S^+^ [M+H]^+^ 486.1206, found 486.1205.

*N*-(2-(1*H*-indol-3-yl)ethyl)-2-(2-(3-(trifluoromethyl)benzylidene)hydrazine-1-carbonyl)thiazole-4-carboxamide (**BAD-12**): White solid; yield, 56.9%; M.p. 221–223 °C; ^1^H NMR (500 MHz, DMSO-*d*_6_) δ 12.26 (s, 1H), 10.84 (s, 1H), 8.68 (s, 1H), 8.61 (s, 1H), 8.54 (t, *J* = 5.9 Hz, 1H), 8.13–8.04 (m, 2H), 7.85 (d, *J* = 7.8 Hz, 1H), 7.74 (t, *J* = 7.8 Hz, 1H), 7.61 (d, *J* = 7.9 Hz, 1H), 7.36 (d, *J* = 8.1 Hz, 1H), 7.23 (d, *J* = 1.9 Hz, 1H), 7.08 (t, *J* = 7.5 Hz, 1H), 7.00 (t, *J* = 7.4 Hz, 1H), 3.68–3.64 (m, 2H), 3.02 (t, *J* = 7.5 Hz, 2H); ^13^C NMR (126 MHz, DMSO-*d*_6_) δ 162.1 (s), 160.4 (s), 155.9 (s), 150.9 (s), 148.8 (s), 136.7 (s), 135.4 (s), 131.8 (s), 130.6 (s), 130.2 (q, *J* = 31.8 Hz), 129.3 (s), 127.6 (s), 127.3 (q, *J* = 3.4 Hz), 124.4 (d, *J* = 272.4 Hz), 123.7 (q, *J* = 3.5 Hz), 123.2 (s), 121.4 (s), 118.7 (s), 118.7 (s), 111.9 (s), 111.9 (s), 25.8 (s); HRMS, *m*/*z* calcd. for C_23_H_19_F_3_N_5_O_2_S^+^ [M+H]^+^ 486.1206, found 486.1205.

*N*-(2-(1*H*-indol-3-yl)ethyl)-2-(2-(4-(trifluoromethyl)benzylidene)hydrazine-1-carbonyl)thiazole-4-carboxamide (**BAD-13**): White solid; yield, 89.1%; M.p. 239–242 °C; ^1^H NMR (500 MHz, DMSO-*d*_6_) δ 12.25 (s, 1H), 10.85 (s, 1H), 8.67 (s, 1H), 8.62 (s, 1H), 8.54 (t, *J* = 6.0 Hz, 1H), 7.99 (d, *J* = 8.1 Hz, 2H), 7.85 (d, *J* = 8.3 Hz, 2H), 7.61 (d, *J* = 7.9 Hz, 1H), 7.35 (d, *J* = 8.1 Hz, 1H), 7.23 (d, *J* = 2.0 Hz, 1H), 7.08 (t, *J* = 7.3 Hz, 1H), 6.99 (t, *J* = 7.4 Hz, 1H), 3.68–3.63 (m, 2H), 3.02 (t, *J* = 7.5 Hz, 2H); ^13^C NMR (126 MHz, DMSO-*d*_6_) δ 162.2 (s), 162.1 (s), 160.4 (s), 155.9 (s), 155.8 (s), 150.9 (s), 150.9 (s), 148.9 (s), 148.8 (s), 138.2 (s), 136.7 (s), 136.5 (s), 134.2 (s), 131.3 (s), 130.7 (s), 130.7 (s), 129.4 (s), 129.3 (s), 128.4 (s), 127.6 (s), 127.0 (s), 126.5 (s), 126.3 (q, *J* = 4.1 Hz), 124.5 (d, *J* = 272.0 Hz), 123.2 (s), 121.4 (s), 118.7 (s), 118.7 (s), 111.9 (s), 111.9 (s), 25.8 (s); HRMS, *m*/*z* calcd. for C_23_H_19_F_3_N_5_O_2_S^+^ [M+H]^+^ 486.1206, found 486.1206.

*N*-(2-(1*H*-indol-3-yl)ethyl)-2-(2-(2-fluorobenzylidene)hydrazine-1-carbonyl)thiazole-4-carboxamide (**BAD-14**): White solid, yield 79.5%, M.p. 232–235 °C; ^1^H NMR (500 MHz, DMSO-*d*_6_) δ 12.21 (s, 1H), 10.84 (s, 1H), 8.83 (s, 1H), 8.61 (s, 1H), 8.53 (t, *J* = 5.9 Hz, 1H), 7.98 (td, *J* = 7.6, 1.4 Hz, 1H), 7.61 (d, *J* = 7.9 Hz, 1H), 7.57–7.50 (m, 1H), 7.40–7.29 (m, 3H), 7.22 (d, *J* = 2.1 Hz, 1H), 7.11–7.04 (m, 1H), 7.02–6.95 (m, 1H), 3.67–3.63 (m, 2H), 3.02 (t, *J* = 7.5 Hz, 2H); ^13^C NMR (126 MHz, DMSO-*d*_6_) δ 162.4 (s), 161.3 (d, *J* = 221.9 Hz), 160.4 (s), 155.7 (s), 150.9 (s), 143.2 (d, *J* = 4.7 Hz), 136.7 (s), 133.0 (d, *J* = 8.6 Hz), 129.3 (s), 127.6 (s), 127.0 (s), 125.5 (d, *J* = 3.0 Hz), 123.2 (s), 121.8 (d, *J* = 9.9 Hz), 121.4 (s), 118.7 (s), 116.5 (d, *J* = 20.6 Hz), 111.9 (s), 111.89 (s), 25.8 (s); HRMS, *m*/*z* calcd. for C_22_H_19_FN_5_O_2_S^+^ [M+H]^+^ 436.1238, found 436.1237.

*N*-(2-(1*H*-indol-3-yl)ethyl)-2-(2-(3-fluorobenzylidene)hydrazine-1-carbonyl)thiazole-4-carboxamide (**BAD-15**): Light yellow solid, yield 62.1%, M.p. 209–212 °C; ^1^H NMR (500 MHz, DMSO-*d*_6_) δ 12.17 (s, 1H), 10.85 (s, 1H), 8.61 (s, 1H), 8.60 (s, 1H), 8.55 (t, *J* = 6.0 Hz, 1H), 7.64–7.60 (m, 2H), 7.59–7.50 (m, 2H), 7.38–7.30 (m, 2H), 7.22 (d, *J* = 1.7 Hz, 1H), 7.08 (t, *J* = 7.5 Hz, 1H), 6.99 (t, *J* = 7.4 Hz, 1H), 3.67–3.63 (m, 2H), 3.02 (t, *J* = 7.5 Hz, 2H); ^13^C NMR (126 MHz, DMSO-*d*_6_) δ 163.8 (s), 161.9 (s), 161.3 (d, *J* = 223.2 Hz), 155.8 (s), 150.9 (s), 149.2 (d, *J* = 2.7 Hz), 136.8 (d, *J* = 7.8 Hz), 136.7 (s), 131.5 (d, *J* = 8.3 Hz), 129.3 (s), 127.6 (s), 124.2 (d, *J* = 2.4 Hz), 123.2 (s), 121.4 (s), 117.8 (d, *J* = 21.2 Hz), 113.7 (d, *J* = 22.8 Hz), 111.9 (d, *J* = 9.9 Hz), 25.8 (s); HRMS, *m*/*z* calcd. for C_22_H_19_FN_5_O_2_S^+^ [M+H]^+^ 436.1238, found 436.1239.

*N*-(2-(1*H*-indol-3-yl)ethyl)-2-(2-(4-fluorobenzylidene)hydrazine-1-carbonyl)thiazole-4-carboxamide (**BAD-16**): Yellow solid, yield 82.3%, M.p. 128–129 °C; ^1^H NMR (500 MHz, DMSO-*d*_6_) δ 12.07 (s, 1H), 10.84 (s, 1H), 8.60 (s, 1H), 8.59 (s, 1H), 8.54 (t, *J* = 6.0 Hz, 1H), 7.86–7.79 (m, 2H), 7.61 (d, *J* = 7.9 Hz, 1H), 7.38–7.30 (m, 3H), 7.22 (d, *J* = 2.1 Hz, 1H), 7.11–7.04 (m, 1H), 7.02–6.95 (m, 1H), 3.67–3.63 (m, 2H), 3.02 (t, *J* = 7.5 Hz, 2H); ^13^C NMR (126 MHz, DMSO-*d*_6_) δ 163.91 (d, *J* = 248.4 Hz), 162.38 (s), 162.16 (s), 160.46 (d, *J* = 1.4 Hz), 155.95 (s), 155.71 (s), 150.95 (d, *J* = 7.8 Hz), 149.48 (s), 148.83 (s), 138.23 (s), 136.75 (s), 130.90 (d, J = 2.8 Hz), 130.76 (s), 130.11 (d, *J* = 8.7 Hz), 129.33 (d, *J* = 25.0 Hz), 128.45 (s), 127.66 (s), 126.31 (d, J = 3.6 Hz), 123.20 (s), 121.48 (s), 118.77 (s), 116.54 (d, *J* = 22.0 Hz), 111.94 (d, *J* = 10.1 Hz), 25.84 (s); HRMS, *m*/*z* calcd. for C_22_H_19_FN_5_O_2_S^+^ [M+H]^+^ 436.1238, found 436.1235.

*N*-(2-(1*H*-indol-3-yl)ethyl)-2-(2-(2-chlorobenzylidene)hydrazine-1-carbonyl)thiazole-4-carboxamide (**BAD-17**): White solid, yield 61.4%, M.p. 244–247 °C; ^1^H NMR (500 MHz, DMSO-*d*_6_) δ 12.34 (s, 1H), 10.83 (s, 1H), 8.99 (s, 1H), 8.61 (s, 1H), 8.51 (t, *J* = 6.0 Hz, 1H), 8.06 (dd, *J* = 7.6, 1.8 Hz, 1H), 7.61 (d, *J* = 7.9 Hz, 1H), 7.57 (dd, *J* = 7.8, 1.2 Hz, 1H), 7.53–7.44 (m, 2H), 7.35 (d, *J* = 8.1 Hz, 1H), 7.22 (d, *J* = 1.9 Hz, 1H), 7.07 (t, *J* = 7.5 Hz, 1H), 6.99 (t, *J* = 7.4 Hz, 1H), 3.67–3.63 (m, 2H), 3.02 (t, *J* = 7.5 Hz, 2H); ^13^C NMR (126 MHz, DMSO-*d*_6_) δ 162.2 (s), 160.4 (s), 155.9 (s), 151.0 (s), 146.3 (s), 136.7 (s), 134.0 (s), 132.5 (s), 131.6 (s), 130.5 (s), 129.4 (s), 128.2 (s), 127.6 (s), 127.5 (s), 123.2 (s), 121.4 (s), 118.7 (s), 111.9 (s), 111.8 (s), 25.8 (s); HRMS, *m*/*z* calcd. for C_22_H_19_ClN_5_O_2_S^+^ [M+H]^+^ 452.0942, found 452.0942.

*N*-(2-(1*H*-indol-3-yl)ethyl)-2-(2-(3-chlorobenzylidene)hydrazine-1-carbonyl)thiazole-4-carboxamide (**BAD-18**): White solid, yield 68.3%, M.p. 191–192 °C; ^1^H NMR (500 MHz, DMSO-*d*_6_) δ 12.19 (s, 1H), 10.84 (s, 1H), 8.61 (s, 1H), 8.57 (s, 1H), 8.54 (t, *J* = 6.0 Hz, 1H), 7.81 (s, 1H), 7.77–7.70 (m, 1H), 7.61 (d, *J* = 7.8 Hz, 1H), 7.57–7.49 (m, 2H), 7.35 (d, *J* = 8.1 Hz, 1H), 7.22 (d, *J* = 2.0 Hz, 1H), 7.10–7.05 (m, 1H), 6.99 (t, *J* = 7.1 Hz, 1H), 3.67–3.63 (m, 2H), 3.02 (t, *J* = 7.5 Hz, 2H); ^13^C NMR (126 MHz, DMSO-*d*_6_) δ 164.9 (s), 162.9 (s), 162.3 (s), 162.2 (s), 160.4 (s), 155.8 (s), 155.7 (s), 150.9 (s), 150.9 (s), 149.4 (s), 148.9 (s), 136.7 (s), 136.5 (s), 134.2 (s), 131.3 (s), 130.9 (s), 130.8 (s), 130.7 (s), 130.1 (s), 130.0 (s), 129.3 (s), 129.2 (s), 127.6 (s), 127.0 (s), 126.5 (s), 123.2 (s), 121.4 (s), 118.7 (s), 118.7 (s), 116.6 (s), 116.4 (s), 111.9 (s), 111.9 (s), 25.8 (s); HRMS, *m*/*z* calcd. for C_22_H_19_ClN_5_O_2_S^+^ [M+H]^+^ 452.0942, found 452.0944.

*N*-(2-(1*H*-indol-3-yl)ethyl)-2-(2-(4-chlorobenzylidene)hydrazine-1-carbonyl)thiazole-4-carboxamide (**BAD-19**): White solid, yield 59.0%, M.p. 244–246 °C; ^1^H NMR (500 MHz, DMSO-*d*_6_) δ 12.12 (s, 1H), 10.84 (s, 1H), 8.60 (s, 1H), 8.58 (s, 1H), 8.53 (t, *J* = 6.0 Hz, 1H), 7.79 (d, *J* = 8.5 Hz, 2H), 7.61 (d, *J* = 7.8 Hz, 1H), 7.56 (d, *J* = 8.5 Hz, 2H), 7.35 (d, *J* = 8.1 Hz, 1H), 7.22 (d, *J* = 2.0 Hz, 1H), 7.08 (t, *J* = 7.2 Hz, 1H), 6.99 (t, *J* = 7.4 Hz, 1H), 3.67–3.63 (m, 2H), 3.02 (t, *J* = 7.5 Hz, 2H); ^13^C NMR (126 MHz, DMSO-*d*_6_) δ 162.3 (s), 160.4 (s), 155.7 (s), 150.9 (s), 149.2 (s), 136.7 (s), 135.5 (s), 133.2 (s), 129.5 (s), 129.4 (s), 129.3 (s), 127.6 (s), 123.2 (s), 121.4 (s), 118.7 (s), 118.7 (s), 111.9 (s), 111.9 (s), 25.8 (s); HRMS, *m*/*z* calcd. for C_22_H_19_ClN_5_O_2_S^+^ [M+H]^+^ 452.0942, found 452.0942.

*N*-(2-(1*H*-indol-3-yl)ethyl)-2-(2-(2-bromobenzylidene)hydrazine-1-carbonyl)thiazole-4-carboxamide (**BAD-20**): White solid, yield 48.5%, M.p. 244–246 °C; ^1^H NMR (500 MHz, DMSO-*d*_6_) δ 12.40 (s, 1H), 10.84 (s, 1H), 8.94 (s, 1H), 8.61 (s, 1H), 8.52 (t, *J* = 6.0 Hz, 1H), 8.04 (dd, *J* = 7.8, 1.5 Hz, 1H), 7.73 (d, *J* = 7.9 Hz, 1H), 7.61 (d, *J* = 7.9 Hz, 1H), 7.50 (t, *J* = 7.5 Hz, 1H), 7.42 (td, *J* = 7.7, 1.6 Hz, 1H), 7.35 (d, *J* = 8.1 Hz, 1H), 7.22 (d, *J* = 2.0 Hz, 1H), 7.07 (t, *J* = 7.5 Hz, 1H), 6.99 (t, *J* = 7.4 Hz, 1H), 3.67–3.63 (m, 2H), 3.02 (t, *J* = 7.5 Hz, 2H); ^13^C NMR (126 MHz, DMSO-*d*_6_) δ 162.3 (s), 160.4 (s), 156.0 (s), 151.0 (s), 148.7 (s), 136.7 (s), 133.7 (s), 133.2 (s), 132.7 (s), 129.4 (s), 128.6 (s), 127.9 (s), 127.6 (s), 124.3 (s), 123.2 (s), 121.4 (s), 118.7 (s), 118.7 (s), 112.0 (s), 111.8 (s), 25.8 (s); HRMS, *m*/*z* calcd. for C_22_H_19_BrN_5_O_2_S^+^ [M+H]^+^ 496.0437, found 496.0441.

*N*-(2-(1*H*-indol-3-yl)ethyl)-2-(2-(3-bromobenzylidene)hydrazine-1-carbonyl)thiazole-4-carboxamide (**BAD-21**): White solid, yield 62.4%, M.p. 174–177 °C; ^1^H NMR (500 MHz, DMSO-*d*_6_) δ 12.19 (s, 1H), 10.84 (s, 1H), 8.61 (s, 1H), 8.57–8.50 (m, 2H), 7.95 (s, 1H), 7.77 (d, *J* = 7.8 Hz, 1H), 7.71–7.65 (m, 1H), 7.61 (d, *J* = 7.8 Hz, 1H), 7.46 (t, *J* = 7.9 Hz, 1H), 7.35 (d, *J* = 8.1 Hz, 1H), 7.22 (d, *J* = 2.1 Hz, 1H), 7.10–7.05 (m, 1H), 7.02–6.97 (m, 1H), 3.67–3.63 (m, 2H), 3.02 (t, *J* = 7.5 Hz, 2H); ^13^C NMR (126 MHz, DMSO-*d*_6_) δ 162.2 (s), 160.4 (s), 155.8 (s), 150.9 (s), 148.8 (s), 136.7 (s), 133.6 (s), 131.6 (s), 129.9 (s), 129.3 (s), 127.6 (s), 126.9 (s), 123.2 (s), 122.7 (s), 121.4 (s), 118.7 (s), 118.7 (s), 111.9 (s), 111.9 (s), 25.8 (s); HRMS, *m*/*z* calcd. for C_22_H_19_BrN_5_O_2_S^+^ [M+H]^+^ 496.0437, found 496.0437.

*N*-(2-(1*H*-indol-3-yl)ethyl)-2-(2-(4-bromobenzylidene)hydrazine-1-carbonyl)thiazole-4-carboxamide (**BAD-22**): White solid, yield 91.5%, M.p. 249–251 °C; ^1^H NMR (500 MHz, DMSO-*d*_6_) δ 12.13 (s, 1H), 10.85 (s, 1H), 8.60 (s, 1H), 8.57–8.50 (m, 2H), 7.76–7.67 (m, 4H), 7.61 (d, *J* = 7.9 Hz, 1H), 7.35 (d, *J* = 8.1 Hz, 1H), 7.22 (d, *J* = 2.0 Hz, 1H), 7.07 (dd, *J* = 11.1, 3.9 Hz, 1H), 6.99 (dd, *J* = 11.0, 3.9 Hz, 1H), 3.67–3.63 (m, 2H), 3.02 (t, *J* = 7.5 Hz, 2H); ^13^C NMR (126 MHz, DMSO-*d*_6_) δ 162.3 (s), 160.4 (s), 155.7 (s), 150.9 (s), 149.3 (s), 136.7 (s), 133.5 (s), 132.4 (s), 129.6 (s), 129.3 (s), 127.6 (s), 124.4 (s), 123.2 (s), 121.4 (s), 118.7 (s), 118.7 (s), 111.9 (s), 111.9 (s), 25.8 (s); HRMS, *m*/*z* calcd. for C_22_H_19_BrN_5_O_2_S^+^ [M+H]^+^ 496.0437, found 496.0436.

*N*-(2-(1*H*-indol-3-yl)ethyl)-2-(2-(2-methoxybenzylidene)hydrazine-1-carbonyl)thiazole-4-carboxamide (**BAD-23**): White solid, yield 65.5%, M.p. 244–247 °C; ^1^H NMR (500 MHz, DMSO-*d*_6_) δ 12.02 (s, 1H), 10.84 (s, 1H), 8.91 (s, 1H), 8.63–8.53 (m, 2H), 7.90 (dd, *J* = 7.7, 1.6 Hz, 1H), 7.62 (d, *J* = 7.8 Hz, 1H), 7.51–7.43 (m, 1H), 7.35 (d, *J* = 8.1 Hz, 1H), 7.22 (d, *J* = 2.1 Hz, 1H), 7.15 (d, *J* = 8.3 Hz, 1H), 7.09–7.04 (m, 2H), 7.01–6.96 (m, 1H), 3.89 (s, 3H), 3.68–3.63 (m, 2H), 3.02 (t, *J* = 7.5 Hz, 2H); ^13^C NMR (126 MHz, DMSO-*d*_6_) δ 162.3 (s), 160.4 (s), 158.4 (s), 155.5 (s), 150.7 (s), 145.7 (s), 136.7 (s), 132.6 (s), 129.1 (s), 127.6 (s), 126.2 (s), 123.2 (s), 122.2 (s), 121.4 (s), 121.3 (s), 118.7 (s), 112.4 (s), 111.9 (s), 111.8 (s), 56.1 (s), 25.9 (s); HRMS, *m*/*z* calcd. for C_23_H_22_N_5_O_3_S^+^ [M+H]^+^ 448.1438, found 448.1436.

*N*-(2-(1*H*-indol-3-yl)ethyl)-2-(2-(3-methoxybenzylidene)hydrazine-1-carbonyl)thiazole-4-carboxamide (**BAD-24**): Light yellow solid, yield 89.0%, M.p. 227–229 °C; ^1^H NMR (500 MHz, DMSO-*d*_6_) δ 12.06 (s, 1H), 10.85 (s, 1H), 8.60 (s, 1H), 8.58–8.52 (m, 2H), 7.61 (d, *J* = 7.9 Hz, 1H), 7.41 (t, *J* = 7.9 Hz, 1H), 7.37–7.30 (m, 3H), 7.22 (d, *J* = 2.1 Hz, 1H), 7.11–7.04 (m, 2H), 6.99 (dd, *J* = 10.9, 3.9 Hz, 1H), 3.82 (s, 3H), 3.67–3.63 (m, 2H), 3.02 (t, *J* = 7.5 Hz, 2H); ^13^C NMR (126 MHz, DMSO-*d*_6_) δ 162.3 (s), 160.4 (s), 160.0 (s), 155.7 (s), 150.9 (s), 150.5 (s), 136.7 (s), 135.6 (s), 130.5 (s), 129.2 (s), 127.6 (s), 123.2 (s), 121.4 (s), 120.8 (s), 118.7 (s), 117.2 (s), 111.9 (s), 111.9 (s), 55.7 (s), 25.8 (s); HRMS, *m*/*z* calcd. for C_23_H_22_N_5_O_3_S^+^ [M+H]^+^ 448.1438, found 448.1436.

*N*-(2-(1*H*-indol-3-yl)ethyl)-2-(2-(4-methoxybenzylidene)hydrazine-1-carbonyl)thiazole-4-carboxamide (**BAD-25**): Light yellow solid, yield 89.4%, M.p. 124–127 °C; ^1^H NMR (500 MHz, DMSO-*d*_6_) δ 11.92 (s, 1H), 10.84 (s, 1H), 8.58 (s, 1H), 8.55 (t, *J* = 6.0 Hz, 1H), 8.52 (s, 1H), 7.72 (d, *J* = 8.8 Hz, 2H), 7.61 (d, *J* = 7.8 Hz, 1H), 7.36 (d, *J* = 8.1 Hz, 1H), 7.22 (d, *J* = 2.0 Hz, 1H), 7.09–7.04 (m, 3H), 6.99 (t, *J* = 7.2 Hz, 1H), 3.82 (s, 3H), 3.67–3.63 (m, 2H), 3.02 (t, *J* = 7.5 Hz, 2H); ^13^C NMR (126 MHz, DMSO-*d*_6_) δ 162.6 (s), 161.7 (s), 160.4 (s), 155.4 (s), 150.8 (s), 150.5 (s), 136.7 (s), 129.5 (s), 129.0 (s), 127.6 (s), 126.7 (s), 123.2 (s), 121.4 (s), 118.7 (s), 114.9 (s), 111.9 (s), 111.9 (s), 55.8 (s), 25.8 (s); HRMS, *m*/*z* calcd. for C_23_H_22_N_5_O_3_S^+^ [M+H]^+^ 448.1438, found 448.1438.

*N*-(2-(1*H*-indol-3-yl)ethyl)-2-(2-(2-nitrobenzylidene)hydrazine-1-carbonyl)thiazole-4-carboxamide (**BAD-26**): Yellow solid, yield 80.7%, M.p. 226–229 °C; ^1^H NMR (500 MHz, DMSO-*d*_6_) δ 12.43 (s, 1H), 10.84 (s, 1H), 8.99 (s, 1H), 8.62 (s, 1H), 8.52 (t, *J* = 6.0 Hz, 1H), 8.17–8.08 (m, 2H), 7.86 (t, *J* = 7.6 Hz, 1H), 7.78–7.69 (m, 1H), 7.61 (d, *J* = 7.8 Hz, 1H), 7.35 (d, *J* = 8.1 Hz, 1H), 7.22 (d, *J* = 2.1 Hz, 1H), 7.11–7.05 (m, 1H), 7.02–6.95 (m, 1H), 3.67–3.63 (m, 2H), 3.02 (t, *J* = 7.5 Hz, 2H); ^13^C NMR (126 MHz, DMSO-*d*_6_) δ 162.1 (s), 160.4 (s), 156.1 (s), 151.0 (s), 148.8 (s), 145.8 (s), 136.7 (s), 134.3 (s), 131.6 (s), 129.5 (s), 128.8 (s), 128.7 (s), 127.6 (s), 125.2 (s), 123.2 (s), 121.4 (s), 118.7 (s), 111.9 (s), 111.8 (s), 25.8 (s); HRMS, *m*/*z* calcd. for C_22_H_19_N_6_O_4_S^+^ [M+H]^+^ 463.1183, found 463.1181.

*N*-(2-(1*H*-indol-3-yl)ethyl)-2-(2-(2-hydroxybenzylidene)hydrazine-1-carbonyl)thiazole-4-carboxamide (**BAD-27**): White solid, yield 90.7%, M.p. 224–226 °C; ^1^H NMR (500 MHz, DMSO-*d*_6_) δ 12.23 (s, 1H), 10.84 (s, 2H), 8.82 (s, 1H), 8.60 (s, 1H), 8.54 (t, *J* = 6.0 Hz, 1H), 7.67–7.59 (m, 2H), 7.39–7.30 (m, 2H), 7.22 (d, *J* = 1.9 Hz, 1H), 7.08 (t, *J* = 7.5 Hz, 1H), 7.01–6.92 (m, 3H), 3.68–3.63 (m, 2H), 3.02 (t, *J* = 7.5 Hz, 2H); ^13^C NMR (126 MHz, DMSO-*d*_6_) δ 162.1 (s), 160.4 (s), 157.8 (s), 155.5 (s), 150.9 (s), 149.5 (s), 136.7 (s), 132.4 (s), 129.2 (s), 129.0 (s), 127.6 (s), 123.2 (s), 121.4 (s), 120.0 (s), 119.3 (s), 118.7 (s), 116.9 (s), 111.9 (s), 111.9 (s), 25.8 (s); HRMS, *m*/*z* calcd. for C_22_H_20_N_5_O_3_S^+^ [M+H]^+^ 434.1281, found 434.1280.

*N*-(2-(1*H*-indol-3-yl)ethyl)-2-(2-(2,3-dichlorobenzylidene)hydrazine-1-carbonyl)thiazole-4-carboxamide (**BAD-28**): Light yellow solid, yield 95.5%, M.p. 262–264 °C; ^1^H NMR (500 MHz, DMSO-*d*_6_) δ 12.43 (s, 1H), 10.84 (s, 1H), 9.01 (s, 1H), 8.62 (s, 1H), 8.51 (t, *J* = 6.0 Hz, 1H), 8.02 (dd, *J* = 7.9, 1.4 Hz, 1H), 7.76 (dd, *J* = 8.0, 1.5 Hz, 1H), 7.62 (d, *J* = 7.8 Hz, 1H), 7.49 (t, *J* = 7.9 Hz, 1H), 7.35 (d, *J* = 8.1 Hz, 1H), 7.22 (d, *J* = 2.1 Hz, 1H), 7.11–7.05 (m, 1H), 7.03–6.94 (m, 1H), 3.68–3.63 (m, 2H), 3.02 (t, *J* = 7.5 Hz, 2H); ^13^C NMR (126 MHz, DMSO-*d*_6_) δ 162.1 (s), 160.4 (s), 156.0 (s), 151.0 (s), 146.1 (s), 136.7 (s), 134.1 (s), 132.9 (s), 132.4 (s), 131.8 (s), 129.5 (s), 129.0 (s), 127.6 (s), 126.1 (s), 123.2 (s), 121.4 (s), 118.7 (s), 112.0 (s), 111.8 (s), 25.8 (s); HRMS, *m*/*z* calcd. for C_22_H_18_Cl_2_N_5_O_2_S^+^ [M+H]^+^ 486.0553, found 486.0555.

*N*-(2-(1*H*-indol-3-yl)ethyl)-2-(2-(2,4-dichlorobenzylidene)hydrazine-1-carbonyl)thiazole-4-carboxamide (**BAD-29**): Light yellow solid, yield 69.9%, M.p. 131–133 °C; ^1^H NMR (500 MHz, DMSO-*d*_6_) δ 12.39 (s, 1H), 10.83 (s, 1H), 8.93 (s, 1H), 8.62 (s, 1H), 8.49 (t, *J* = 6.0 Hz, 1H), 8.05 (d, *J* = 8.6 Hz, 1H), 7.76 (d, *J* = 2.0 Hz, 1H), 7.61 (d, *J* = 7.9 Hz, 1H), 7.55 (dd, *J* = 8.5, 1.9 Hz, 1H), 7.35 (d, *J* = 8.1 Hz, 1H), 7.21 (d, *J* = 1.9 Hz, 1H), 7.07 (t, *J* = 7.2 Hz, 1H), 6.99 (t, *J* = 7.2 Hz, 1H), 3.67–3.63 (m, 2H), 3.01 (t, *J* = 7.5 Hz, 2H); ^13^C NMR (126 MHz, DMSO-*d*_6_) δ 162.1 (s), 160.4 (s), 155.9 (s), 151.0 (s), 145.3 (s), 136.7 (s), 136.1 (s), 134.7 (s), 130.8 (s), 129.9 (s), 129.4 (s), 128.7 (s), 128.6 (s), 127.6 (s), 123.2 (s), 121.4 (s), 118.7 (s), 112.0 (s), 111.8 (s), 25.8 (s); HRMS, *m*/*z* calcd. for C_22_H_18_Cl_2_N_5_O_2_S^+^ [M+H]^+^ 486.0553, found 486.0555.

*N*-(2-(1*H*-indol-3-yl)ethyl)-2-(2-(2,5-dichlorobenzylidene)hydrazine-1-carbonyl)thiazole-4-carboxamide (**BAD-30**): Light yellow solid, yield 89.7%, M.p. 138–140 °C; ^1^H NMR (500 MHz, DMSO-*d*_6_) δ 12.45 (s, 1H), 10.83 (s, 1H), 8.93 (s, 1H), 8.62 (s, 1H), 8.48 (t, *J* = 6.0 Hz, 1H), 7.98 (d, *J* = 2.6 Hz, 1H), 7.62–7.60 (m, 2H), 7.58–7.56 (m, 1H), 7.35 (d, *J* = 8.1 Hz, 1H), 7.21 (d, *J* = 2.1 Hz, 1H), 7.10–7.05 (m, 1H), 7.01–6.96 (m, 1H), 3.67–3.63 (m, 2H), 3.01 (t, *J* = 7.5 Hz, 2H); ^13^C NMR (126 MHz, DMSO-*d*_6_) δ 162.1 (s), 160.4 (s), 156.0 (s), 151.0 (s), 146.1 (s), 136.7 (s), 134.1 (s), 132.9 (s), 132.4 (s), 131.8 (s), 129.5 (s), 129.0 (s), 127.6 (s), 126.1 (s), 123.2 (s), 121.4 (s), 118.7 (s), 112.0 (s), 111.8 (s), 25.8 (s); HRMS, *m*/*z* calcd. for C_22_H_18_Cl_2_N_5_O_2_S^+^ [M+H]^+^ 486.0553, found 486.0555.

*N*-(2-(1*H*-indol-3-yl)ethyl)-2-(2-(3,4-dichlorobenzylidene)hydrazine-1-carbonyl)thiazole-4-carboxamide (**BAD-31**): White solid, yield 88.5%, M.p. 199–201 °C; ^1^H NMR (500 MHz, DMSO) δ ^1^H NMR (500 MHz, DMSO-*d*_6_) δ 12.25 (s, 1H), 10.84 (s, 1H), 8.61 (s, 1H), 8.56 (s, 1H), 8.52 (t, *J* = 5.9 Hz, 1H), 7.99 (d, *J* = 1.1 Hz, 1H), 7.79–7.72 (m, 2H), 7.61 (d, *J* = 7.8 Hz, 1H), 7.35 (d, *J* = 8.1 Hz, 1H), 7.22 (d, *J* = 1.9 Hz, 1H), 7.08 (t, *J* = 7.5 Hz, 1H), 6.99 (t, *J* = 7.4 Hz, 1H), 3.67–3.63 (m, 2H), 3.02 (t, *J* = 7.5 Hz, 2H); ^13^C NMR (126 MHz, DMSO-*d*_6_) δ 162.1 (s), 160.4 (s), 155.9 (s), 150.9 (s), 147.8 (s), 136.7 (s), 135.0 (s), 133.3 (s), 132.3 (s), 131.7 (s), 129.4 (s), 129.2 (s), 127.6 (s), 127.6 (s), 123.2 (s), 121.4 (s), 118.7 (s), 118.7 (s), 111.9 (s), 111.9 (s), 55.3 (s), 25.8 (s); HRMS, *m*/*z* calcd. for C_22_H_18_Cl_2_N_5_O_2_S^+^ [M+H]^+^ 486.0553, found 486.0553.

*N*-(2-(1*H*-indol-3-yl)ethyl)-2-(2-(3,5-dichlorobenzylidene)hydrazine-1-carbonyl)thiazole-4-carboxamide (**BAD-32**): White solid, yield 75.5%, M.p. 139–140 °C; ^1^H NMR (500 MHz, DMSO-*d*_6_) δ 12.25 (s, 1H), 10.84 (s, 1H), 8.61 (s, 1H), 8.56 (s, 1H), 8.52 (t, *J* = 5.9 Hz, 1H), 7.99 (d, *J* = 1.1 Hz, 1H), 7.80–7.73 (m, 2H), 7.61 (d, *J* = 7.8 Hz, 1H), 7.35 (d, *J* = 8.1 Hz, 1H), 7.22 (d, *J* = 1.9 Hz, 1H), 7.08 (t, *J* = 7.5 Hz, 1H), 6.99 (t, *J* = 7.4 Hz, 1H), 3.67–3.63 (m, 2H), 3.02 (t, *J* = 7.5 Hz, 2H); ^13^C NMR (126 MHz, DMSO-*d*_6_) δ 162.0 (s), 160.4 (s), 156.0 (s), 151.0 (s), 147.4 (s), 137.9 (s), 136.7 (s), 135.2 (s), 130.0 (s), 129.4 (s), 127.6 (s), 126.0 (s), 123.2 (s), 121.4 (s), 118.7 (s), 118.7 (s), 111.9 (s), 111.9 (s), 25.8 (s); HRMS, *m*/*z* calcd. for C_22_H_18_Cl_2_N_5_O_2_S^+^ [M+H]^+^ 486.0553, found 486.0554.

*N*-(2-(1*H*-indol-3-yl)ethyl)-2-(2-(2,6-dichlorobenzylidene)hydrazine-1-carbonyl)thiazole-4-carboxamide (**BAD-33**): Light yellow solid, yield 93.8%, M.p. 224–227 °C; ^1^H NMR (500 MHz, DMSO-*d*_6_) δ 12.42 (s, 1H), 10.84 (s, 1H), 8.80 (s, 1H), 8.62 (s, 1H), 8.54 (t, *J* = 6.0 Hz, 1H), 7.65–7.57 (m, 3H), 7.49 (dd, *J* = 8.5, 7.7 Hz, 1H), 7.35 (d, *J* = 8.1 Hz, 1H), 7.22 (d, *J* = 2.0 Hz, 1H), 7.08 (dd, *J* = 11.1, 4.0 Hz, 1H), 6.99 (t, *J* = 7.4 Hz, 1H), 3.67–3.63 (m, 2H), 3.01 (t, *J* = 7.5 Hz, 2H); ^13^C NMR (126 MHz, DMSO-*d*_6_) δ 162.1 (s), 160.4 (s), 156.0 (s), 151.0 (s), 145.9 (s), 136.7 (s), 134.4 (s), 132.0 (s), 130.8 (s), 129.6 (s), 129.5 (s), 127.6 (s), 123.1 (s), 121.4 (s), 118.7 (s), 112.0 (s), 111.8 (s), 25.8 (s); HRMS, *m*/*z* calcd. for C_22_H_18_Cl_2_N_5_O_2_S^+^ [M+H]^+^ 486.0553, found 486.0555.

### 2.3. Fungicidal Activity

The in *vitro* antifungal activities of the target compounds **BAD-1** to **BAD-33** were evaluated against *Alternaria solani*, *Botrytis cinerea*, *Fusarium graminearum*, *Physalospora piricola*, *Rhizoctonia solani*, *Fusarium verticillioides*, and *Fusdrzum oxysporum* using the method described in our previous study [36]. For comparative purposes, the commercially available fungicide thifluzamide was included in the evaluation. For the preparation of the stock solution, 5 mg of the test compound was accurately weighed and dissolved in 200 μL of *N, N*-dimethylformamide (DMF) to achieve a concentration of 25 mg/mL. To prepare the working solution at a test concentration of 500 μg/mL, 100 μL of the stock solution was accurately transferred to 4900 μL of sterile water containing 0.1% Tween 80 and thoroughly mixed. Finally, a 50 μg/mL potato dextrose agar (PDA) medium was prepared by uniformly mixing 4 mL of the working solution with 36 mL of molten PDA. The control was prepared by adding an equivalent volume of pure DMF to PDA medium according to the corresponding amount described above. Each experiment was conducted in triplicate. The relative inhibition (I, %) was calculated using the equation below:

I (%) = [(C − T)/(C − 4)] × 100%

where I represents the rate of fungal growth inhibition, C denotes the average diameter of the control colony (mm), and T indicates the average diameter of the mycelial colony in the experiment (mm). The diameter of the mycelial cake inoculated on PDA was 4 mm.

Compounds exhibiting higher inhibitory activity were selected for evaluation of their half-maximal effective concentration (EC_50_) values. The EC_50_ values of selected compounds were calculated by non-linear regression analysis using Prism 8 v. 8.30 (GraphPad Software, La Jolla, CA, USA).

### 2.4. Morphology Study of B. cinerea

Scanning electron microscopy (SEM) was performed to observe the morphology of *B. cinerea*. The SEM samples were processed according to the reported methods [37]. Briefly, mycelia blocks (5 mm × 5 mm) were cut from the culture of *B. cinerea* on potato dextrose broth (PDB) on the fifth day after treatment with **BAD-15** at 50 μg/mL and blank control (0.5% DMF). All specimens were fixed in 2.5% glutaraldehyde and rinsed three times with phosphate-buffered saline (PBS, 0.01 M, pH 7.0). Subsequently, samples were post-fixed in 1% osmium tetroxide (*v*/*v*), dehydrated through an ascending ethanol series, and subjected to critical-point drying using CO_2_. Following sputter-coating with gold, images were acquired using a Hitachi Regulus 8100 scanning electron microscope (Tokyo, Japan).

### 2.5. Transcriptome Analyses

RNA extraction was performed following a previously reported protocol [36]. A stock solution of the target compound **BAD-15** (25,000 µg/mL) was prepared in dimethyl sulfoxide, and 100 µL of this stock solution was added to 4900 µL of sterile water containing 0.1% Tween 80 to obtain a working solution at a concentration of 500 µg/mL. To prepare the culture medium with a final concentration of 6.66 µg/mL **BAD-15**, 1 mL of the working solution was mixed with 99 mL of potato dextrose broth (PDB). A control culture containing all components except **BAD-15** was simultaneously established. Subsequently, *B. cinerea* was cultivated in the treated and control PDB media for five days under standard conditions. After incubation, mycelial samples were harvested by filtration, immediately frozen in liquid nitrogen, and stored at −80 °C until RNA extraction. Each treatment was replicated three times.

Total RNA extraction and RNA-Seq analysis were performed by Beijing BiomarkerTechnologies Co., Ltd. (Beijing, China). The *B. cinerea* genome (assembly accession: GCF_000143535.2) was used as a reference for mapping the short reads. The data was analyzed on the online platform of BMKCloud (www.biocloud.net). Differential expression analysis of two conditions was performed using DESeq2 v.1.30.1. DESeq2 provides statistical routines for determining differential expression in digital gene expression data using a model based on the negative binomial distribution. The resulting *p* values were adjusted using Benjamini and Hochberg’s approach for controlling the false discovery rate. Genes with an adjusted Fold Change ≥ 2 and FDR < 0.01 found by DESeq2 v.1.30.1 were assigned as differentially expressed. Gene Ontology (GO) (http://www.geneontology.org, accessed on 26 December 2025) enrichment analysis of the differentially expressed genes (DEGs) was implemented by the clusterProfiler v.4.4.4 packages based on Wallenius noncentral hyper-geometric distribution. KOBAS database and clusterProfiler v.4.4.4 software were used to test the statistical enrichment of DEGs in Kyoto Encyclopedia of Genes and Genomes (KEGG) (http://www.genome.jp/kegg/, accessed on 26 December 2025) pathways.

### 2.6. Statistical Analysis

The values presented in each table represent the mean ± standard deviation (SD) derived from at least three repeated experiments. Statistical analysis was performed using the DPS 7.05 data processing system (DPS, Hangzhou, China).

## 3. Results and Discussion

### 3.1. Chemistry

The synthesis of target compounds **BAD-1** to **BAD-33** is outlined in Scheme 1. The route consists of five consecutive steps, starting from commercially available 3-bromo-2-oxopropanoic acid and ethyl amino(thioxo)acetate as the initial materials. First, intermediate **1** was prepared via a cycloaddition reaction between these two reagents, affording a yield of 73.1%. Subsequently, intermediate **1** underwent acyl chlorination using thionyl chloride, followed by condensation with tryptamine to afford intermediate **2** in an overall yield of 70.3%. Intermediate **2** was then refluxed with hydrazine hydrate in ethanol to yield key intermediate **3** in a yield of 88.1%. Finally, the target compounds **BAD-1** to **BAD-33** were synthesized through a Schiff base reaction of intermediate **3** with various aldehyde reagents, yielding products in the range of 48.5% to 95.5%. The structures of all synthesized compounds were characterized by ^1^H NMR, ^13^C NMR, and HRMS (Figures S1–S99).

### 3.2. Fungicidal Activity and SAR

The in *vitro* antifungal activities of the target compound **BAD-1** to **BAD-33** against seven phytopathogenic fungi (i.e., *A. solani*, *B. cinerea*, *F. graminearum*, *P. piricola*, *R. solani*, *S. sclerotiorum*, and *F. oxysporum*) at a concentration of 50 μg/mL are summarized in Table 1. Thifluzamide, a commercially available fungicide, was used as positive control. It was observed that the majority of the target compounds exhibited antifungal activity against the tested fungal strains, although their potency was suboptimal. Compared with thifluzamide, most compounds showed reduced antifungal activity against *A. solani*, *F. graminearum*, *P. piricola*, *R. solani*, *F. verticillioides*, and *F. oxysporum*, but demonstrated superior antifungal efficacy against *B. cinerea*. For example, some compounds, such as **BAD-9**, **BAD-14**, **BAD-15**, **BAD-17**, and **BAD-18**, exhibited pronounced antifungal activity against *B. cinerea*, with inhibition rates exceeding 70%, surpassing that of thifluzamide (inhibition rate = 37%). One plausible explanation for this selectivity is the differential capacity of fungi to metabolize distinct fungicides [38]. These preliminary results indicated that the title compounds were successfully designed.

**Figure 2:**
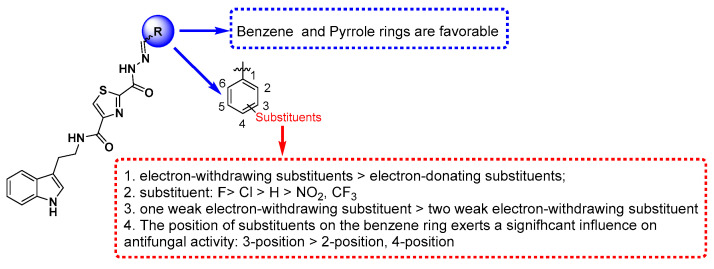
The summarized SAR analysis.

Based on the antifungal activity results against *B. cinerea*, a preliminary structure–activity relationship (SAR) analysis was performed. Comparative evaluation of the antifungal activities of compounds **BAD-1** to **BAD-10** revealed that compounds bearing a benzene ring (**BAD-1**) or a pyrrole ring (**BAD-9**) at the R position demonstrated superior antifungal potency relative to those containing other heterocyclic moieties. Additionally, it was observed that the substituents on the benzene ring exert a significant influence on antifungal activity. In general, compounds bearing electron-withdrawing groups on the benzene ring exhibited higher antifungal activity than their analogues containing electron-donating substituents. For instance, compounds **BAD-11** (R = 2-CF_3_ phenyl), **BAD-14** (R = 2-F phenyl), **BAD-17** (R = 2-Cl phenyl), and **BAD-20** (R = 2-Br phenyl) displayed inhibition rates of 55%, 73%, 72%, and 48%, respectively, whereas compounds **BAD-23** (R = 2-OMe phenyl) and **BAD-27** (R = 2-OH phenyl) showed only 16% and 15%. Notably, compared to compound **BAD-1**, derivatives bearing strong electron-withdrawing groups such as CF_3_ (**BAD-11** to **BAD-13**) or NO_2_ (**BAD-26**) exhibited reduced antifungal activity, while those incorporating weak electron-withdrawing groups such as F (**BAD-14** to **BAD-17**) or Cl (**BAD-18** to **BAD-20**) demonstrated enhanced potency. The plausible reason is that the introduction of fluorine or chlorine atoms enables precise modulation of compound partitioning between aqueous and lipid phases [39,40]. Owing to their lower polarizability, C–F and C–Cl bonds increase lipophilicity and confer enhanced thermal stability to the parent molecule—properties that synergistically improve cellular bioavailability and thereby potentiate biological activity [41]. Furthermore, compounds with weak electron-withdrawing groups at the 3-position of the benzene ring displayed superior antifungal activity relative to their 2- or 4-substituted counterparts. For example, compound **BAD-15** (R = 3-F phenyl) achieved an inhibition rate of 89%, surpassing those of **BAD-14** (R = 2-F phenyl, 73%) and **BAD-16** (R = 4-F phenyl, 69%). Additionally, analogues bearing two chlorine atoms on the benzene ring (i.e., **BAD-28** to **BAD-33**) exhibited lower antifungal activity compared to those with a single chlorine atom. These analysis results indicate that the introduction of one weak electron-withdrawing group on the benzene ring effectively enhances antifungal activity, with substitution at the 3-position being the optimal configuration. Overall, these SAR analysis results provide valuable insights for guiding the design of compounds with improved antifungal activity. The summarized SAR analysis is shown in Figure 2.

To further investigate the antifungal potential of the target compound against *B. cinerea*, compounds with inhibition rates exceeding 65% were selected for further evaluation of their EC_50_ values. As shown in Table 2, compounds **BAD-15** and **BAD-18** demonstrated promising antifungal activity against *B. cinerea* with EC_50_ values of 6.725 and 12.05 μg/mL, respectively. These results indicate that the bacillamide–acylhydrazone scaffold has potential as a lead structure for the development of fungicides, and compound **BAD-15** can serve as a lead compound for further optimization.

**Figure 3:**
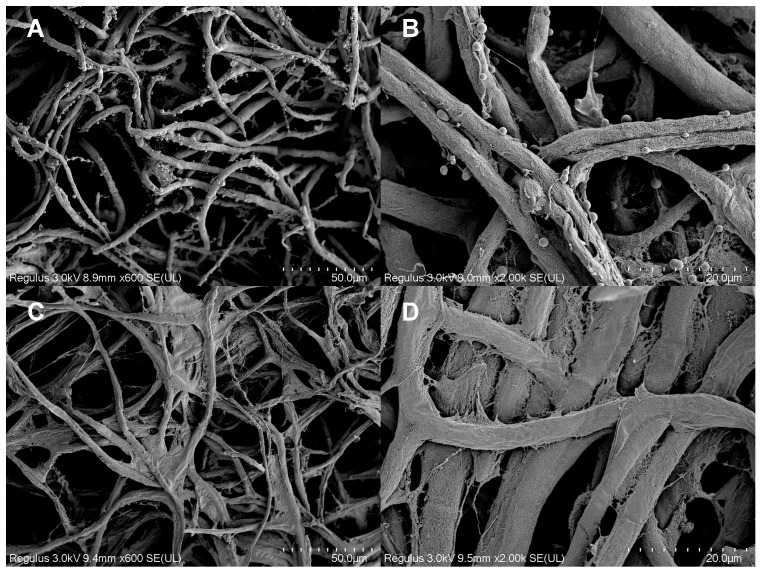
Scanning electron micrographs of *B. cinerea* mycelium ((**A**,**B**): control; (**C**,**D**): treated with **BAD-15**).

To investigate the antifungal mechanism of the target compound, **BAD-15** was selected as a representative candidate to examine its molecular mode of action. Morphological changes in *B. cinerea* following treatment with **BAD-15** were preliminarily analyzed using SEM. The results revealed that, in contrast to the smooth, uniform, and elongated hyphae observed in untreated controls (Figure 3A), **BAD-15**-treated mycelia exhibited pronounced morphological alterations, including shrinkage, excessive branching, and loss of structural integrity (Figure 3C). Furthermore, a significant reduction in spore production was observed in **BAD-15**-treated *B. cinerea* compared to the control group (Figure 3B,D). These findings indicate that **BAD-15** may compromise the structural integrity of the fungal cell wall and plasma membrane and suppress spore development.

**Figure 4:**
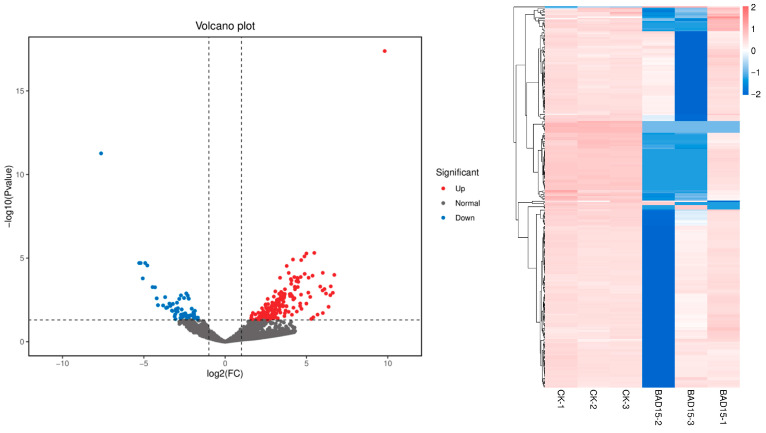
Transcriptome analysis of *B. cinerea* treated by **BAD-15**. (**Left**) volcano plots; (**right**) heatmap.

**Figure 5:**
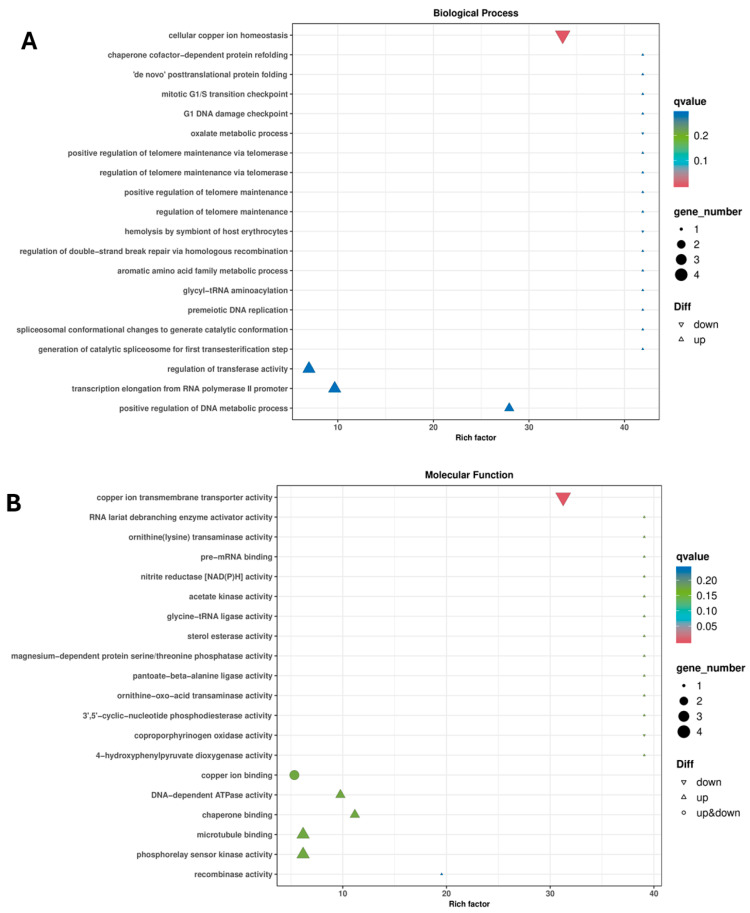

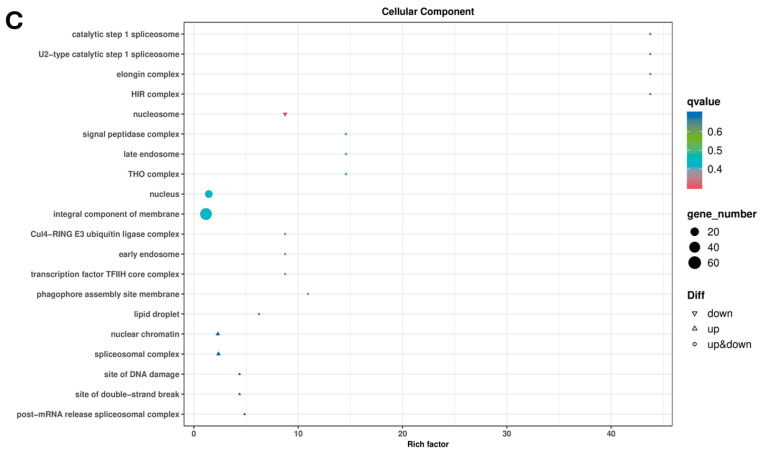
GO analysis of DEGs. (**A**): the biological process; (**B**): the molecular function; (**C**): the cellular component.

To further explore the molecular mechanism of action, RNA sequencing was performed to identify DEGs in *B. cinerea* following treatment with compound **BAD-15**. Transcriptome analysis revealed that a total of 259 genes exhibited differential expression, including 193 upregulated and 66 downregulated genes (Figure 4). Subsequently, these DEGs were subjected to GO and KEGG annotation analyses. The GO enrichment histogram provides a comprehensive overview of the enrichment and distribution of DEGs across the three main categories: biological process (BP), molecular function (MF), and cellular component (CC). As shown in Figure 5, in the biological process category (Figure 5A), the DEGs were significantly enriched in the term “cellular copper ion homeostasis” and exhibited down-regulation, indicating impairment of the regulatory mechanism underlying intracellular copper ion homeostasis. As reported, copper homeostasis is a tightly regulated process critical for the maintenance of cellular function [42,43]. Previous studies have revealed that copper serves as an essential enzymatic cofactor in diverse biological processes, including cellular respiration (cytochrome-c oxidase), antioxidant defence (superoxide dismutase), melanin biosynthesis (tyrosinase), extracellular matrix maturation (lysyl oxidase), and iron homeostasis (e.g., via multicopper ferroxidases) [44,45,46]. However, copper accumulation beyond physiological capacity induces cytotoxicity. Excess free cytosolic copper ions disrupt cellular metalloprotein homeostasis through three primary mechanisms: (i) competitive displacement of other essential metal ions (e.g., Zn^2+^, Fe^2+^) from metalloenzyme active sites; (ii) aberrant binding to intracellular metallophilic ligands—particularly labile iron–sulfur clusters—causing their disassembly; and (iii) catalysis of hydroxyl radical generation via Fenton- and Haber–Weiss-type reactions, resulting in oxidative damage to lipids, proteins, and DNA. This dual nature—indispensable as a micronutrient yet inherently toxic at elevated concentrations—necessitates highly precise, multi-layered regulatory systems to maintain intracellular copper homeostasis [47,48]. In the molecular function category (Figure 5B), the DEGs were significantly enriched in the term “copper ion transmembrane transporter activity” and exhibited down-regulation, indicating that the transmembrane transporter of copper ions was markedly impaired. Copper ion transmembrane transporters play a critical role in regulating copper uptake and efflux. Dysfunction of these transporters directly disrupts copper homeostasis, physiological metabolism, and environmental adaptability in fungi [49,50]. Downregulation of copper uptake transporter gene expression impairs the ability of fungi to acquire copper from the environment, leading to loss of activity in copper-dependent enzymes, disruption of mitochondrial respiration, energy deficiency, reduced growth rates, and diminished colony size [51]. Concurrently, fungal antioxidant capacity is compromised, resulting in intracellular accumulation of reactive oxygen species and subsequent oxidative damage, including lipid peroxidation of cell membranes and DNA damage [52,53]. In the cellular component category (Figure 5C), the DEGs were significantly enriched in the terms of catalytic step 1 spliceosome, U2-type catalytic step 1 spliceosome, elongin complex, HIR complex, and nucleosome. Based on the GO annotation analysis, compound **BAD-15** may inhibit the activity of copper ion transmembrane transporters, leading to disrupted intracellular copper ion homeostasis and subsequent suppression of fungal growth.

**Figure 6:**
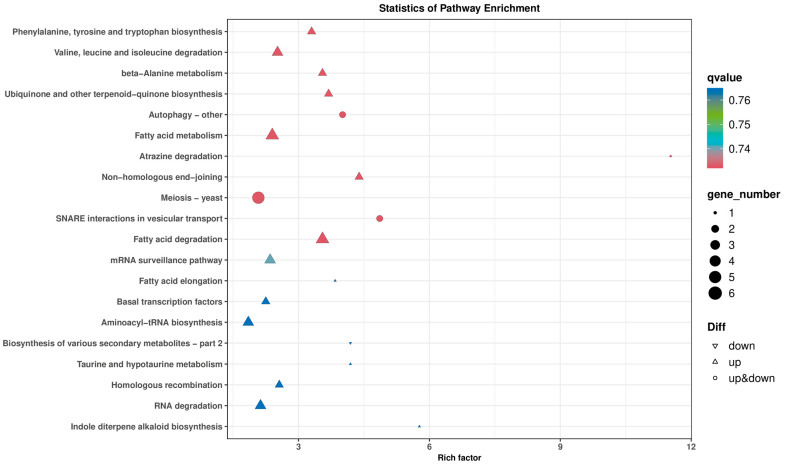
KEGG pathway analysis of DEGs.

Furthermore, KEGG pathway enrichment analysis was performed to identify the primary metabolic pathways associated with the DEGs. As shown in Figure 6, the enriched metabolic pathways, such as phenylalanine, tyrosine, and tryptophan biosynthesis; valine, leucine, and isoleucine degradation; beta-alanine metabolism; ubiquinone and other terpenoid-quinone biosynthesis; fatty acid metabolism and degradation; and non-homologous end joining, are all significantly enriched with up-regulated DEGs. It is well established that phenylalanine, tyrosine, and tryptophan are essential amino acids required for fungal growth and serve as key precursors for a wide range of secondary metabolites [54]. The upregulation of genes associated with this biosynthetic pathway may indicate that the fungus is undergoing rapid proliferation or enhancing the production of secondary metabolites in response to environmental stress [55]. In this study, **BAD-15** inhibited the growth of *B. cinerea*. The upregulation of genes involved in the phenylalanine, tyrosine, and tryptophan biosynthesis pathways indicates that *B. cinerea* mounts a stress response upon exposure to the compound **BAD-15**. Under external stress conditions, the expression of genes associated with the degradation pathways of valine, leucine, and isoleucine is significantly upregulated, indicating that the fungus may generate energy through the catabolism of stored branched-chain amino acids while simultaneously producing essential intermediate metabolites required for the maintenance of cellular functions [56]. β-alanine, a non-proteinogenic amino acid, plays a central role in two critical biological processes: coenzyme A biosynthesis and the regulation of osmotic balance and oxidative stress responses [57]. Ubiquinone, a key component of the mitochondrial electron transport chain, mediates electron transfer and ATP synthesis [58]. The activation of the ubiquinone and other terpenoid-quinone biosynthesis pathways directly reflects a substantial increase in fungal energy demand—necessary not only to support high-energy-consuming processes such as DNA repair and amino acid synthesis, but also to sustain energy homeostasis under stress and prevent energy deficiency-induced apoptosis [59]. Furthermore, terpenoid-quinone compounds contribute to cellular stability by stabilizing membrane structure and enhancing stress tolerance [60]. Additionally, the significant upregulation of genes involved in fatty acid metabolism and degradation pathways suggests that the fungus adapts to environmental stresses, including antifungal agents and other chemical challenges, by modulating its fatty acid composition—a regulatory mechanism that supports both membrane integrity and cellular energy homeostasis [61]. Non-homologous end joining (NHEJ) is a primary pathway responsible for the repair of DNA double-strand breaks in eukaryotes. The upregulation of genes involved in this pathway provides direct evidence of significant DNA damage stress [62]. Given the above analysis results, the coordinated upregulation of these pathways indicates that the fungus is under complex stress from the compound **BAD-15**, requiring it to maintain both survival and metabolic homeostasis simultaneously. To achieve this goal, the fungus synthesizes key amino acids to support secondary metabolism and protein synthesis, while degrading stored amino acids to provide energy; it enhances the efficiency of ATP synthesis, eliminates reactive oxygen species, and remodels the cell membrane structure to improve its adaptability to environmental stress; in addition, the fungus activates the NHEJ pathway to rapidly repair DNA damage and ensure genomic stability, thereby comprehensively addressing multiple physiological challenges.

Although the above transcriptome analysis provides valuable insights into DEG, it has inherent limitations for mechanistic investigation. Specifically, it permits only the identification and functional enrichment of dysregulated genes but cannot resolve deeper mechanistic layers—including the regulatory signalling pathways governing key genes, their upstream regulators and downstream effectors, or post-translational modifications that critically modulate protein function, stability, and subcellular localization. Our future work will integrate complementary multi-omics approaches—namely, quantitative proteomics, phosphoproteomics, and metabolomics—to systematically dissect the precise molecular mechanisms underlying the biological effects of the compound **BAD-15** that we have observed.

## 4. Conclusions

In summary, 33 novel bacillamide–acylhydrazone derivatives were successfully designed and synthesized through conjugation of the bacillamide scaffold with an acylhydrazone moiety. Antifungal assays demonstrated that most of the target compounds exhibited promising inhibitory activity against *B. cinerea*. Notably, compounds **BAD-15** and **BAD-18** displayed potent antifungal activity, with EC_50_ values of 6.725 and 12.05 μg/mL, respectively. Preliminary SAR analysis revealed that the R group exerts a significant influence on antifungal potency. Derivatives bearing a benzene ring or pyrrole ring at the R position exhibited superior activity compared to those containing other heterocyclic moieties, and the introduction of a weak electron-withdrawing group at the 3-position of the benzene ring was found to enhance antifungal efficacy. Furthermore, morphological studies and transcriptome analyses were conducted to preliminarily investigate the molecular mechanism of action of compound **BAD-15**. The SEM analysis results indicated that **BAD-15** may compromise the structural integrity of the fungal cell wall and plasma membrane and suppress spore development. The transcriptome analysis results demonstrated that **BAD-15** may inhibit the activity of copper ion transmembrane transporters, leading to disrupted intracellular copper ion homeostasis and subsequent suppression of fungal growth. This research is expected to provide critical theoretical foundations and innovative insights for the development of novel and highly efficient fungicides. Ongoing efforts in our laboratory are focused on structural optimization and elucidation of the molecular mechanism of action of the compound **BAD-15**.

## Figures and Tables

**Table 1:** The in *vitro* antifungal activities of the target compound **BAD-1** to **BAD-33** at a concentration of 50 μg/mL (% inhibition) ^1^.

Comp.	*A. s* ^2^	*B. c* ^2^	*F. g* ^2^	*P. p* ^2^	*R. s* ^2^	*F. v* ^2^	*F. o* ^2^
**BAD-1**	22 ± 1	67 ± 0	36 ± 4	23 ± 1	2 ± 4	26 ± 2	16 ± 1
**BAD-2**	2 ± 2	44 ± 2	40 ± 2	16 ± 2	9 ± 1	15 ± 3	3 ± 1
**BAD-3**	0	48 ± 2	26 ± 2	24 ± 1	12 ± 4	22 ± 2	8 ± 3
**BAD-4**	25 ± 2	28 ± 1	7 ± 4	7 ± 2	0	16 ± 3	14 ± 2
**BAD-5**	19 ± 2	34 ± 2	45 ± 4	36 ± 2	7 ± 4	36 ± 2	28 ± 1
**BAD-6**	16 ± 4	36 ± 4	2 ± 3	0	13 ± 4	8 ± 3	0
**BAD-7**	14 ± 1	45 ± 1	17 ± 5	28 ± 2	0	28 ± 3	8 ± 1
**BAD-8**	4 ± 4	36 ± 3	1 ± 2	0	1 ± 1	13 ± 1	13 ± 3
**BAD-9**	4 ± 5	76 ± 1	22 ± 1	20 ± 2	2 ± 3	27 ± 1	20 ± 2
**BAD-10**	59 ± 0	47 ± 1	38 ± 3	22 ± 2	0	1 ± 1	0
**BAD-11**	10 ± 1	55 ± 3	21 ± 4	18 ± 0	2 ± 3	9 ± 4	23 ± 1
**BAD-12**	8 ± 1	60 ± 3	37 ± 5	22 ± 1	4 ± 3	15 ± 3	21 ± 1
**BAD-13**	15 ± 3	49 ± 1	21 ± 2	17 ± 1	9 ± 3	17 ± 4	24 ± 2
**BAD-14**	9 ± 1	73 ± 2	18 ± 1	20 ± 1	10 ± 3	6 ± 4	12 ± 2
**BAD-15**	12 ± 2	89 ± 1	29 ± 5	20 ± 0	1 ± 2	18 ± 3	20 ± 1
**BAD-16**	11 ± 2	69 ± 2	19 ± 1	28 ± 1	8 ± 3	19 ± 2	8 ± 2
**BAD-17**	12 ± 1	72 ± 2	17 ± 2	12 ± 0	2 ± 3	10 ± 4	0
**BAD-18**	13 ± 2	80 ± 2	14 ± 6	15 ± 1	12 ± 3	11 ± 3	1 ± 1
**BAD-19**	3 ± 1	65 ± 1	23 ± 3	20 ± 1	5 ± 4	13 ± 3	0
**BAD-20**	8 ± 1	48 ± 2	13 ± 7	14 ± 1	13 ± 3	7 ± 4	0
**BAD-21**	14 ± 2	56 ± 3	7 ± 3	11 ± 1	15 ± 4	9 ± 5	0
**BAD-22**	15 ± 2	35 ± 3	20 ± 5	19 ± 1	21 ± 3	10 ± 4	0
**BAD-23**	6 ± 1	16 ± 3	15 ± 6	8 ± 1	21 ± 4	3 ± 3	0
**BAD-24**	13 ± 1	33 ± 2	18 ± 5	8 ± 1	3 ± 4	7 ± 4	0
**BAD-25**	8 ± 1	26 ± 4	23 ± 6	24 ± 1	10 ± 3	15 ± 3	4 ± 3
**BAD-26**	9 ± 1	12 ± 3	19 ± 7	0	10 ± 5	7 ± 4	0
**BAD-27**	5 ± 2	15 ± 4	29 ± 5	6 ± 1	13 ± 4	5 ± 4	0
**BAD-28**	1 ± 1	36 ± 1	1 ± 1	0	0	5 ± 2	10 ± 2
**BAD-29**	6 ± 4	58 ± 2	1 ± 1	1 ± 1	0	17 ± 2	17 ± 3
**BAD-30**	10 ± 4	33 ± 2	9 ± 1	0	12 ± 5	12 ± 1	9 ± 3
**BAD-31**	10 ± 4	60 ± 3	15 ± 1	8 ± 2	1 ± 1	9 ± 2	9 ± 3
**BAD-32**	1 ± 2	47 ± 1	13 ± 6	13 ± 1	21 ± 2	10 ± 3	3 ± 1
**BAD-33**	8 ± 4	30 ± 1	14 ± 1	1 ± 1	2 ± 3	15 ± 2	7 ± 4
Thifluzamide	65 ± 1	37 ± 3	100	40 ± 3	19 ± 2	18 ± 1	11 ± 0

^1^ Each value represents the mean ± SD of three experiments. ^2^ *A. solani* (*A. s*), *B. cinerea* (*B. c*), *F. graminearum* (*F. g*), *P. piricola* (*P. p*), *R. solani* (*R. s*), *F. verticillioides* (*F. v*), and *F. oxysporum* (*F. o*).

**Table 2:** In vitro precision toxicity determination of EC_50_ values of selected compounds.

Comp.	Regression Equation	R^2^	EC_50_ (μg/mL)	95% Confidence Interval (μg/mL)
**BAD-1**	y = 1.284x + 5.468	0.9835	30.15	25.09–37.34
**BAD-9**	y = 1.582x + 4.184	0.9803	22.3	18.47–27.25
**BAD-14**	y = 1.145x + 20.18	0.9517	17.72	12.36–27.62
**BAD-15**	y = 1.352x + 30.13	0.9852	6.725	5.421–8.335
**BAD-16**	y = 1.162x + 13.43	0.9641	25.71	18.96–38.10
**BAD-17**	y = 0.675x + 21.63	0.9533	21.85	15.40–31.85
**BAD-18**	y = 1.318x + 22.33	0.9401	12.05	7.991–18.82
**BAD-19**	y = 1.046x + 14.39	0.9422	30.8	20.87–55.79

## Data Availability

The data are contained within the article; further inquiries can be directed to the corresponding author.

## Supplementary Materials

The following supporting information can be downloaded at https://www.mdpi.com/article/10.3390/jof12030169/s1.
