# Supplementary material for: Discovery of Bacillamide–Acylhydrazone Hybrids as Novel Fungicide Lead Compounds
Source: J Fungi (Basel). 2026 Feb 26;12(3):169. doi: 10.3390/jof12030169 (PMC13027498; doi:10.3390/jof12030169)
Supplement: Supplementary file 1 [file jof-12-00169-s001.zip › jof-4140733-supplementary.pdf]

Discovery of Bacillamide–Acylhydrazone Hybrids as Novel Fungicide Lead Compounds

Sijia Feng [1], Yuxiao Zhang [2], Peipei Shi [1], Ke Chen [2,*] and Kang Lei [2]

[1] College of Life Science, Henan Normal University, Xinxiang 453007, China; 15083111270@163.com (S.F.); shipeipei2018@163.com (P.S.)
[2] School of Pharmaceutical Sciences and Food Engineering, Liaocheng University, Liaocheng 252059, China; zhangyuxiao0515@163.com (Y.Z.); leikang@lcu.edu.cn (K.L.)
* Correspondence: chenke@lcu.edu.cn

1. The ^{1}HNMR, ^{13}CNMR, and HRMS Spectrum of Target Compounds

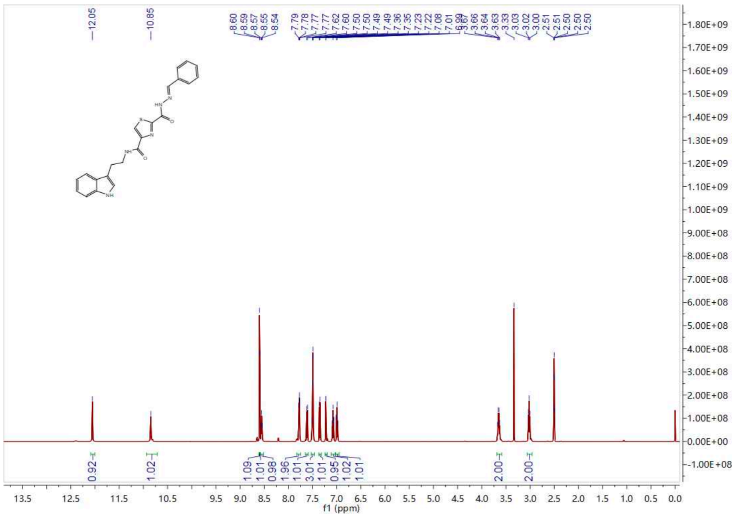

Figure S1 ^{1}H NMR of **BAD-1**

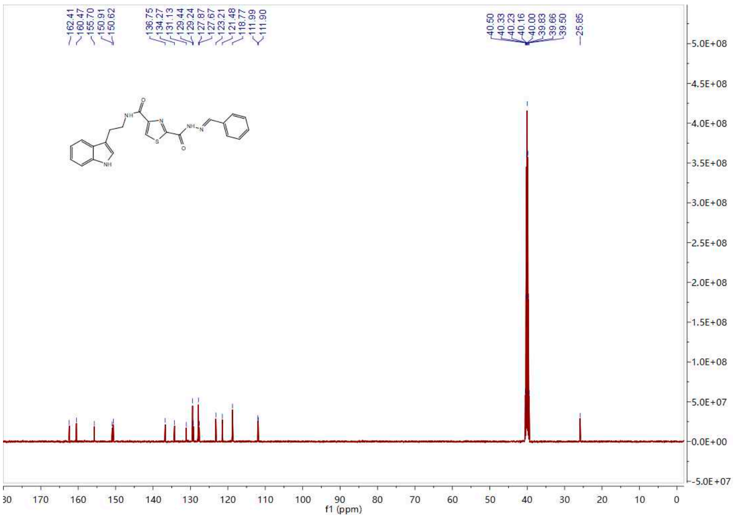

Figure S2 ^{13}C NMR of **BAD-1**

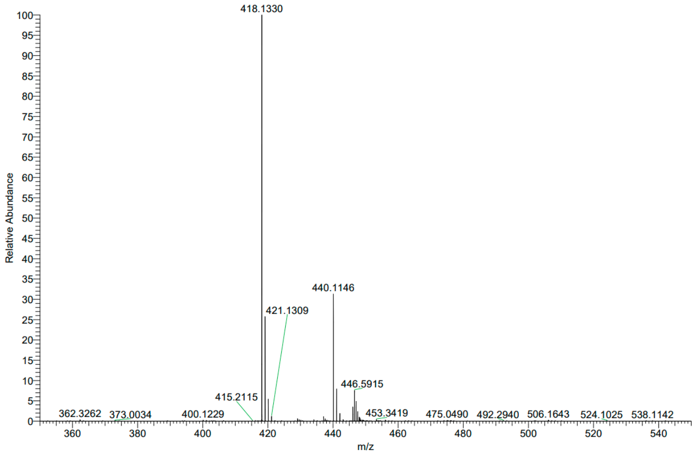

Figure S3 HRMS of **BAD-1**

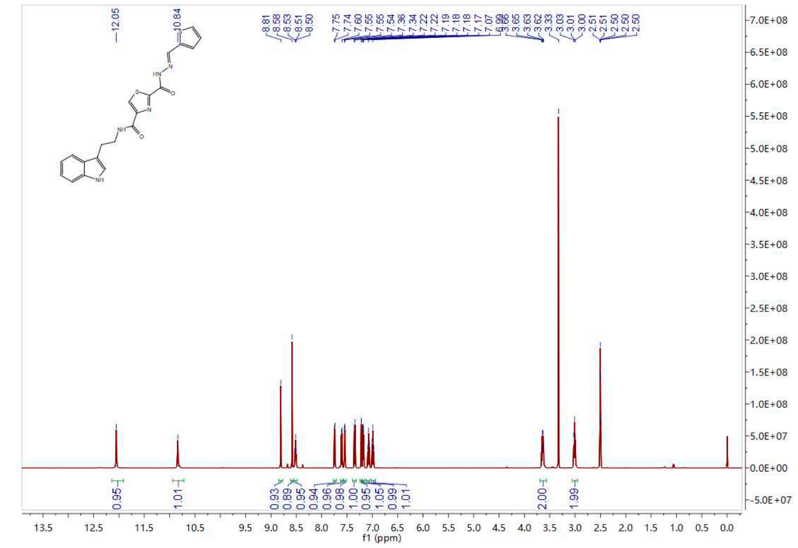

Figure S4 ^{1}H NMR of **BAD-2**

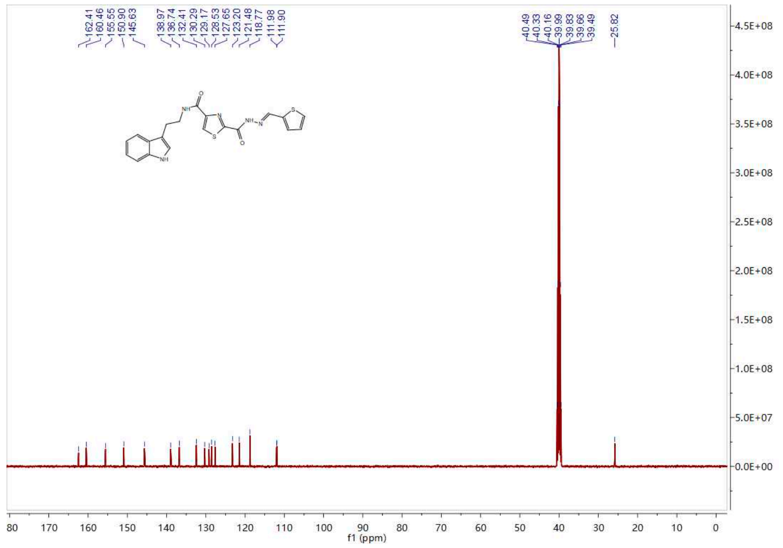

Figure S5 ^{13}C NMR of **BAD-2**

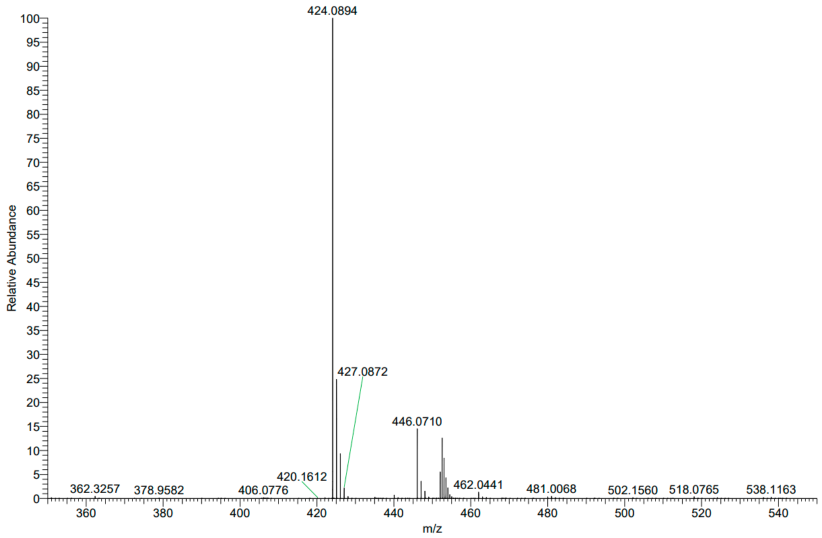

Figure S6 HRMS of **BAD-2**

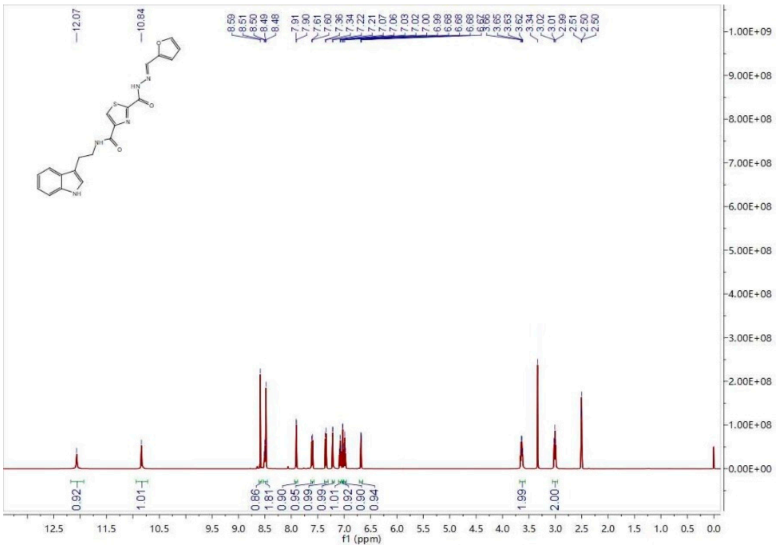

Figure S7 ^{1}H NMR of **BAD-3**

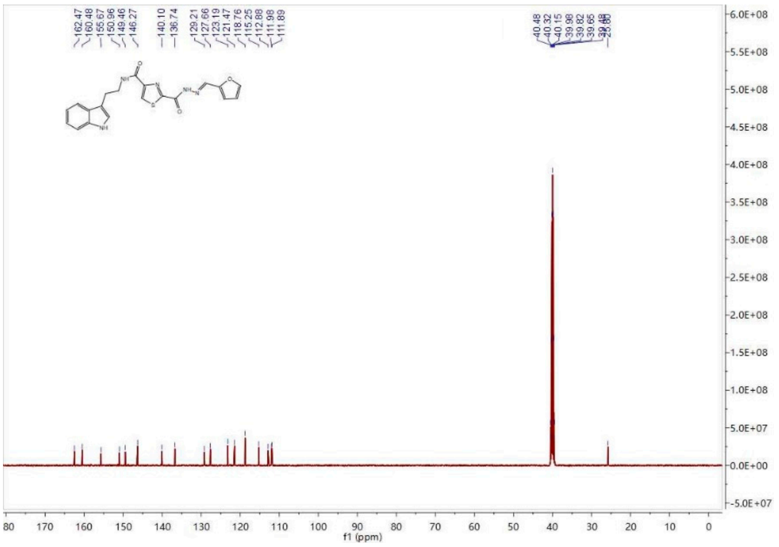

Figure S8 ^{13}C NMR of **BAD-3**

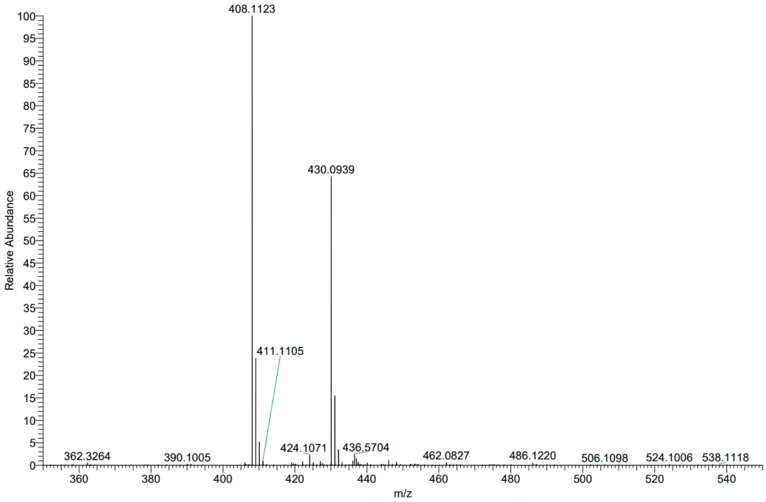

Figure S9 HRMS of **BAD-3**

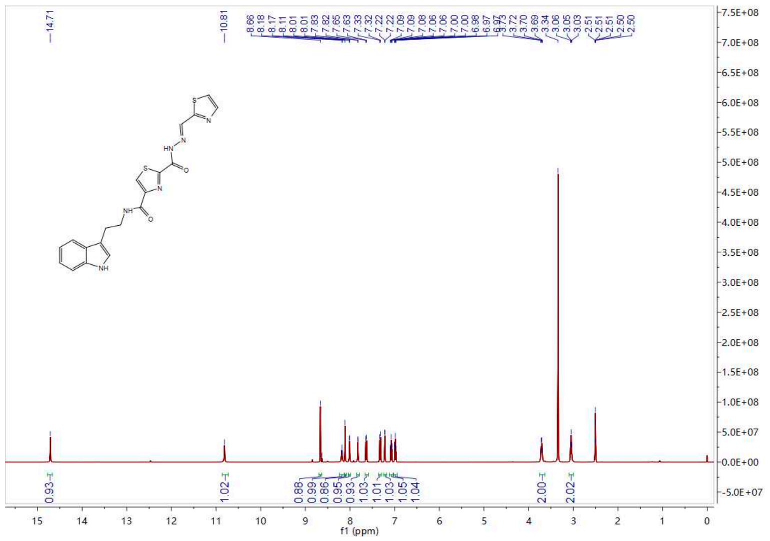

Figure S10 ^{1}H NMR of **BAD-4**

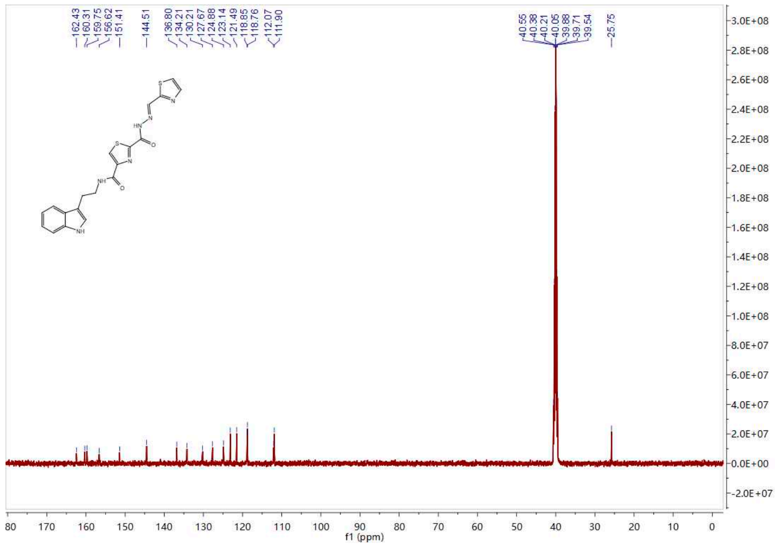

Figure S11 ^{13}C NMR of **BAD-4**

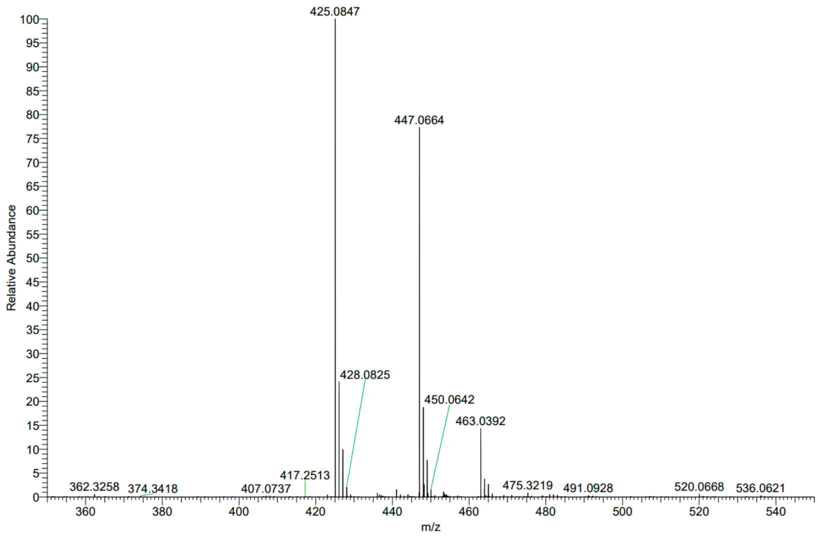

Figure S12 HRMS of **BAD-4**

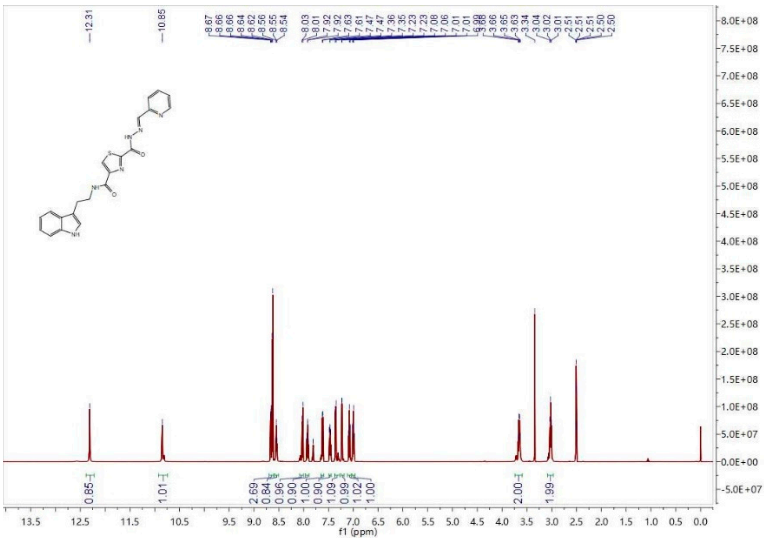

Figure S13 ^{1}H NMR of **BAD-5**

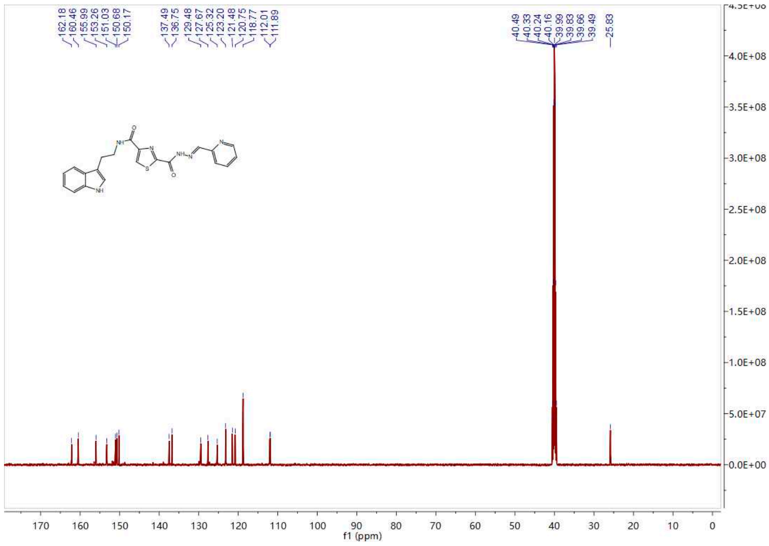

Figure S14 ^{13}C NMR of **BAD-5**

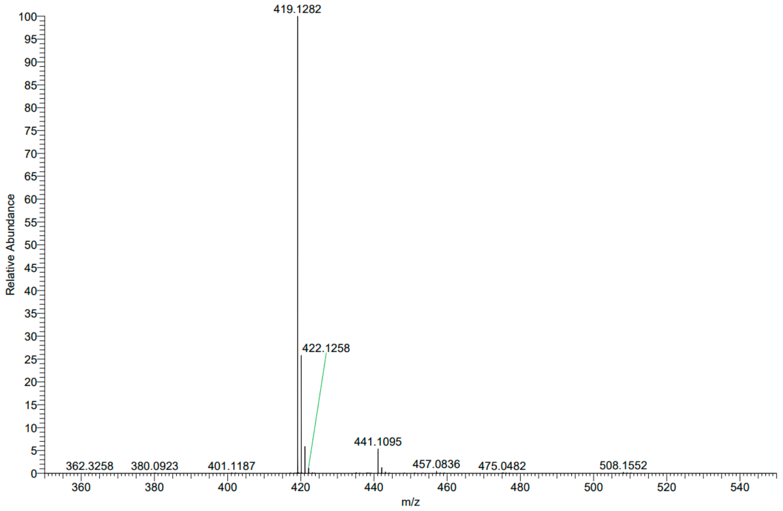

Figure S15 HRMS of **BAD-5**

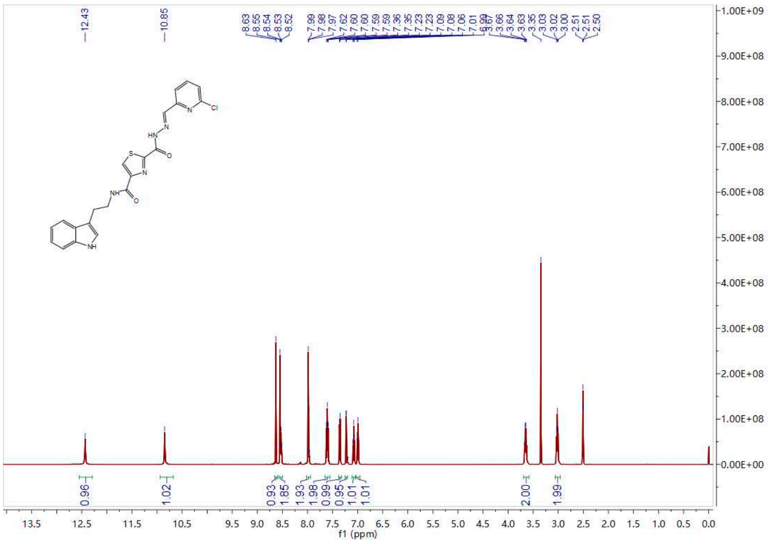

Figure S16 ^{1}H NMR of **BAD-6**

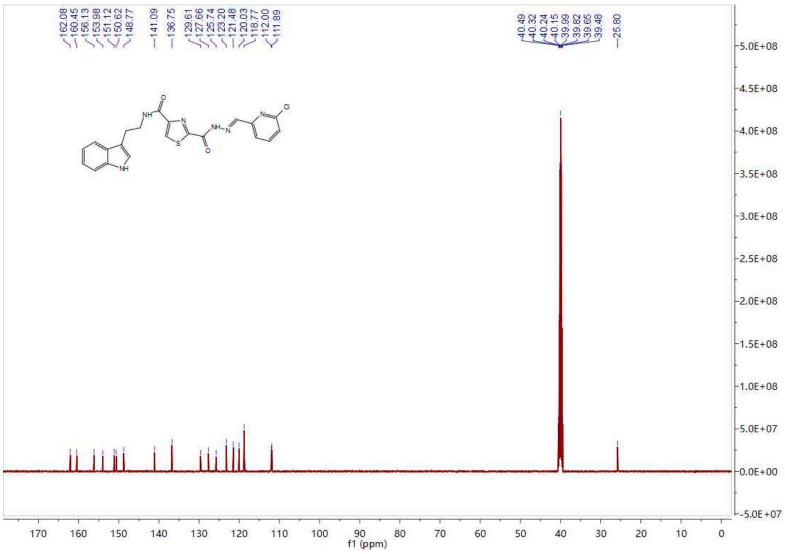

Figure S17 ^{13}C NMR of **BAD-6**

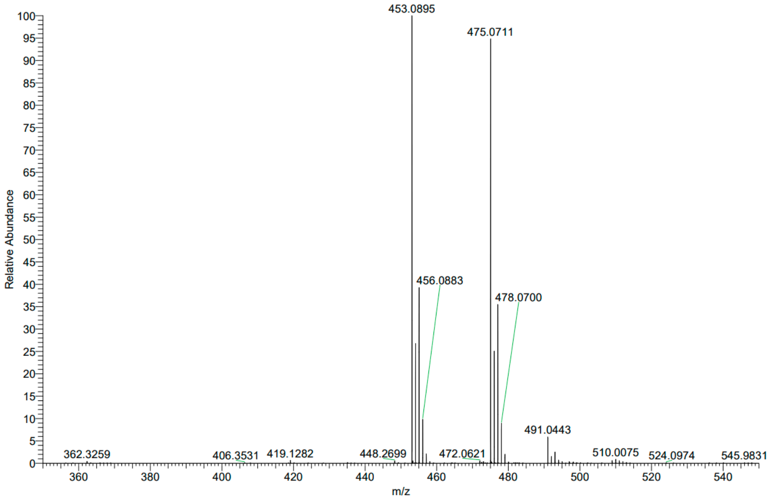

Figure S18 HRMS of **BAD-6**

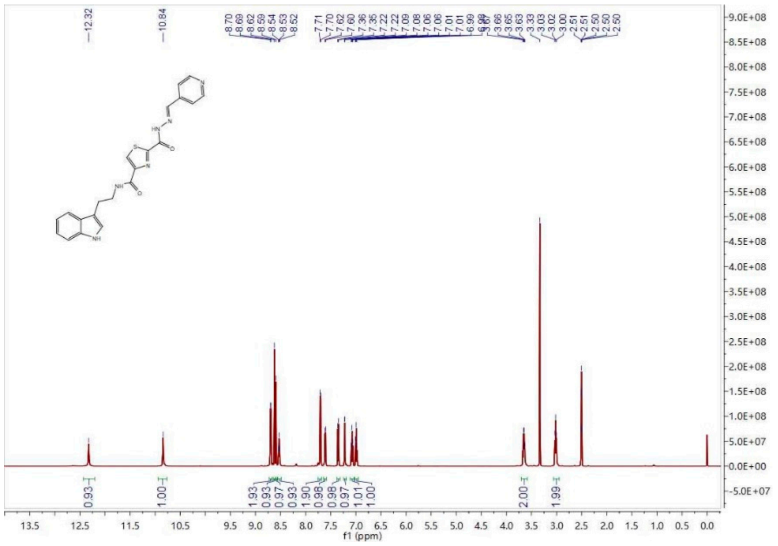

Figure S19 ^{1}H NMR of **BAD-7**

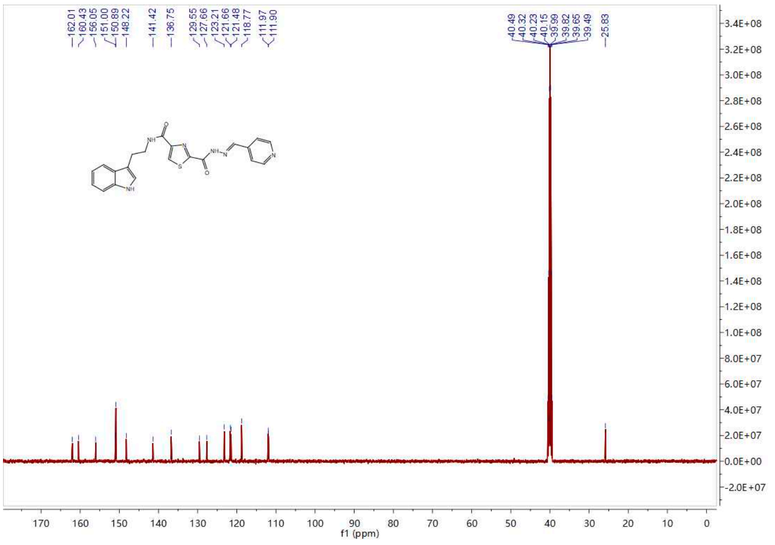

Figure S20 ^{13}C NMR of **BAD-7**

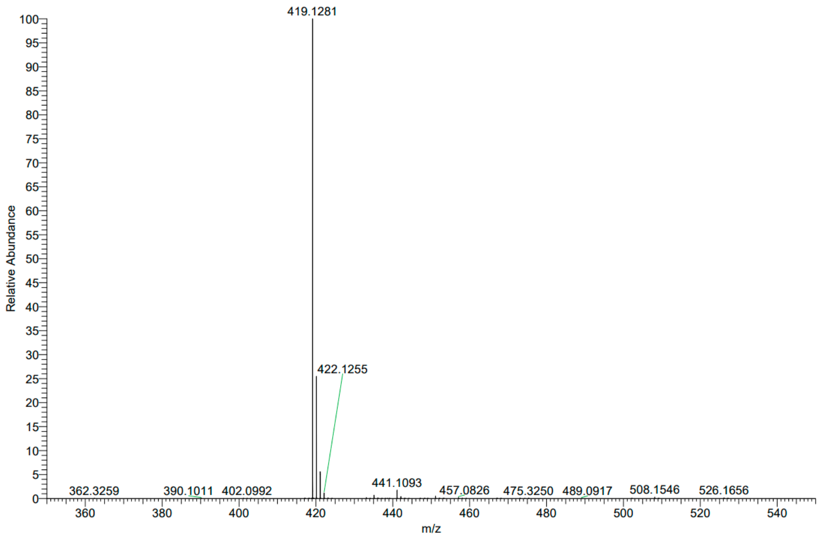

Figure S21 HRMS of **BAD-7**

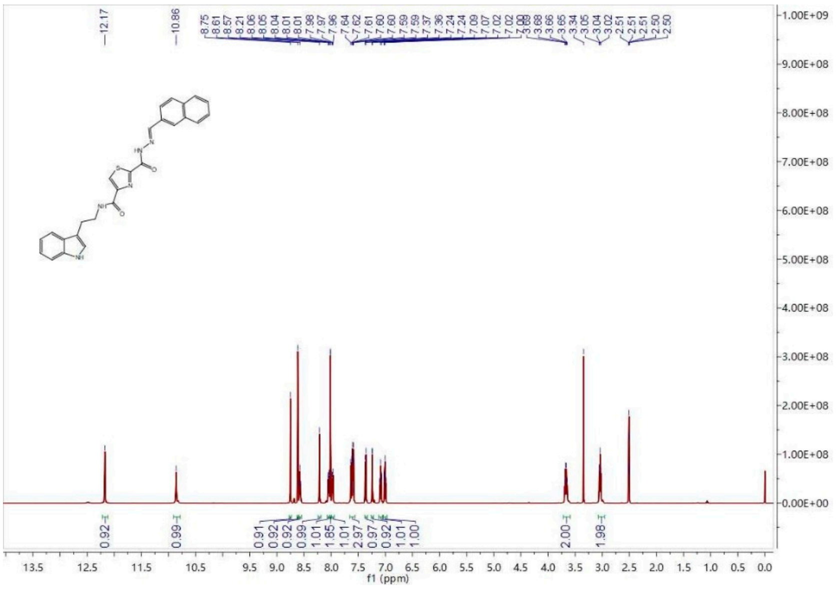

Figure S22 ^{1}H NMR of **BAD-8**

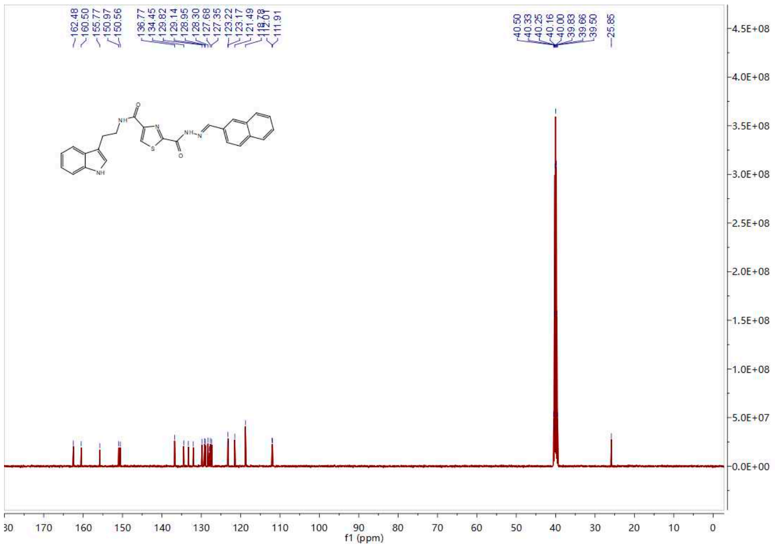

Figure S23 ^{13}C NMR of **BAD-8**

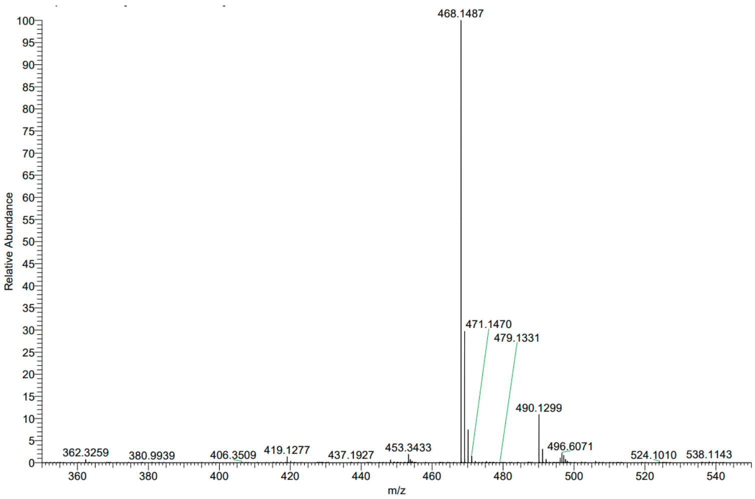

Figure S24 HRMS of **BAD-8**

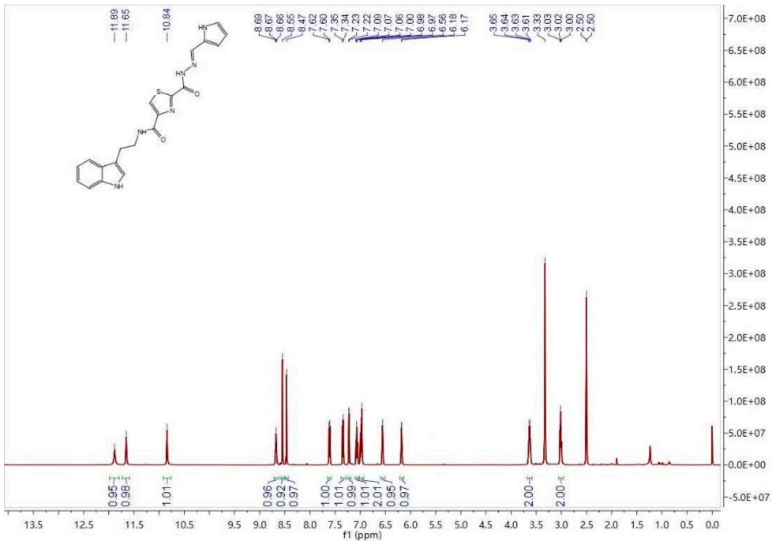

Figure S25 ^{1}H NMR of **BAD-9**

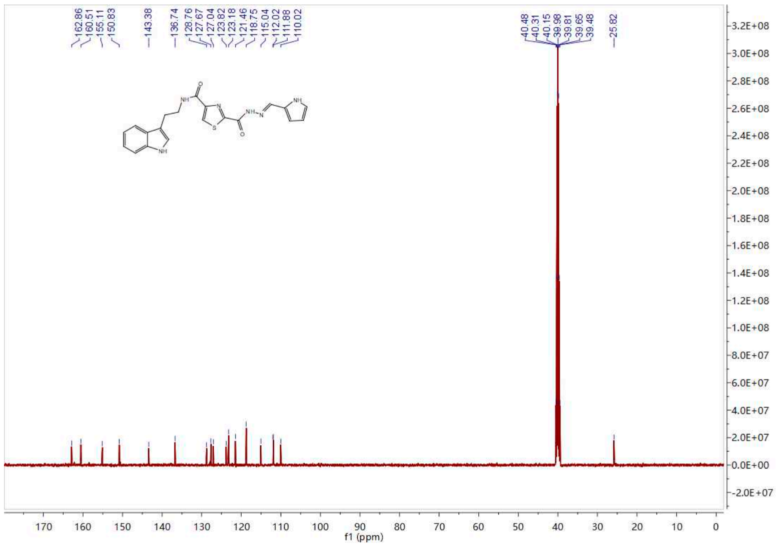

Figure S26 ^{13}C NMR of **BAD-9**

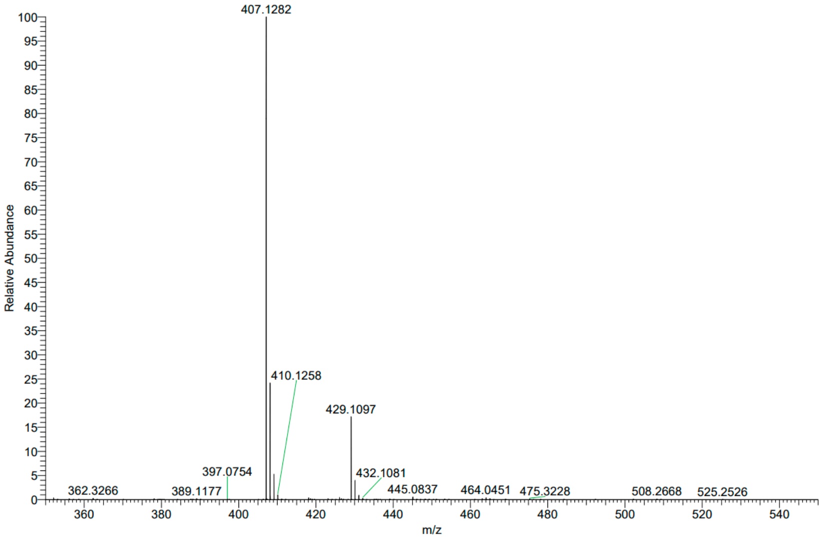

Figure S27 HRMS of **BAD-9**

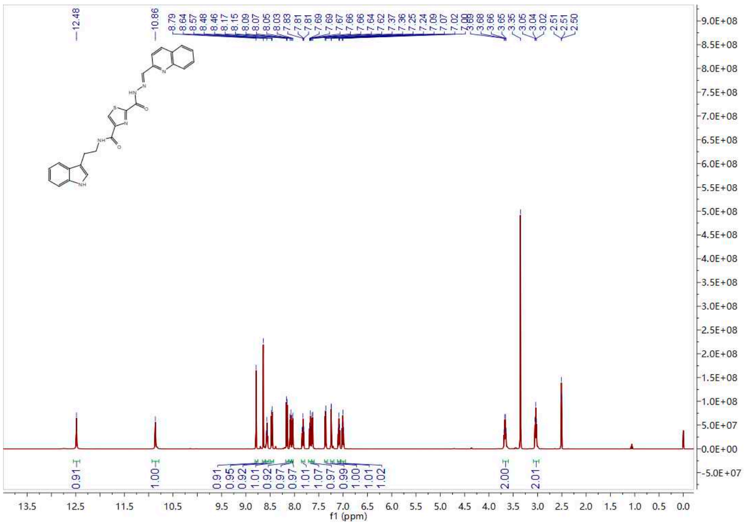

Figure S28 ^{1}H NMR of **BAD-10**

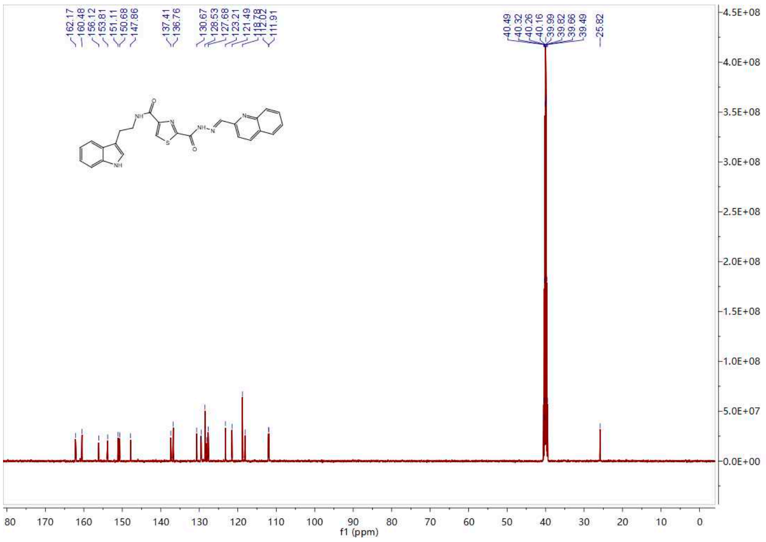

Figure S29 ^{13}C NMR of **BAD-10**

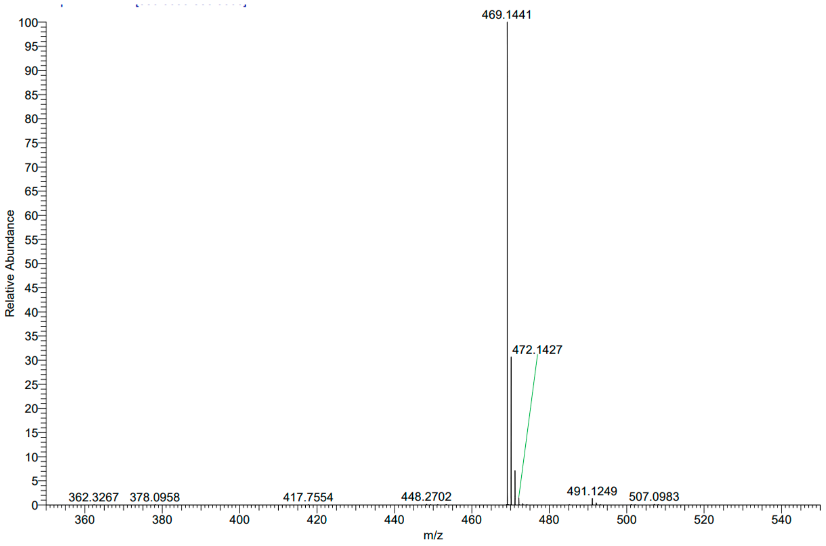

Figure S30 HRMS of **BAD-10**

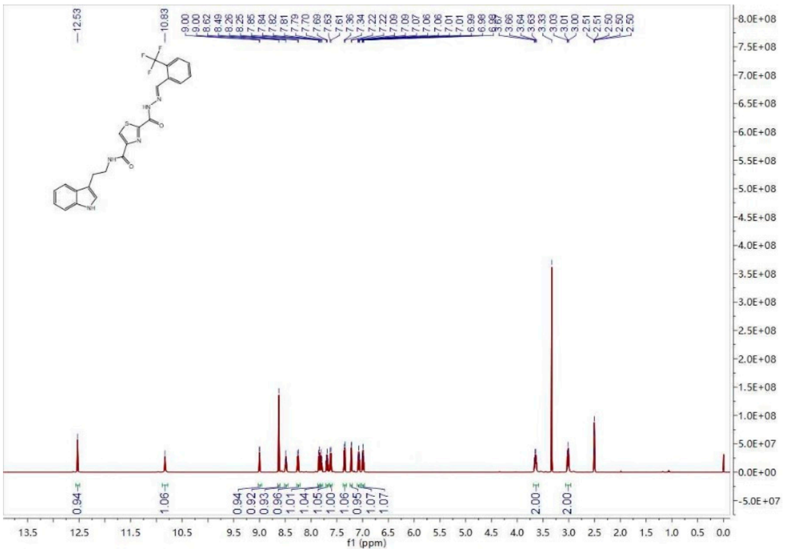

Figure S31 ^{1}H NMR of **BAD-11**

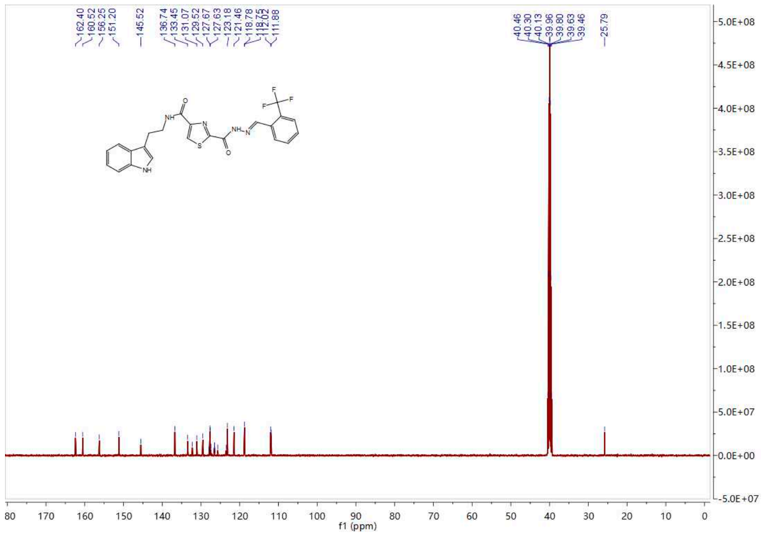

Figure S32 ^{13}C NMR of **BAD-11**

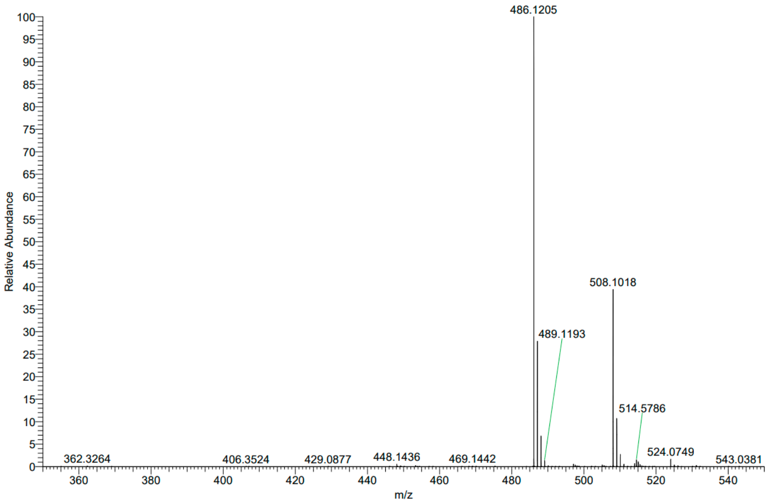

Figure S33 HRMS of **BAD-11**

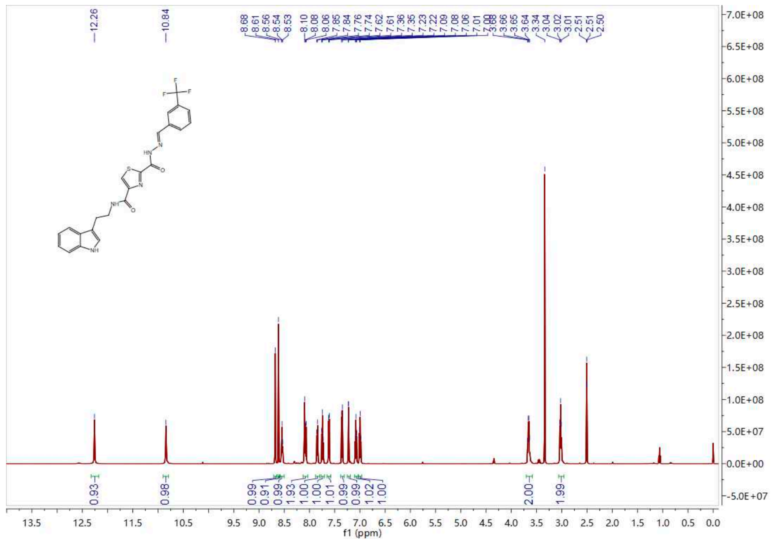

Figure S34 ^{1}H NMR of **BAD-12**

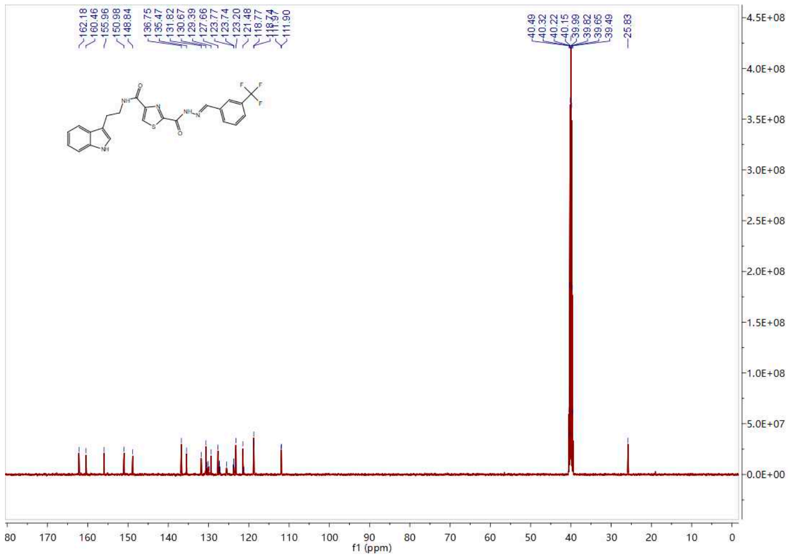

Figure S35 ^{13}C NMR of **BAD-12**

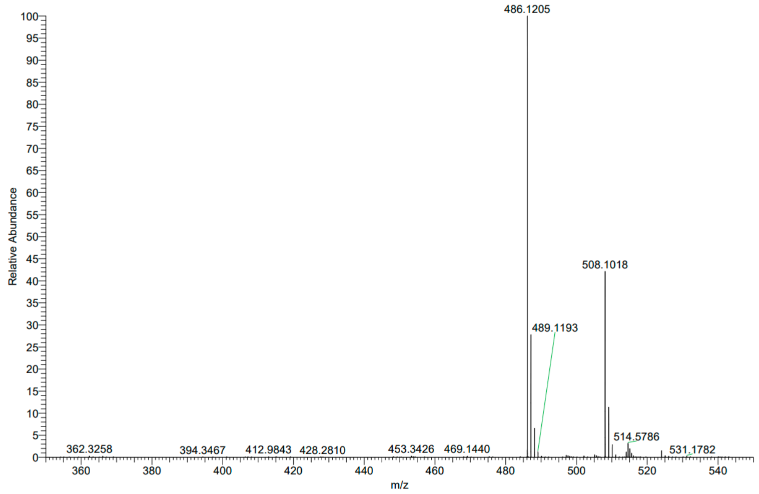

Figure S36 HRMS of **BAD-12**

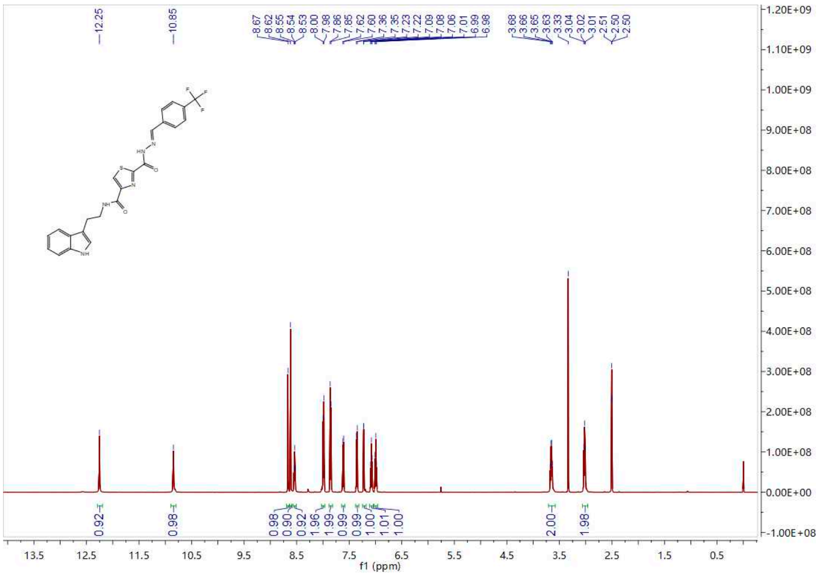

Figure S37 ^{1}H NMR of **BAD-13**

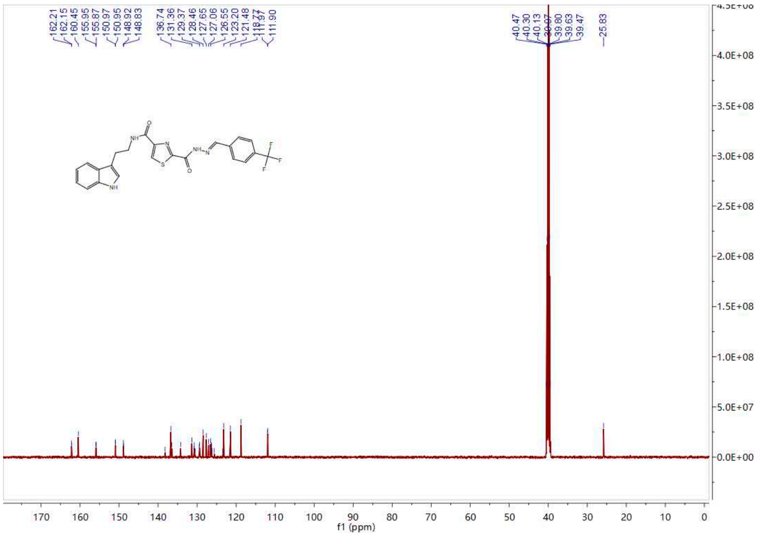

Figure S38 ^{13}C NMR of **BAD-13**

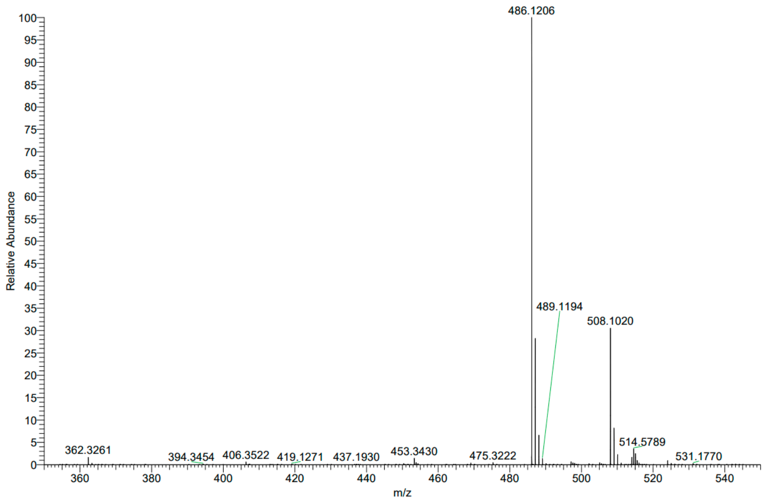

Figure S39 HRMS of **BAD-13**

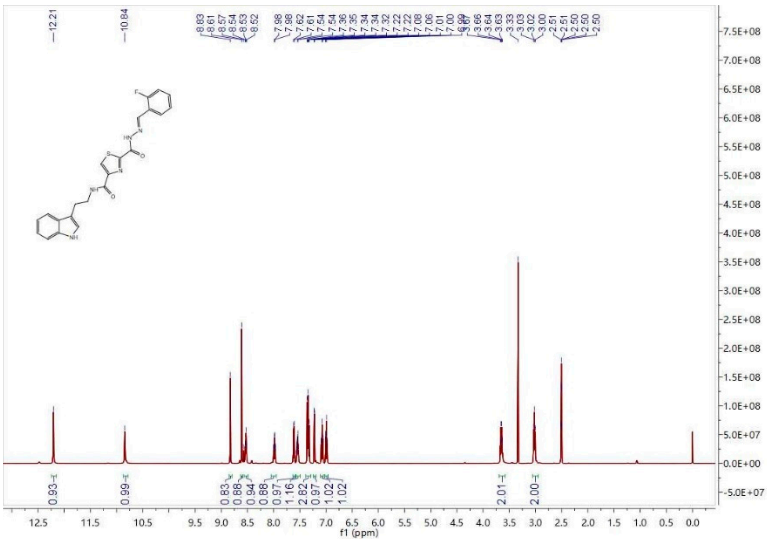

Figure S40 ^{1}H NMR of **BAD-14**

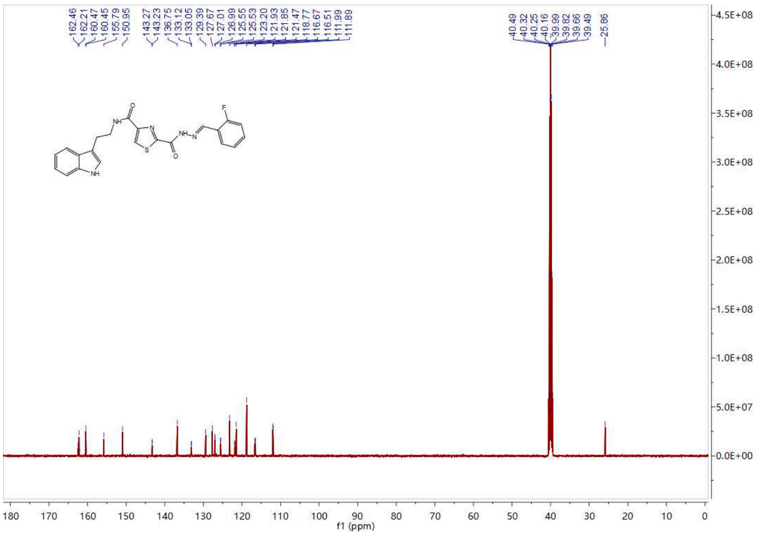

Figure S41 ^{13}C NMR of **BAD-14**

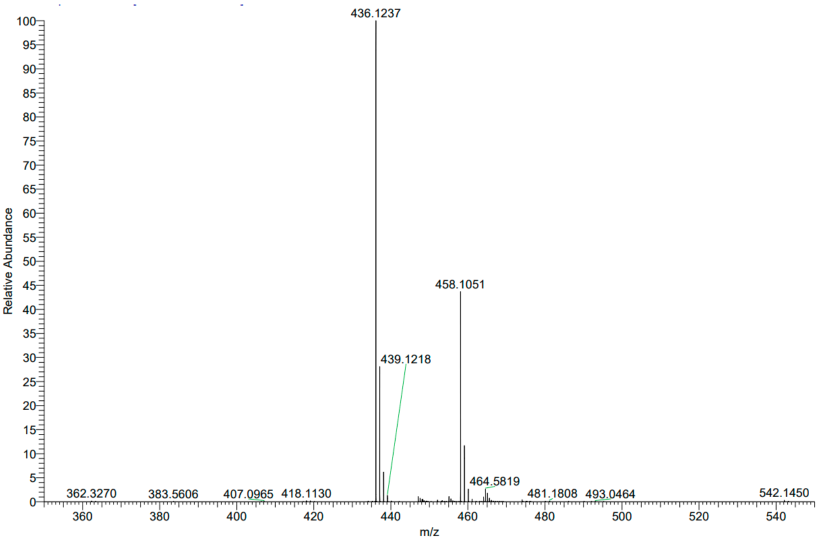

Figure S42 HRMS of **BAD-14**

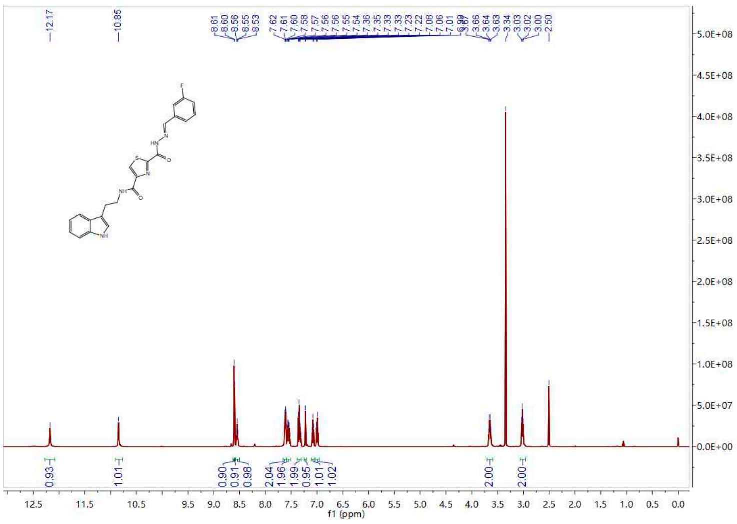

Figure S43 ^{1}H NMR of **BAD-15**

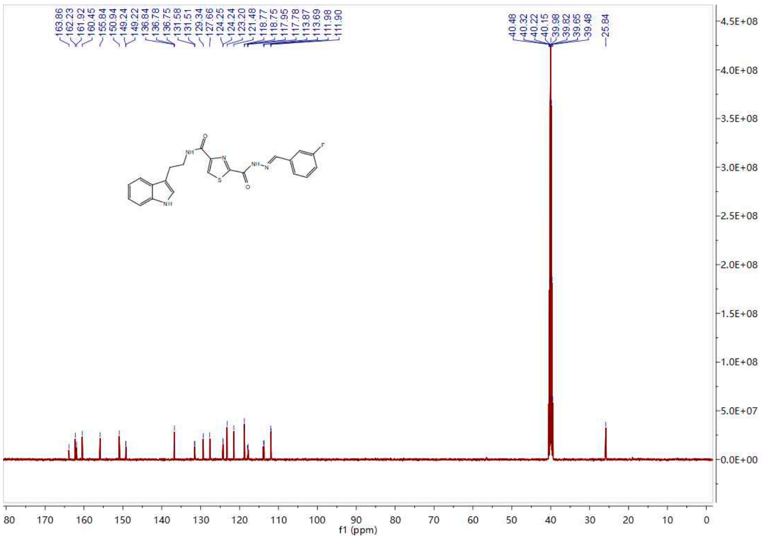

Figure S44 ^{13}C NMR of **BAD-15**

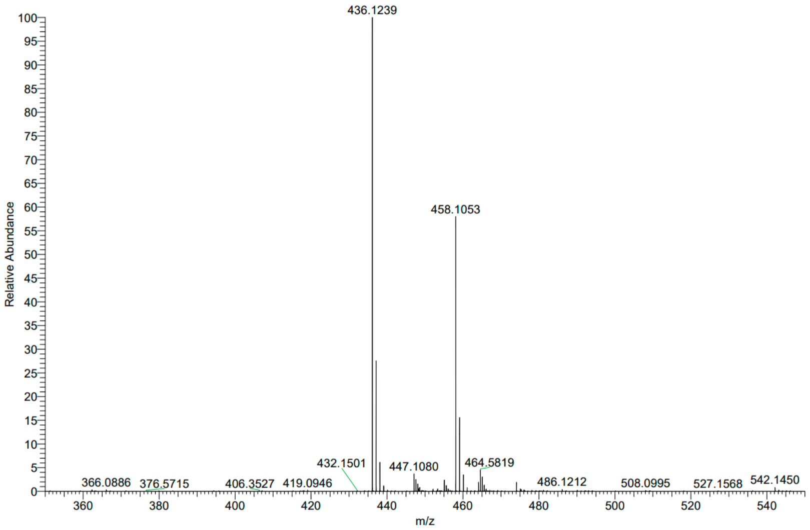

Figure S45 HRMS of **BAD-15**

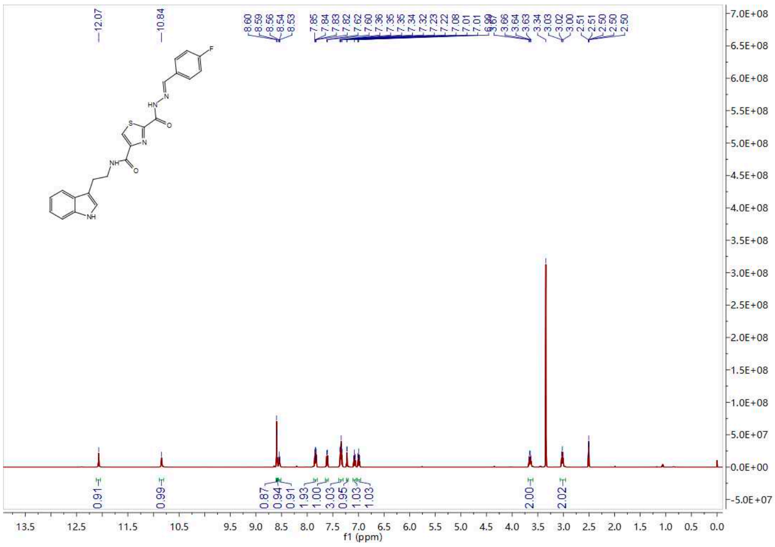

Figure S46 ^{1}H NMR of **BAD-16**

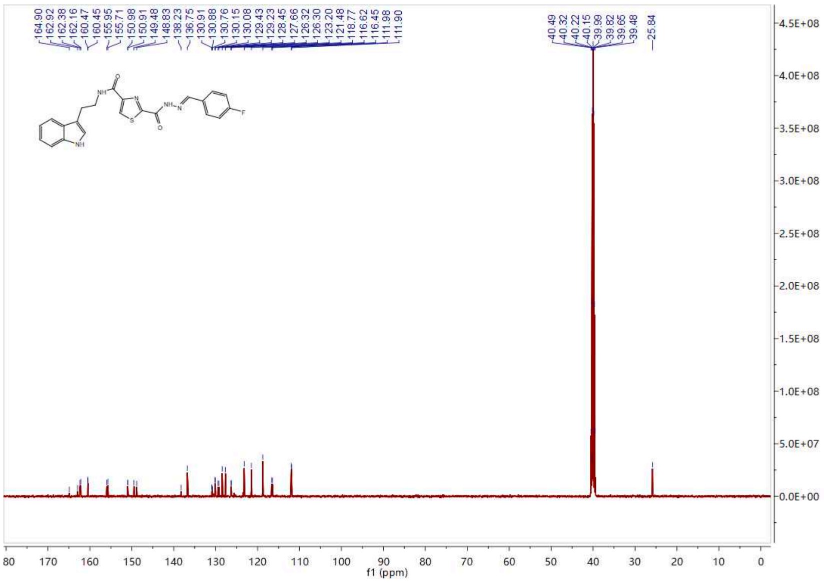

Figure S47 ^{13}C NMR of **BAD-16**

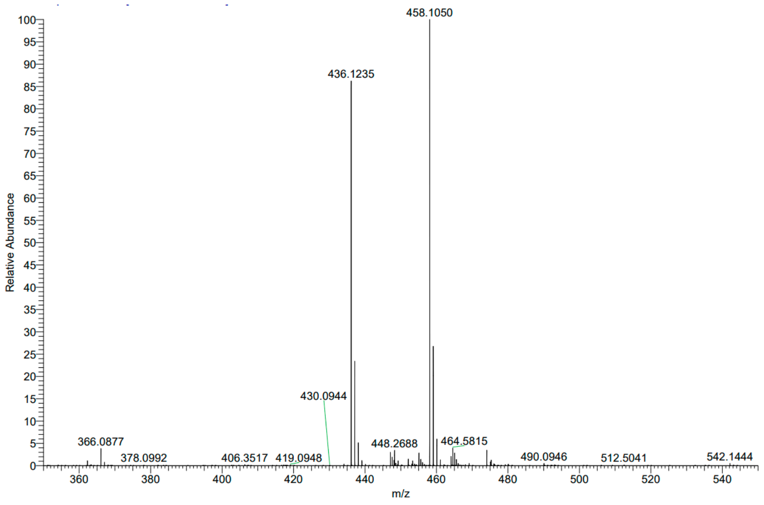

Figure S48 HRMS of **BAD-16**

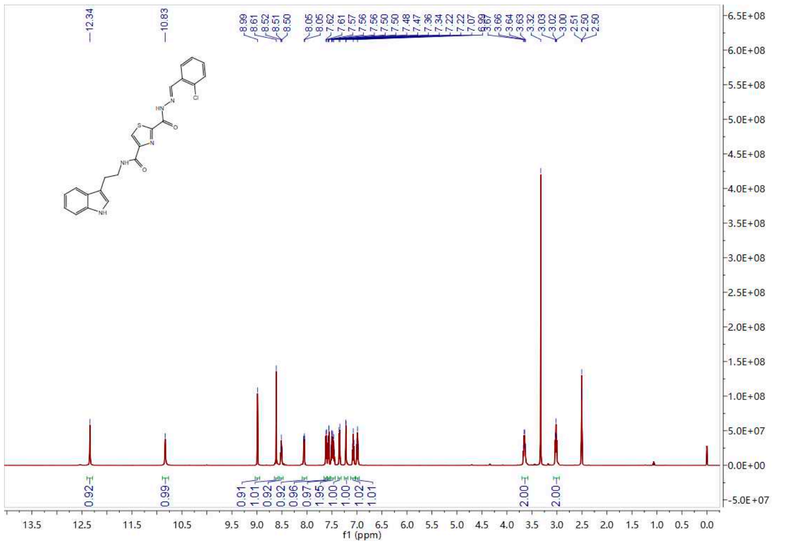

Figure S49 ^{1}H NMR of **BAD-17**

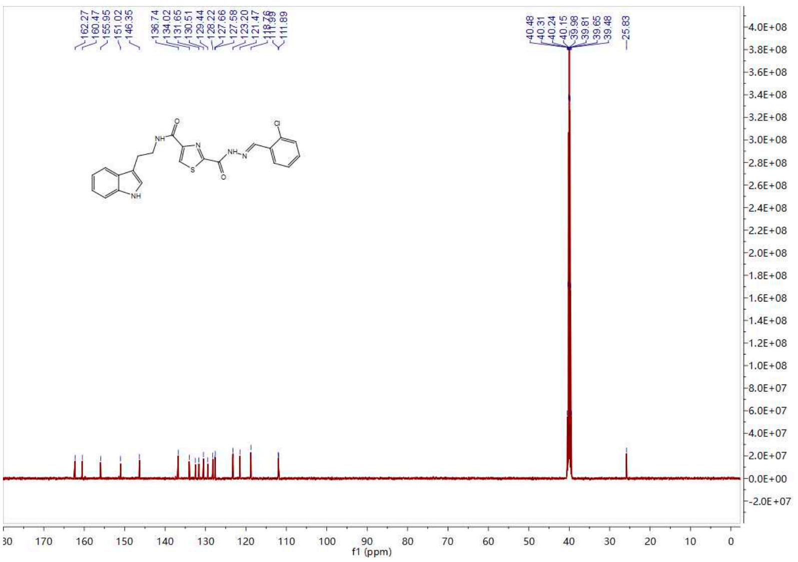

Figure S50 ^{13}C NMR of **BAD-17**

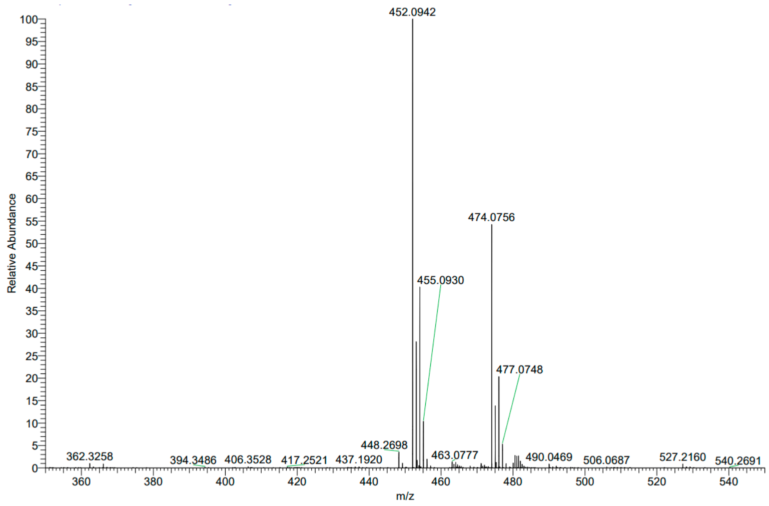

Figure S51 HRMS of **BAD-17**

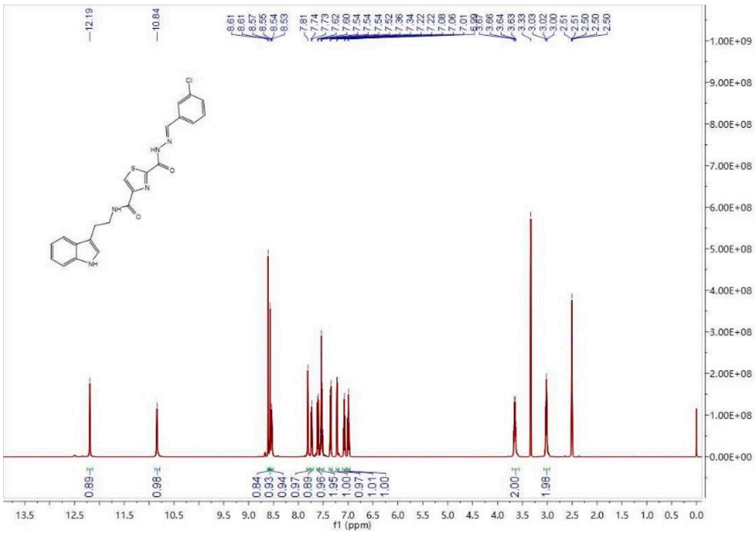

Figure S52 ^{1}H NMR of **BAD-18**

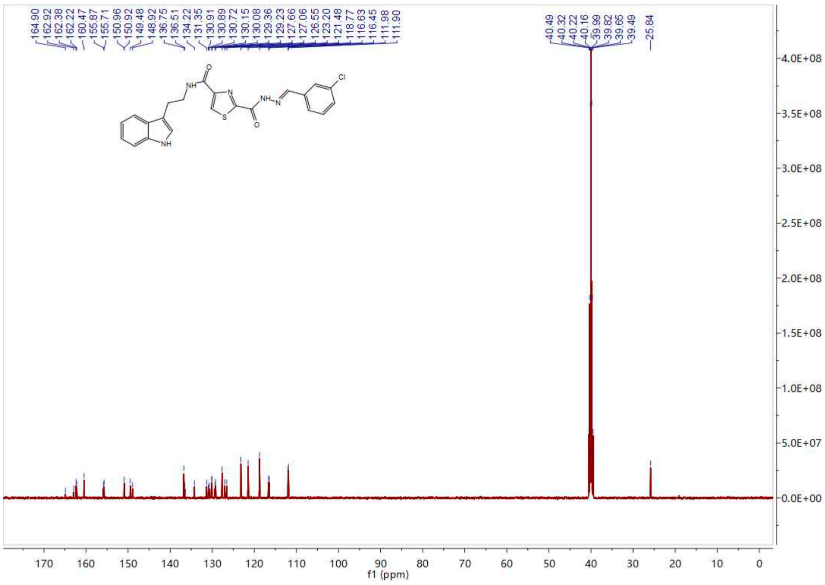

Figure S53 ^{13}C NMR of **BAD-18**

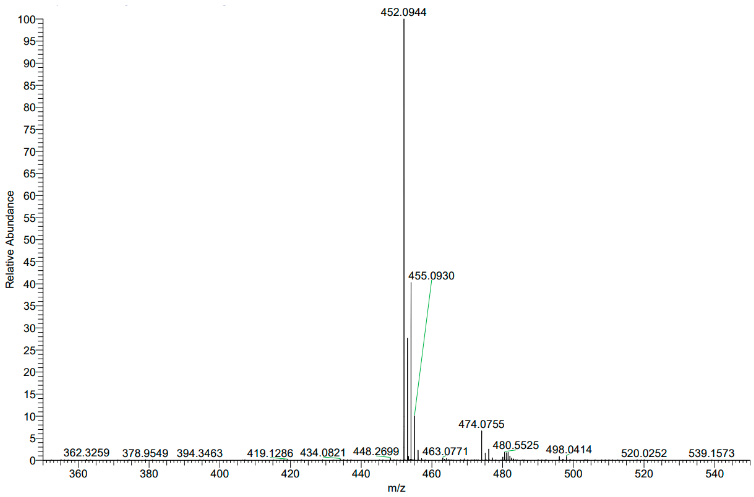

Figure S54 HRMS of **BAD-18**

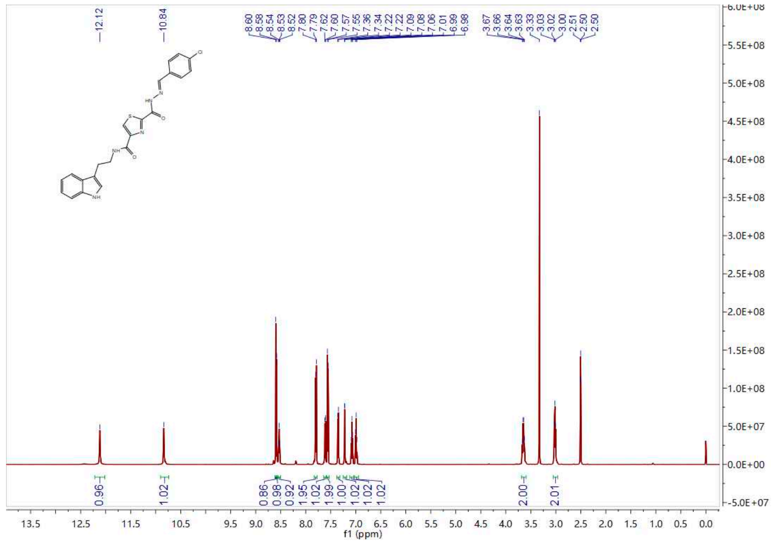

Figure S55 ^{1}H NMR of **BAD-19**

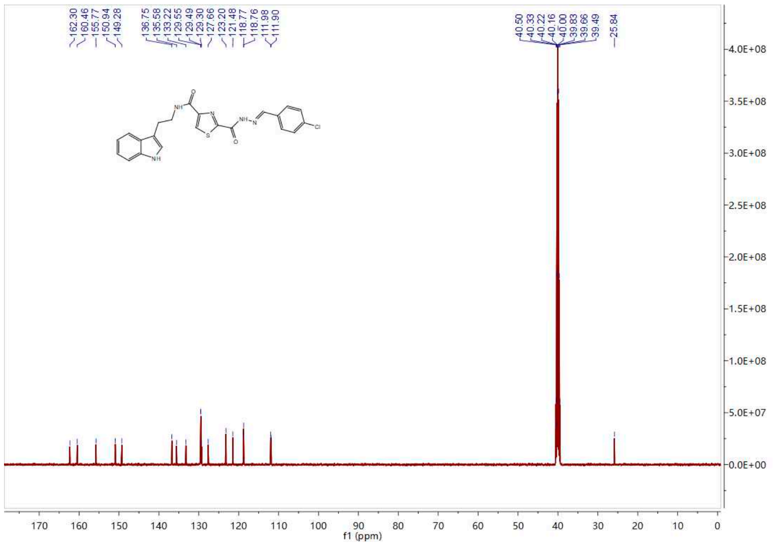

Figure S56 ^{13}C NMR of **BAD-19**

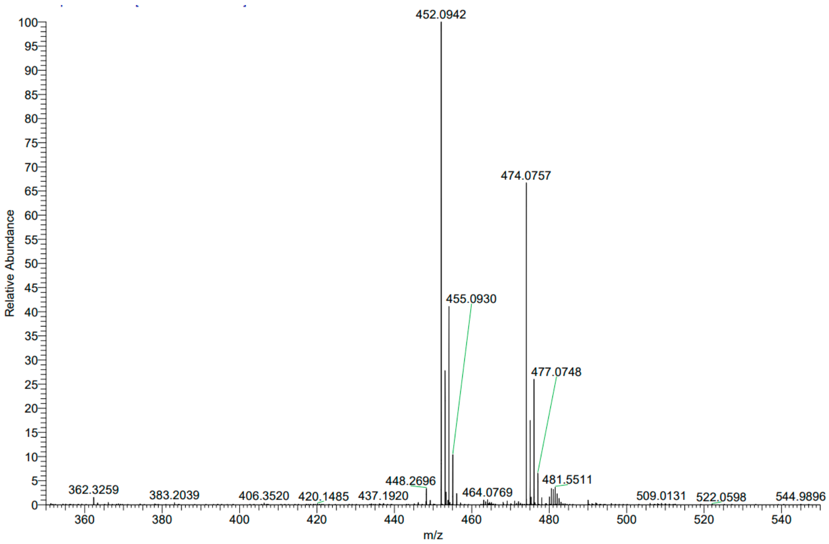

Figure S57 HRMS of **BAD-19**

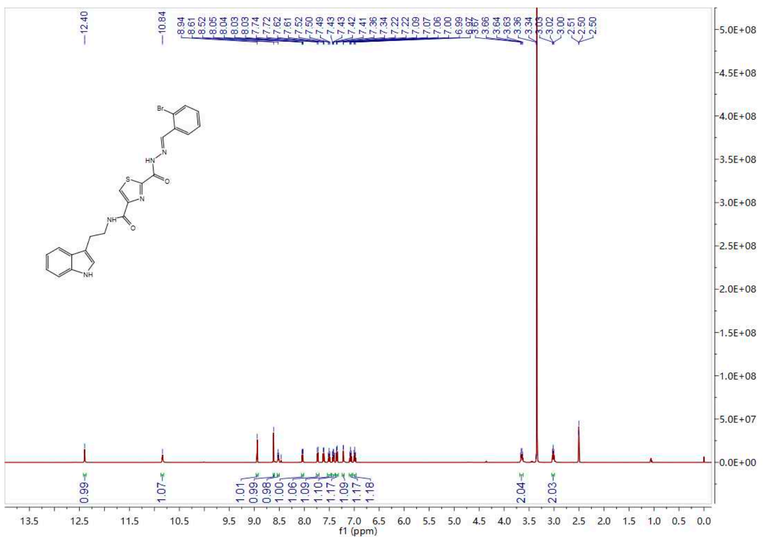

Figure S58 ^{1}H NMR of **BAD-20**

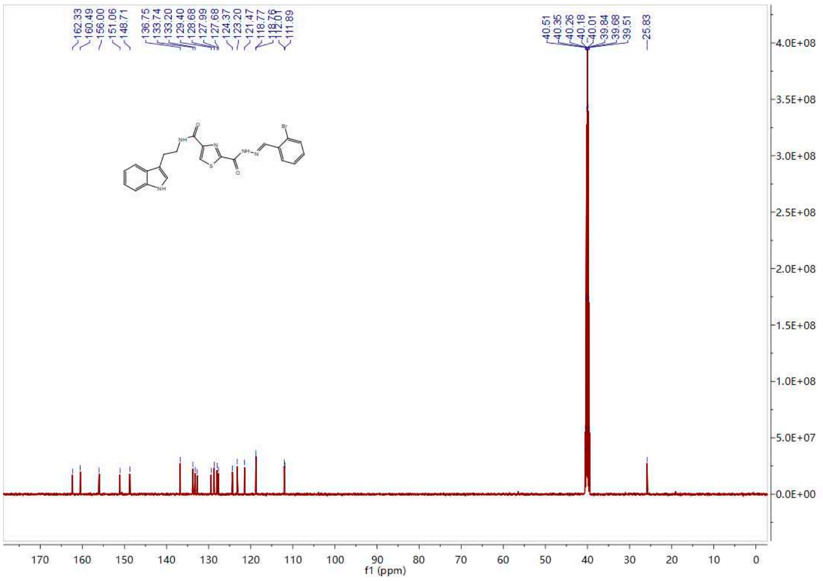

Figure S59 ^{13}C NMR of **BAD-20**

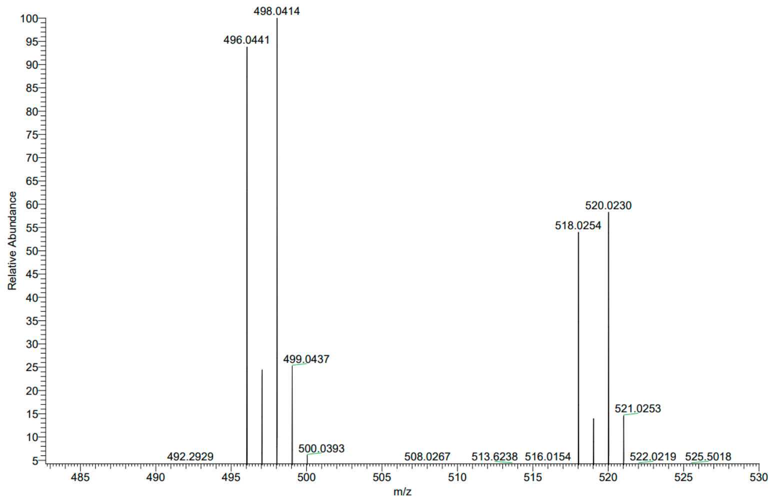

Figure S60 HRMS of **BAD-20**

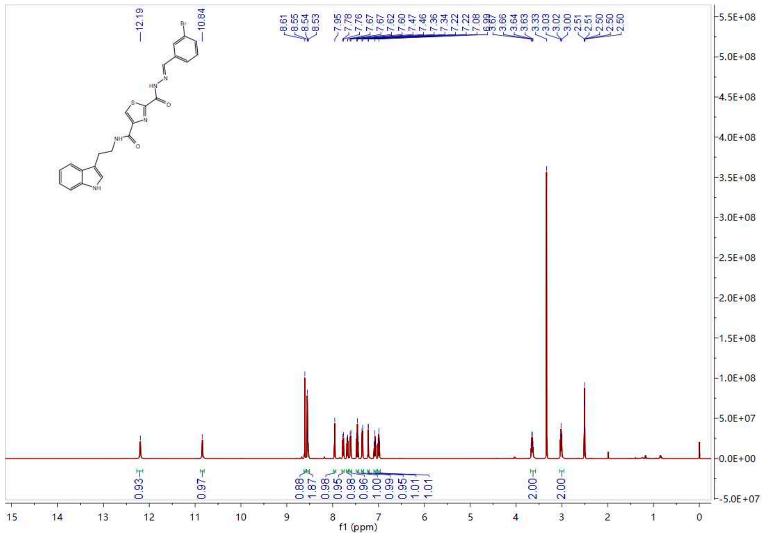

Figure S61 ^{1}H NMR of **BAD-21**

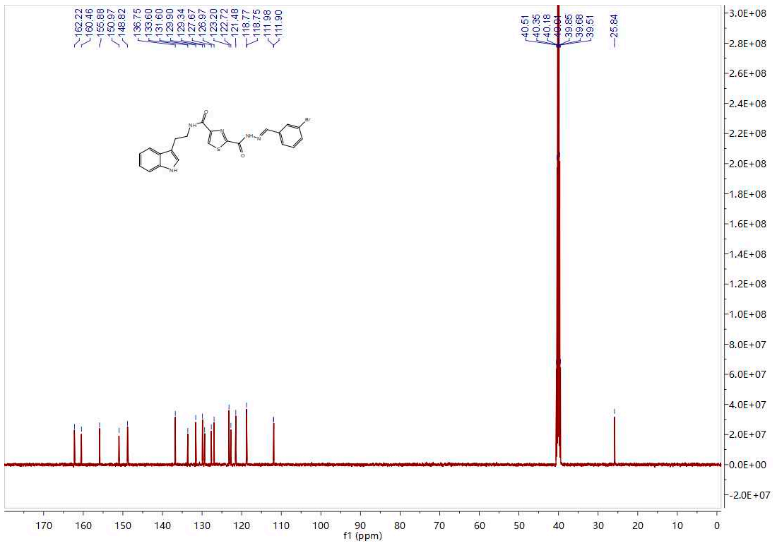

Figure S62 ^{13}C NMR of **BAD-21**

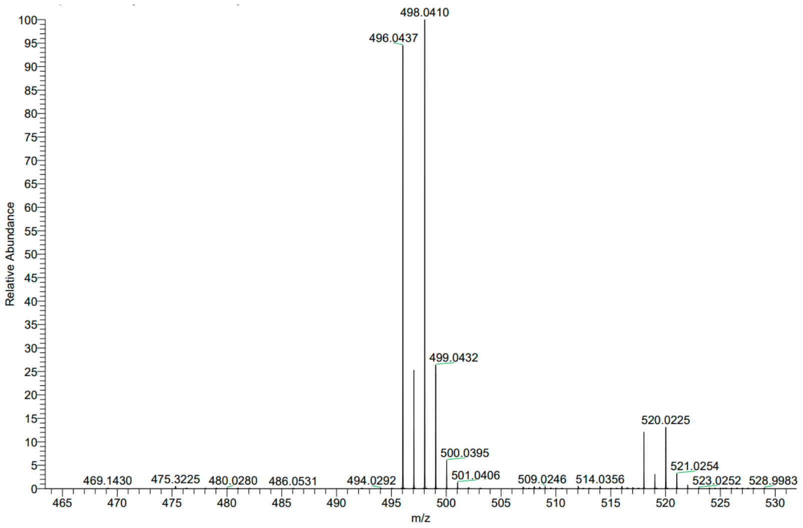

Figure S63 HRMS of **BAD-21**

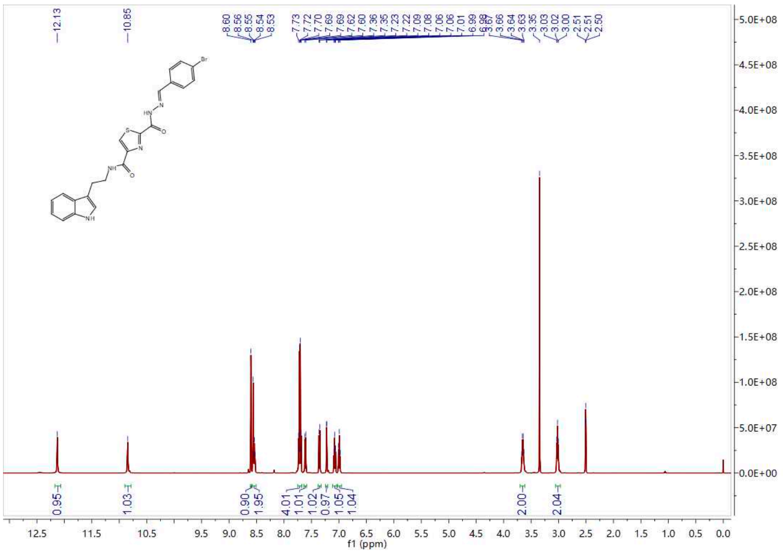

Figure S64 ^{1}H NMR of **BAD-22**

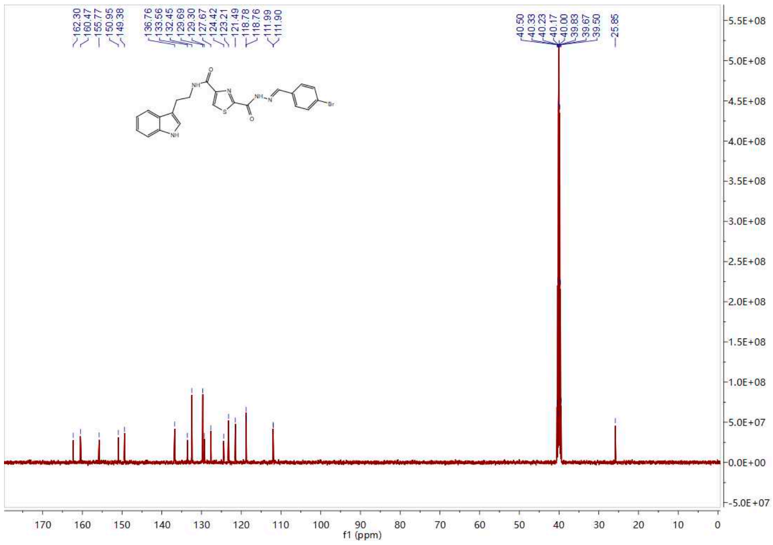

Figure S65 ^{13}C NMR of **BAD-22**

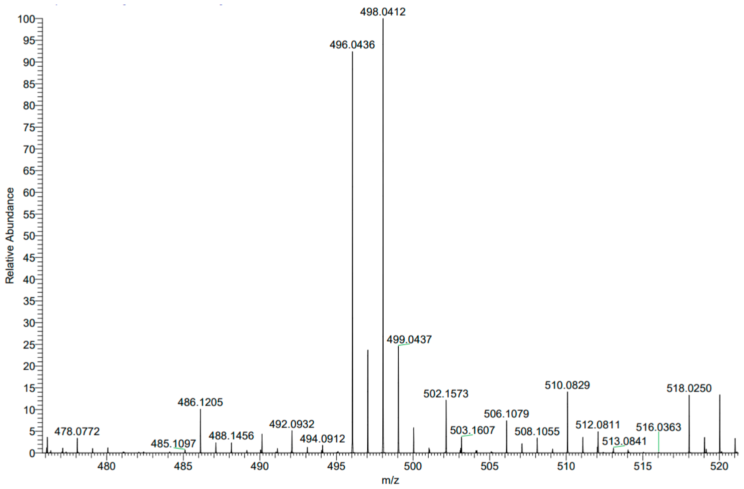

Figure S66 HRMS of **BAD-22**

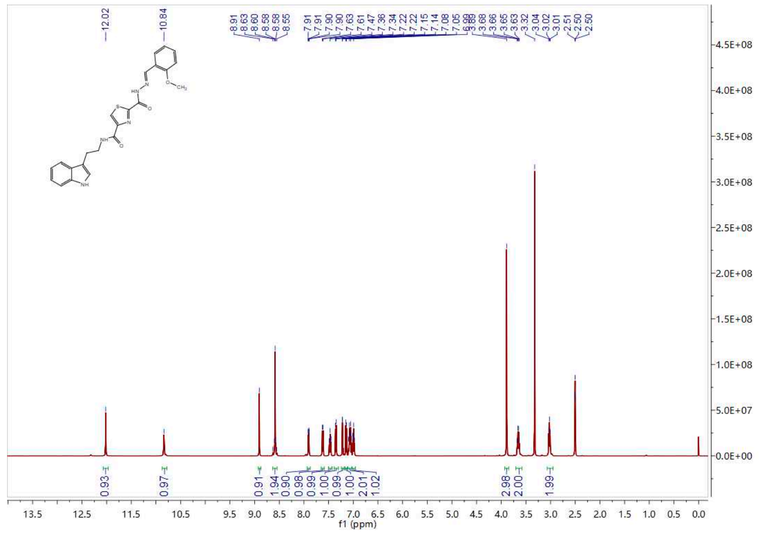

Figure S67 ^{1}H NMR of **BAD-23**

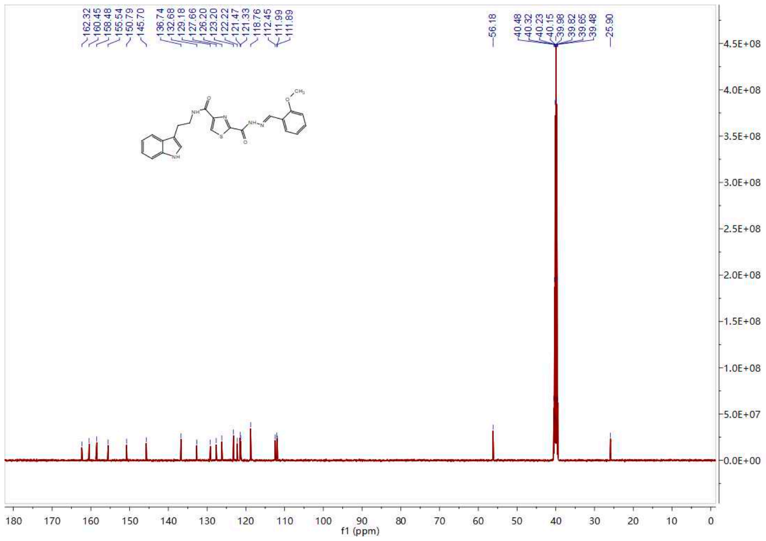

Figure S68 ^{13}C NMR of **BAD-23**

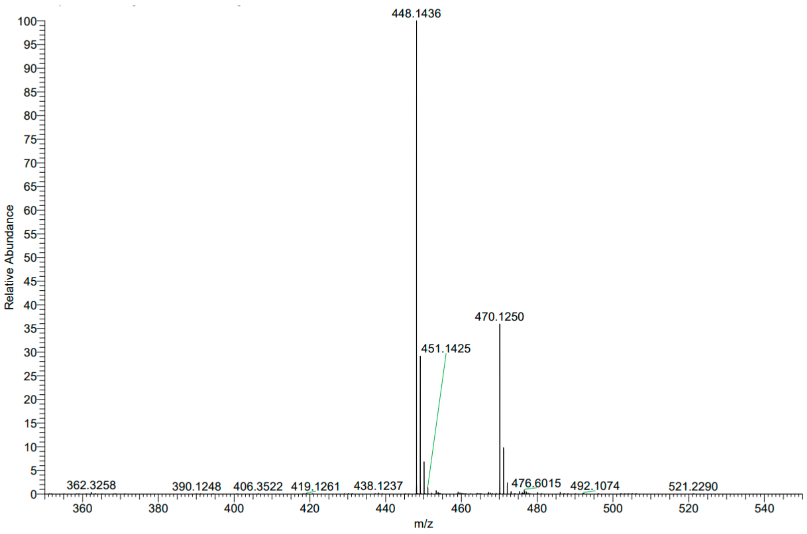

Figure S69 HRMS of **BAD-23**

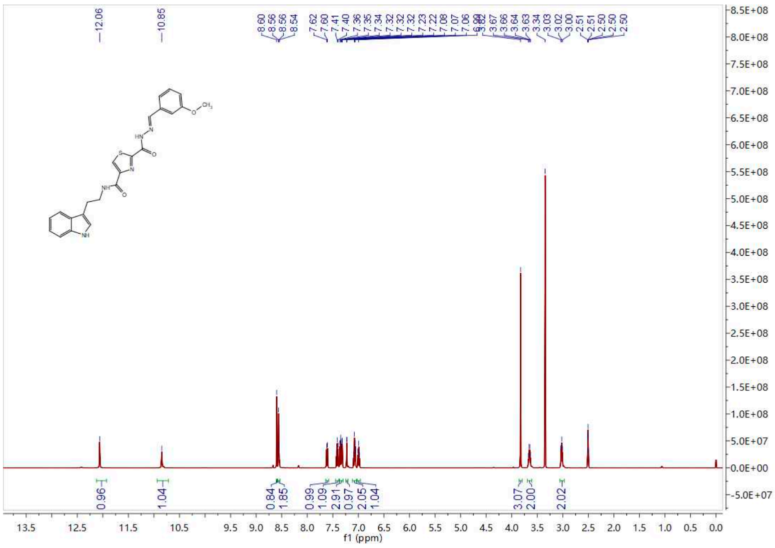

Figure S70 ^{1}H NMR of **BAD-24**

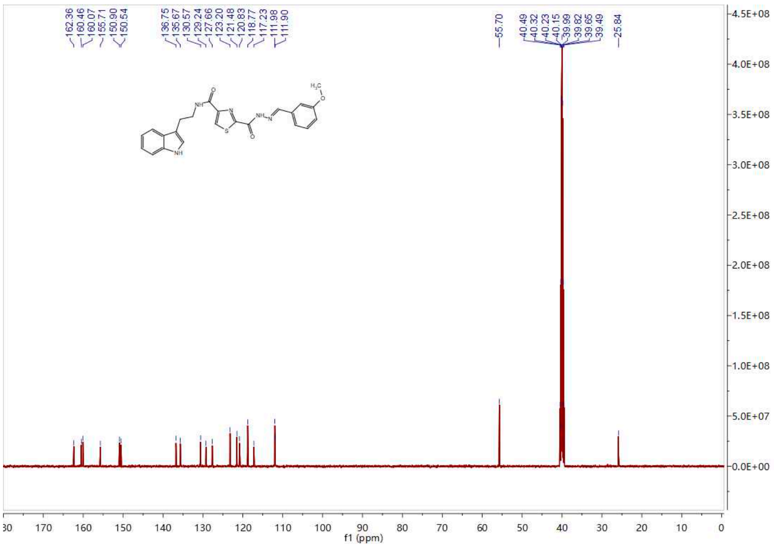

Figure S71 ^{13}C NMR of **BAD-24**

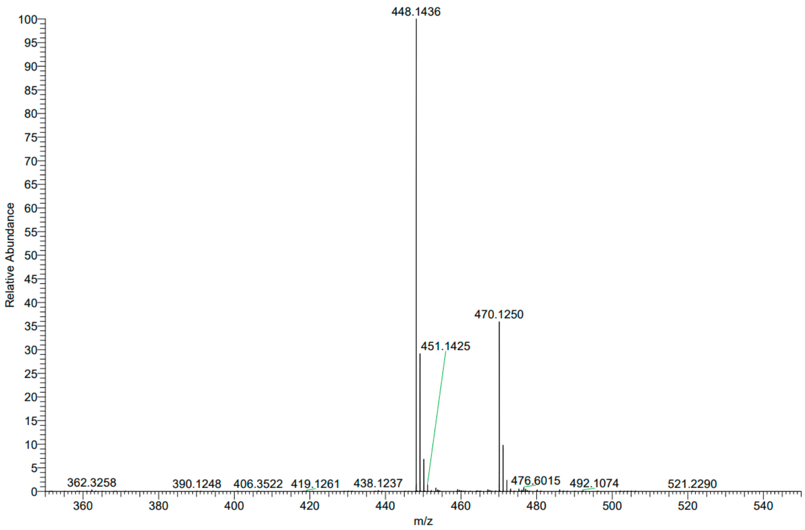

Figure S72 HRMS of **BAD-24**

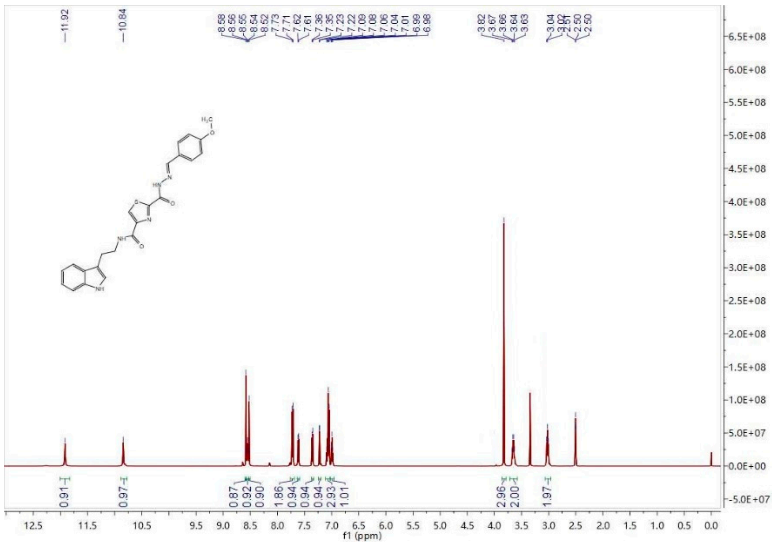

Figure S73 ^{1}H NMR of **BAD-25**

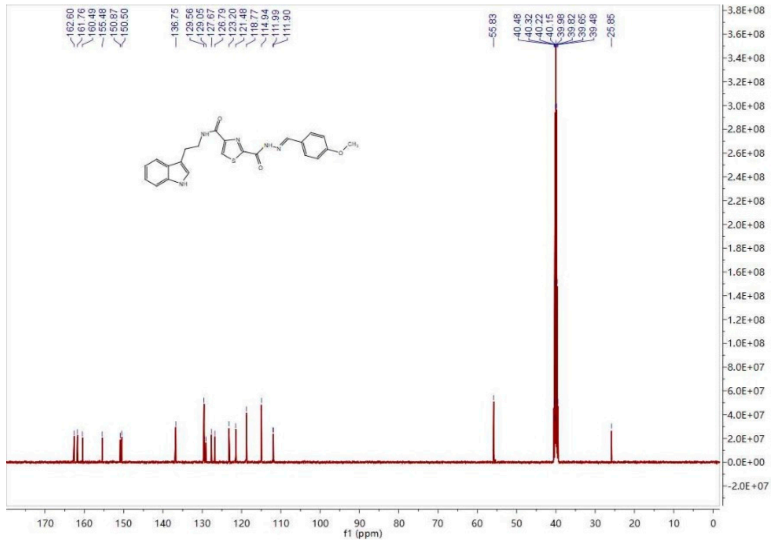

Figure S74 ^{13}C NMR of **BAD-25**

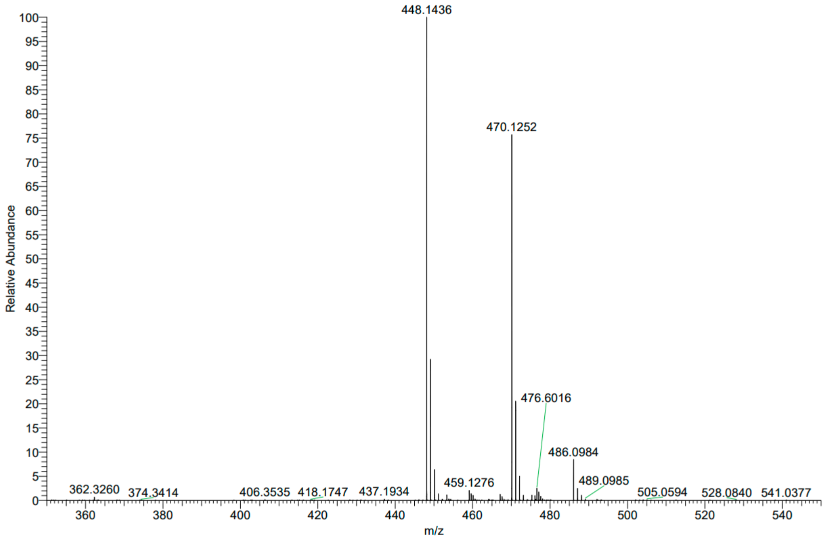

Figure S75 HRMS of **BAD-25**

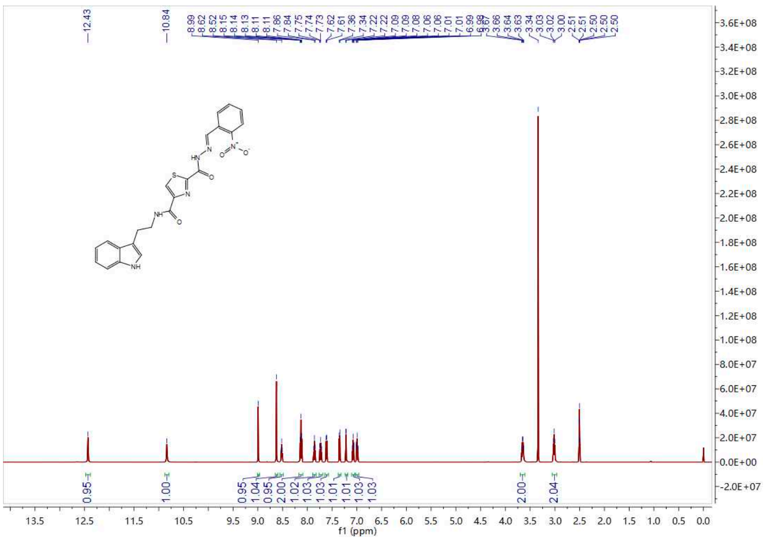

Figure S76 ^{1}H NMR of **BAD-26**

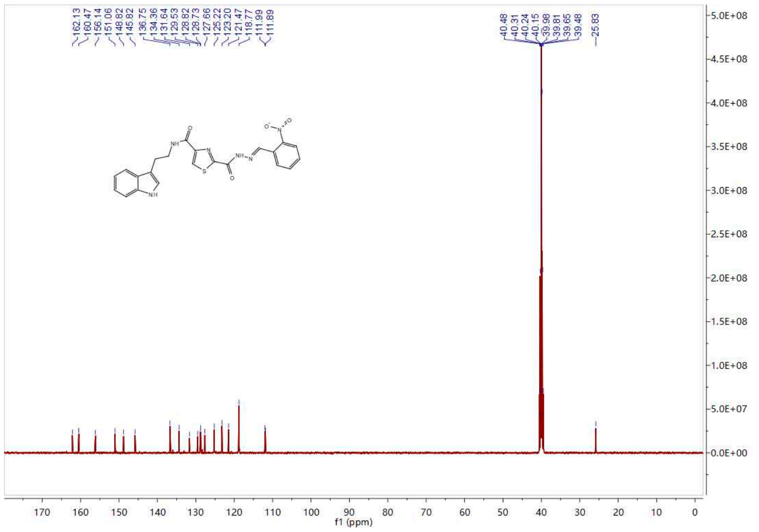

Figure S77 ^{13}C NMR of **BAD-26**

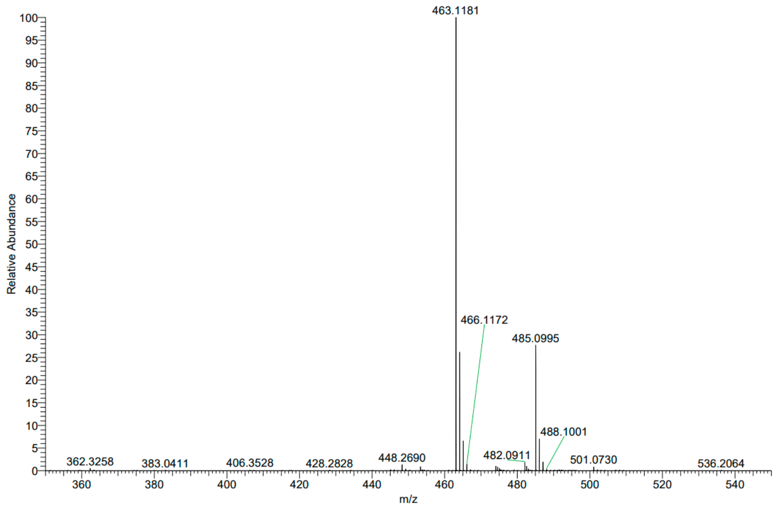

Figure S78 HRMS of **BAD-26**

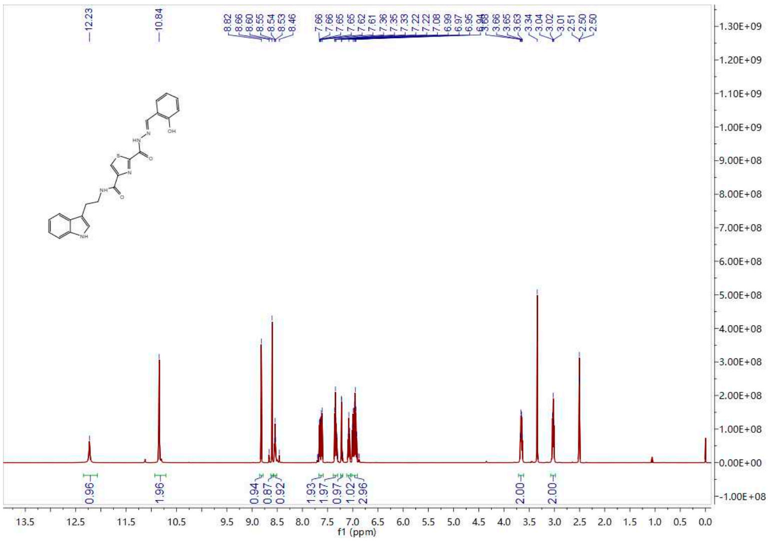

Figure S79 ^{1}H NMR of **BAD-27**

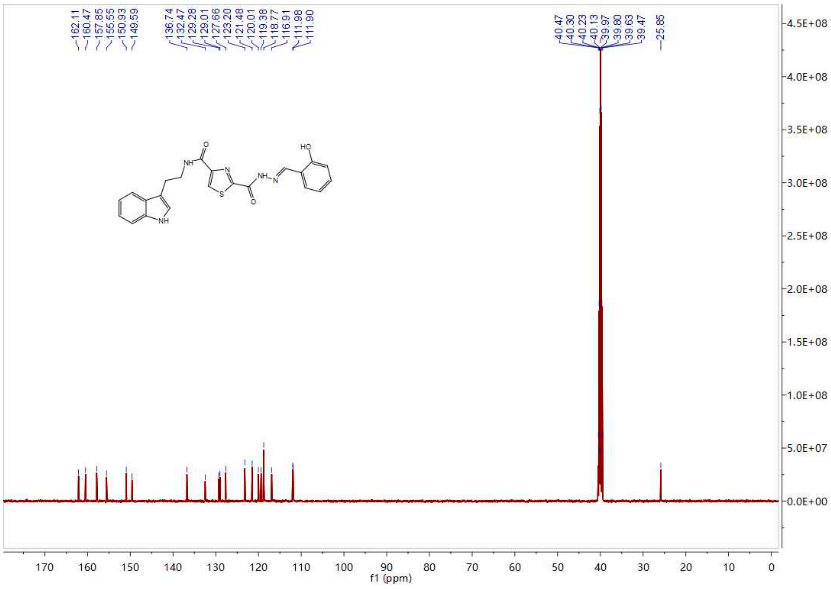

Figure S80 ^{13}C NMR of **BAD-27**

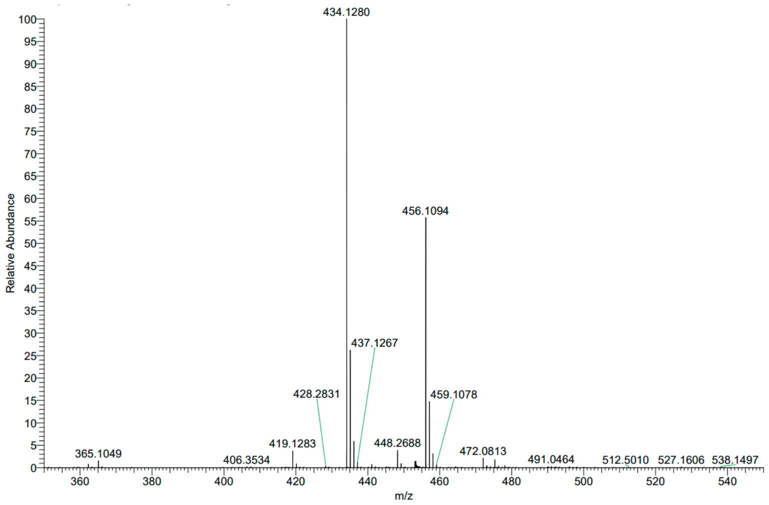

Figure S81 HRMS of **BAD-27**

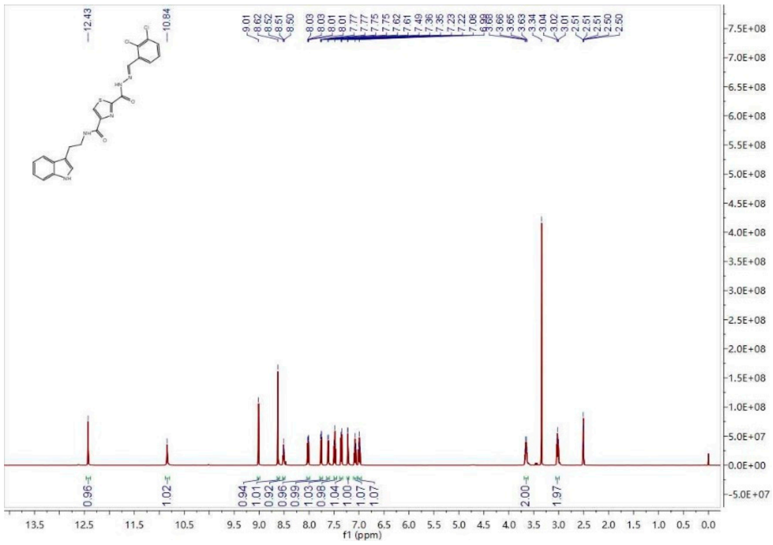

Figure S82 ^{1}H NMR of **BAD-28**

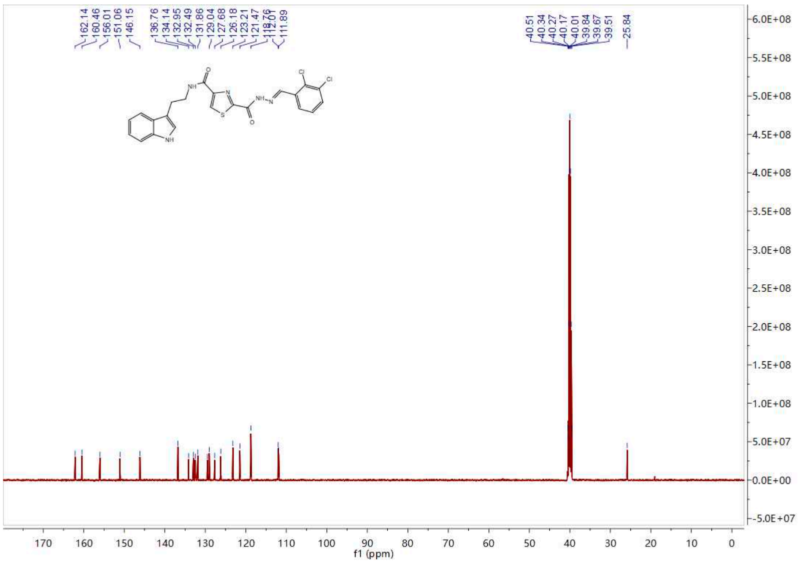

Figure S83 ^{13}C NMR of **BAD-28**

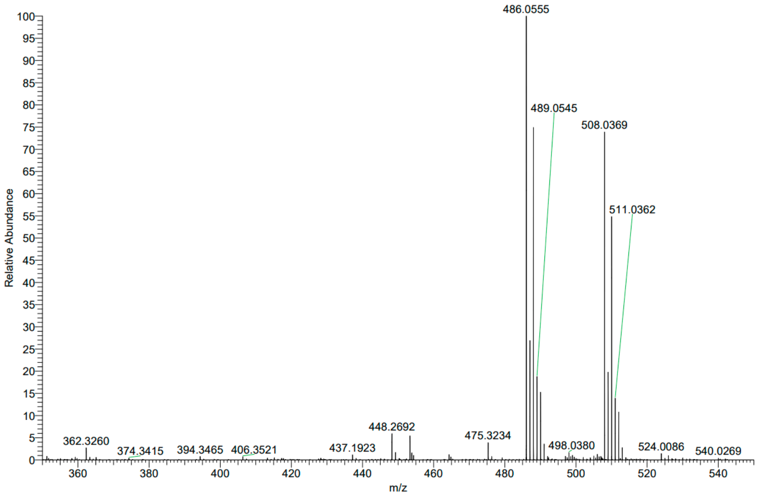

Figure S84 HRMS of **BAD-28**

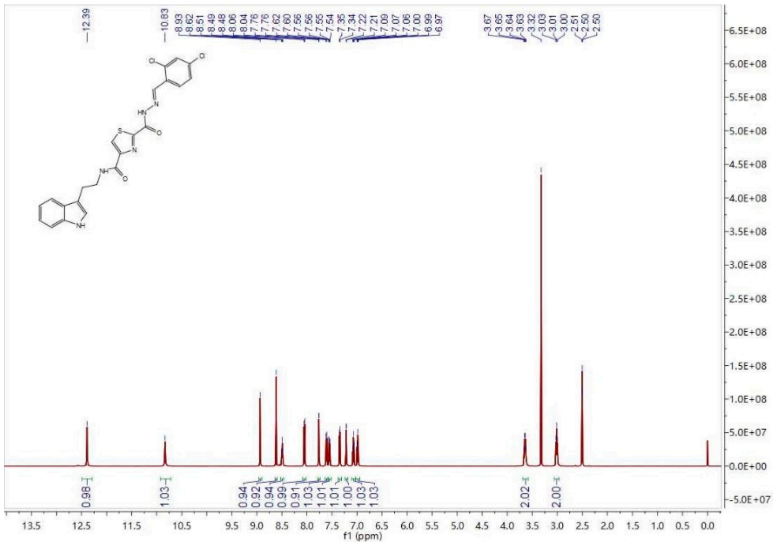

Figure S85 ^{1}H NMR of **BAD-29**

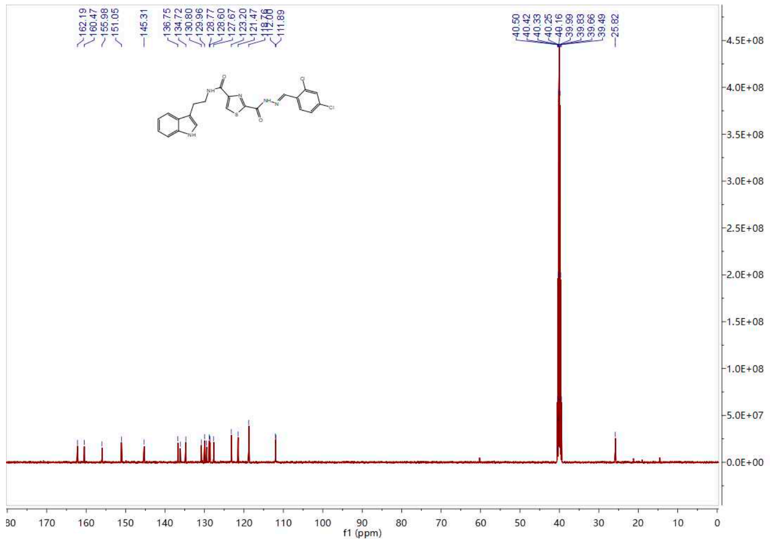

Figure S86 ^{13}C NMR of **BAD-29**

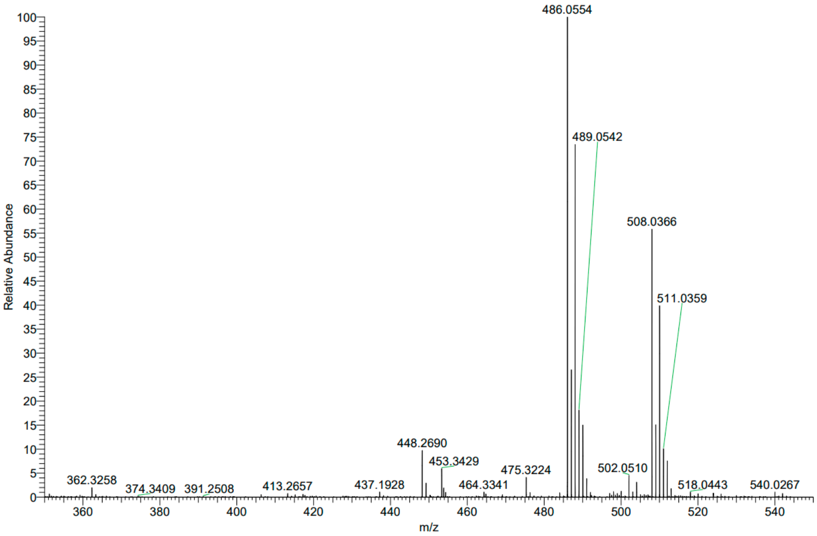

Figure S87 HRMS of **BAD-29**

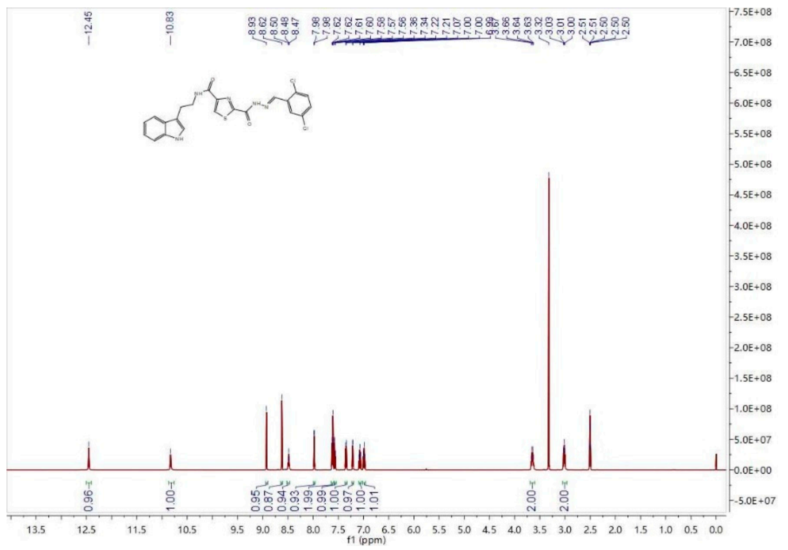

Figure S88 ¹H NMR of **BAD-30**

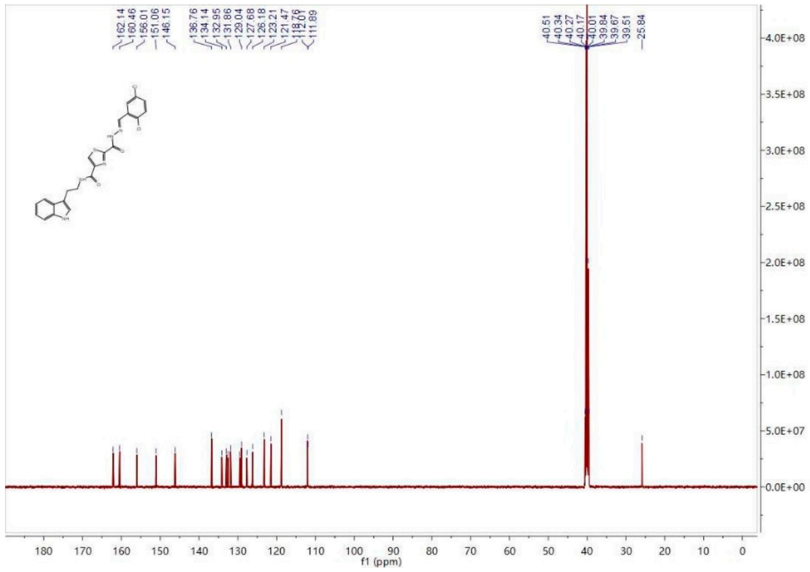

Figure S89 ¹³C NMR of **BAD-30**

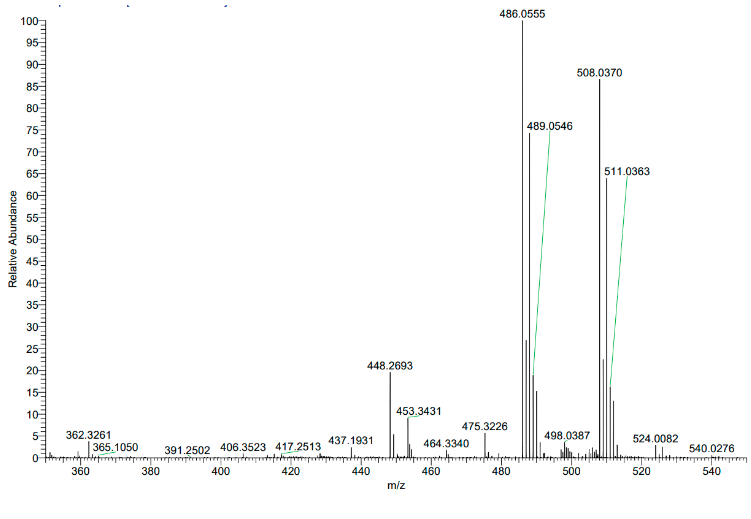

Figure S90 HRMS of **BAD-30**

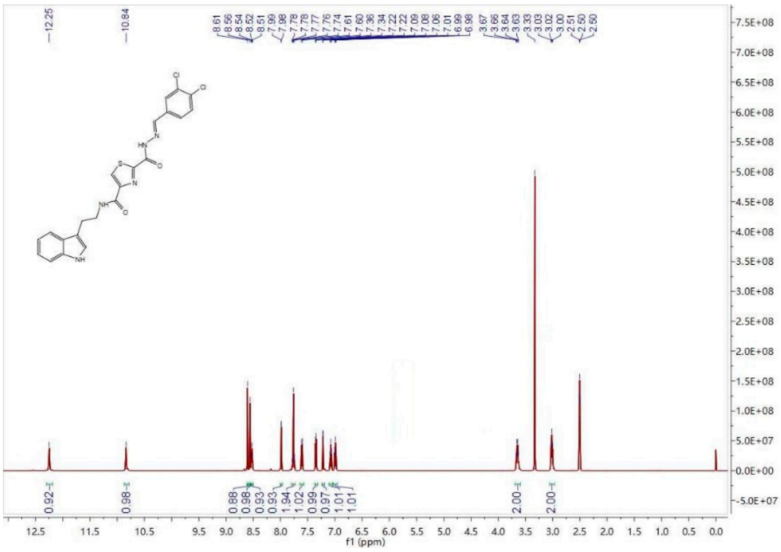

Figure S91 ^{1}H NMR of **BAD-31**

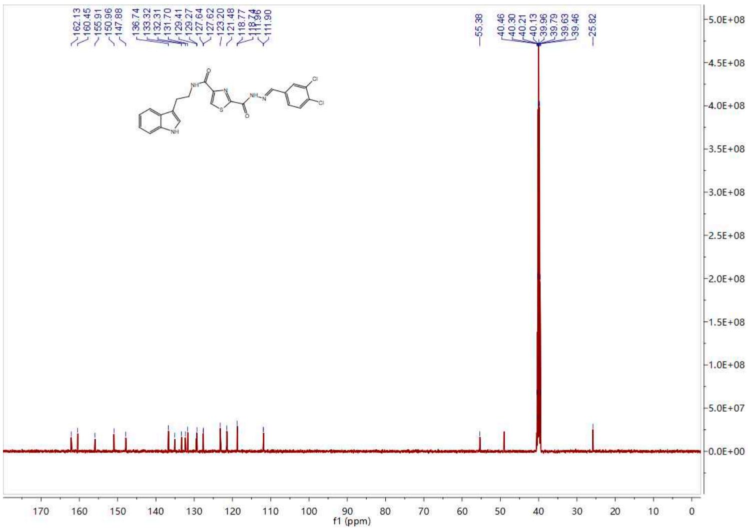

Figure S92 ^{13}C NMR of **BAD-31**

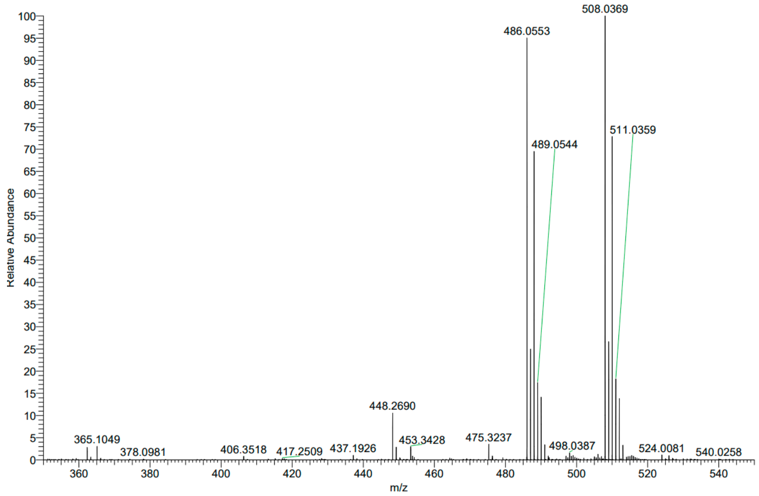

Figure S93 HRMS of **BAD-31**

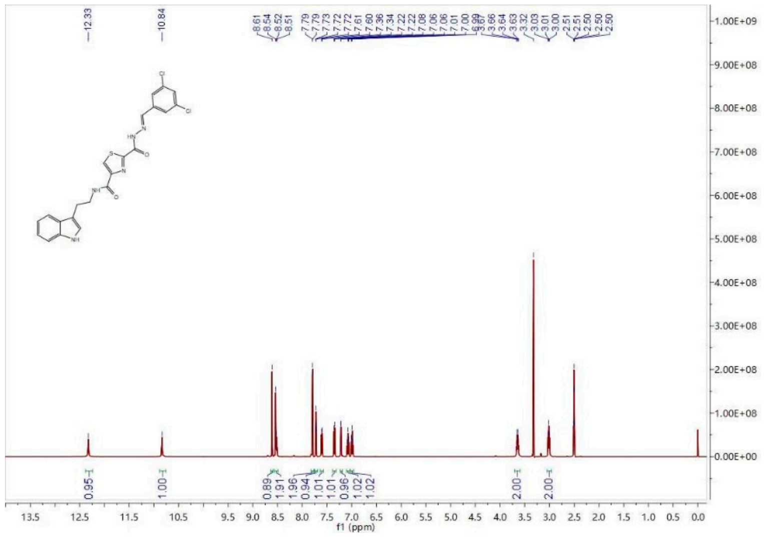

Figure S94 ^{1}H NMR of **BAD-32**

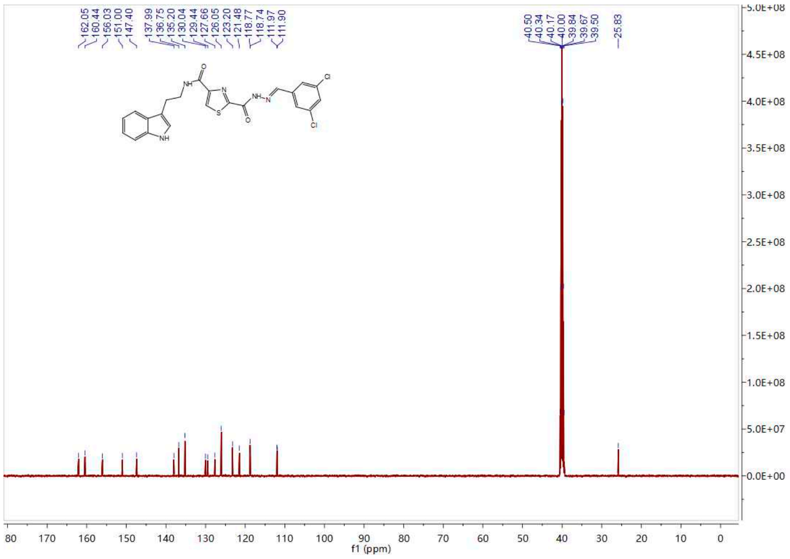

Figure S95 ^{13}C NMR of **BAD-32**

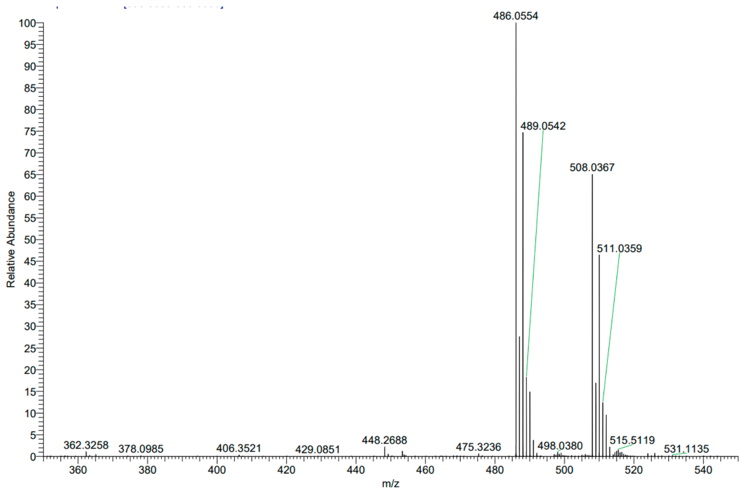

Figure S96 HRMS of **BAD-32**

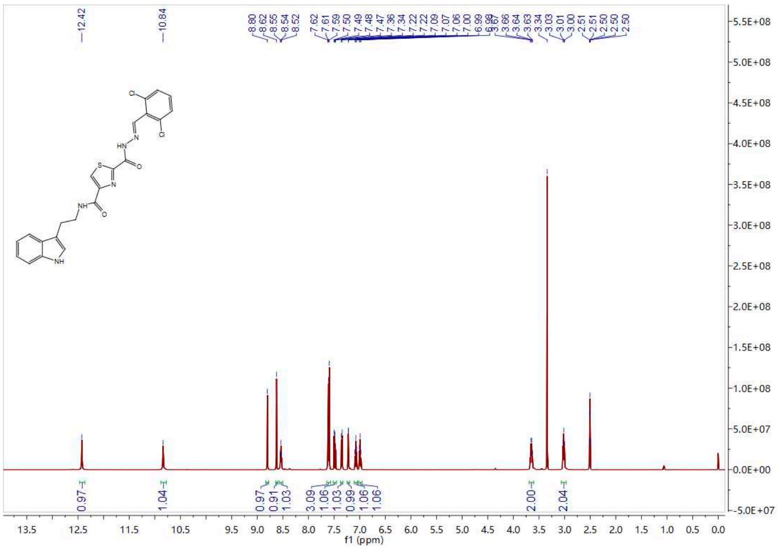

Figure S97 ^{1}H NMR of **BAD-33**

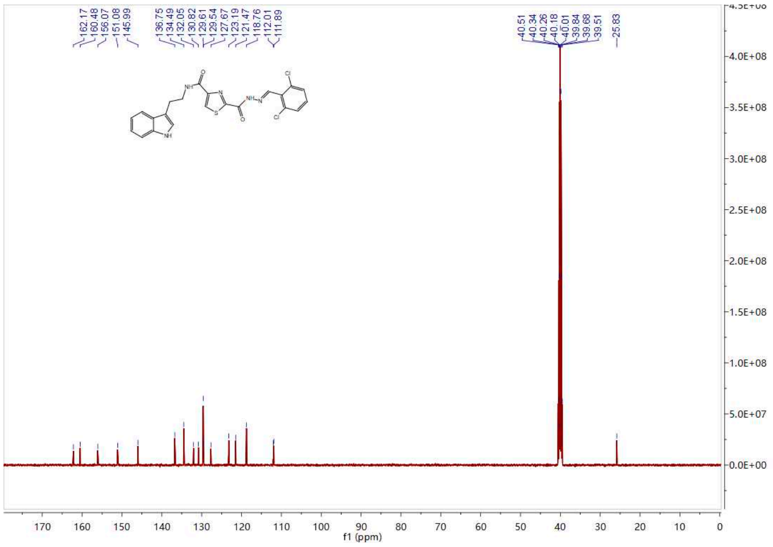

Figure S98 ^{13}C NMR of **BAD-33**

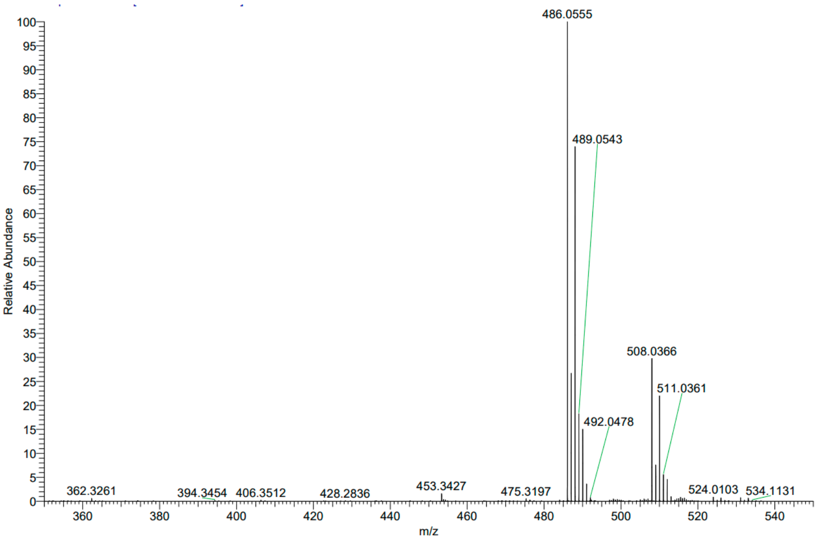

Figure S99 HRMS of **BAD-33**
